# Systematic review and meta-analysis of respiratory viral triggers for acute myocardial infarction and stroke

**DOI:** 10.1093/cvr/cvaf092

**Published:** 2025-06-17

**Authors:** Tu Q Nguyen, Diana Vlasenko, Aishwarya N Shetty, Eric Zhao, Christopher M Reid, Hazel J Clothier, Jim P Buttery

**Affiliations:** Department of Paediatrics, University of Melbourne, 50 Flemington Road, Parkville, Victoria 3052, Australia; Centre for Health Analytics and Epidemiology-Informatics Research Group, Murdoch Children's Research Institute, 50 Flemington Road, Parkville, Victoria 3052, Australia; Centre for Health Analytics and Epidemiology-Informatics Research Group, Murdoch Children's Research Institute, 50 Flemington Road, Parkville, Victoria 3052, Australia; Centre for Health Analytics and Epidemiology-Informatics Research Group, Murdoch Children's Research Institute, 50 Flemington Road, Parkville, Victoria 3052, Australia; Centre for Health Analytics and Epidemiology-Informatics Research Group, Murdoch Children's Research Institute, 50 Flemington Road, Parkville, Victoria 3052, Australia; School of Population Health, Curtin University, Bentley, Western Australia, Australia; Centre for Cardiovascular Research and Education in Therapeutics, Monash University, Melbourne, Victoria, Australia; Department of Paediatrics, University of Melbourne, 50 Flemington Road, Parkville, Victoria 3052, Australia; Centre for Health Analytics and Epidemiology-Informatics Research Group, Murdoch Children's Research Institute, 50 Flemington Road, Parkville, Victoria 3052, Australia; School of Population and Global Health, University of Melbourne, Parkville, Victoria, Australia; Department of Paediatrics, University of Melbourne, 50 Flemington Road, Parkville, Victoria 3052, Australia; Centre for Health Analytics and Epidemiology-Informatics Research Group, Murdoch Children's Research Institute, 50 Flemington Road, Parkville, Victoria 3052, Australia; Department of Infectious Diseases, Royal Children’s Hospital, Parkville, Victoria, Australia

**Keywords:** Acute myocardial infarction, Stroke, Respiratory virus, Vaccine preventable, Systematic review

## Abstract

Respiratory viral infections may trigger acute cardiovascular events. However, relative pathogen-specific associations are poorly understood, limiting optimal preventive recommendations. The aim of this study was to systematically review the association between respiratory viruses with two primary outcomes, acute myocardial infarction (AMI) and stroke. We searched MEDLINE, PubMed, Embase, Cochrane, and Web of Science, from database inception to 26 August 2024. Analytical epidemiological studies of respiratory viruses identified by laboratory-confirmatory testing, involving human participants of any age in any country, were eligible for inclusion. Risk of bias was assessed using the Cochrane Collaboration approach. Data from studies of sufficient quality and homogeneity were pooled using a random-effects model. Certainty of the evidence was assessed for each identified viral trigger. Of 11 017 articles identified, we included a total of 48 studies published between 1978 and 2024. All were observational studies, of which 28 were suitable for quantitative synthesis. There was moderate-certainty evidence that influenza triggers AMI (incidence rate ratio, 5.37; 95% CI, 3.48–8.28; *I^2^* = 69.4%). We found high-certainty evidence that influenza triggers stroke—influenza was associated with a 4.7-fold increased risk of stroke within the first 28 days following infection (incidence rate ratio, 4.72; 95% CI, 3.78–5.90; *I^2^* = 0%). SARS-CoV-2 and cytomegalovirus may trigger stroke, while SARS-CoV-2, respiratory syncytial virus, and Coxsackie B were also identified as potential triggers for AMI. In this systematic review and meta-analysis, the findings suggest that common, often vaccine-preventable, respiratory viral infections are associated with an increased risk of acute cardiovascular events.

## Graphical Abstract

**Graphical Abstract:**
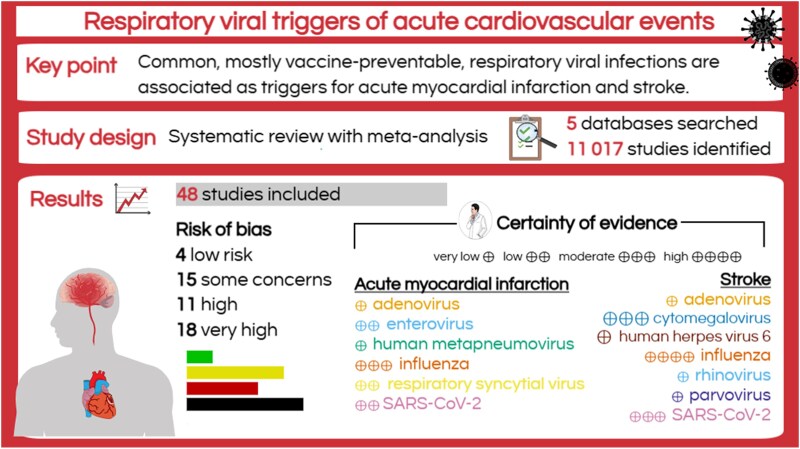

## 1. Introduction

Cardiovascular diseases contribute substantially to global disease burden, with ischaemic heart disease the single biggest contributor to disability of all diseases.^1^ Most cardiovascular disease burden is due to atherosclerotic disease, which presents acutely in multiple ischaemic or haemorrhagic clinical presentations, including acute myocardial infarction (AMI) and stroke.^2^ These two major acute cardiovascular events are estimated to account for approximately 85% of cardiovascular mortality.^3^ While most cardiovascular risk factors are well-established, not all of the attributable burden has been explained. It is generally accepted that *precipitating* or *triggering* risk factors, including stress, physical exertion, some medications, and infections, increase the short-term risk in individuals with *baseline underlying risk* to finally having an AMI or stroke.

With modern molecular diagnostic laboratory tests, there is now increasing evidence of the role of infectious agents as triggers for acute cardiovascular events.^4,5^ Although the exact mechanistic pathways are unclear, the prevailing theory is that atherosclerosis may be induced by activation of inflammatory biomarkers in the acute phase of infection.^6^ The risk window of impact on the cardiovascular system following infection appears to be transient, lasting from several days to few months,^7^ as opposed to identifiable long-term sequelae.

Understanding the relative associations of different pathogens with acute cardiovascular disease outcomes is important to inform population burden and guide preventive strategies.^8,9^ Much research has predominantly focused on influenza, owing to its severity and position as a pathogen for which vaccination or therapeutic intervention is available.^10,11^ Meanwhile, other respiratory pathogens may be implicated, such as herpes viruses linked with stroke and Coxsackie B linked with AMI.^8,9^ While improved population-level data have enabled large-scale epidemiological studies on respiratory virus detections and their role as triggering factors, the pathogen-specific contributions to the risk of AMI and stroke remain unclear. Previous systematic reviews and meta-analyses have been limited in scope, either focusing solely on a single viral agent like influenza or SARS-CoV-2, or have used broad, non-specific definitions of infection (e.g. influenza-like illness),^12^ making interpretation challenging as data were not compared across studies detecting different attributable viruses.

The relative effects of different respiratory viruses on the risk of acute cardiovascular events remain poorly understood, particularly when comparing seasonality, climate, and populations that differ between geographic areas.^13^ Research to date has not comprehensively examined the role of respiratory viral infections on the risk of AMI and stroke. Randomized trial evidence suggests that influenza vaccination may confer a cardiovascular protective effect, particularly among high-risk groups,^14^ while further trials with cardiovascular endpoints are ongoing. Therefore, it was postulated that comparison of various attributable viruses may help identify other candidate pathogens for potential vaccine trials and other preventive therapies in targeted populations.

Our primary objective was to systematically review evidence from analytical epidemiological studies, either randomized or non-randomized, of respiratory viruses identified by laboratory-confirmatory testing involving humans of all ages and geographical areas. We specifically focused on laboratory-confirmatory infections to compare the associations between viral respiratory triggers with no detected infection, and if appropriate, to estimate pathogen-specific pooled effect sizes with AMI and stroke events. A secondary objective was to identify evidence gaps, assess the certainty of the evidence, and recommend areas for future research. As no randomized controlled trials met inclusion criteria, this systematic review comprised entirely of observational studies.

## 2. Methods

### 2.1 Registration

This systematic review was registered with PROSPERO (CRD42024494997). We have published an *a priori* protocol^15^: important changes to the protocol are detailed in Supplementary material online, *Methods S1*.

### 2.2 Search strategy and data sources

A comprehensive search of epidemiological studies on laboratory-confirmed respiratory viral infections and their associations with AMI or stroke outcomes was performed. The search strategy was designed based on the Population, Exposure, Comparison, Outcome, Study design, and Timeframe (PECOST) framework using Boolean operators (AND, OR) to combine variations of the search concepts: *humans*, *laboratory confirmatory tests*, *respiratory viruses*, *acute myocardial infarction, stroke* from *analytical epidemiological study designs*. We searched five electronic bibliographic databases from their inception until 26 August 2024: MEDLINE (Ovid), EMBASE (Ovid), PubMed, Cochrane Central Register of Controlled Trials, and Web of Science (see Supplementary material online, *Methods S2*). In addition, we manually checked the reference lists of the included studies (backward citations), citing articles (forward citations), and of earlier systematic reviews to identify any further relevant studies.

### 2.3 Eligibility criteria

The eligibility criteria were defined according to the PECOST elements as follows: population (P)—studies involving human participants of any age in any country; exposure (E)—respiratory viral infection as detected by confirmatory laboratory methods. We defined exposures as those primarily resulting in respiratory phenotypes; comparator (C)—eligible studies included a comparator group that was unexposed (e.g. negative testing individuals, unexposed person-time); outcome (O)—AMI or stroke. Composite acute cardiovascular or cerebrovascular events, including AMI or stroke, but not exclusive to these conditions, were excluded. Studies were included if either or both conditions were measured; study design (S)—randomized controlled trials (RCTs), cohort and case-control studies, self-controlled case series (SCCS), and case crossover designs; timeframe (T)—studies using short-term risk periods (up to 90 days) were prioritized (see Supplementary material online, *Methods S3*).

Studies must have reported, or provided data to calculate, an estimate of the effect on the risk of AMI and/or stroke [i.e. risk ratio or rate ratio (RR), odds ratio (OR), incidence rate ratio (IRR), or hazard ratio (HR)]. There were no restrictions on the date, country, geographical area of study, language, population, or publication status. Due to the high specificity of eligibility criteria in terms of exposure, comparator, outcome, study design, and timeframe, the populations eligible for inclusion was relatively liberal, to minimize biases associated with selectively excluding specific subpopulations.^16^

### 2.4 Study selection

All search results were screened for eligibility by at least two independent reviewers in two stages: title and abstract screening, followed by full-text review. Discrepancies in either of the screening stages were resolved by consensus and/or arbitration by a third reviewer. We contacted the authors for clarification, or if further information was required for study selection.

### 2.5 Data collection and quality assessment

Data were extracted using a prepiloted, standardized template in Covidence. Outcome data were collated from the text, tables, or figures. The most fully adjusted measures were extracted. Where only raw data were reported, we extracted data (e.g. constructed 2 × 2 tables) to calculate the unadjusted effect estimates and 95% confidence intervals. Two reviewers independently extracted important data fields, particularly outcome data, with discrepancies resolved through a combination of discussion and arbitration. All other fields were extracted by one person and verified by another person.

Quality assessment was performed using the Cochrane Risk of Bias in Non-randomized studies—of Exposures (ROBINS-E) tool.^17^ Two authors performed the assessment independently, and discrepancies were resolved through discussion to achieve consensus for domain-level and overall risk of bias judgements (see Supplementary material online, *Methods S4*).

### 2.6 Outcome synthesis and statistical analysis

We analyzed AMI and stroke as separate outcomes. Data were grouped and analyzed by viral pathogen and by the measure of effect reported (e.g. studies reporting OR were compared with other studies using OR). Where necessary, effect estimates were computed from cross-tabulated data. Clinical heterogeneity was assessed for each virus–outcome relationship by examining the population and outcome definition.

Study quality was a key consideration in the quantitative synthesis. We excluded individual studies that were classified as having a very high risk of bias and studies using self-reported outcome diagnoses. If there were ≥ 3 sufficiently homogenous studies, we meta-analyzed study-specific effect estimates. For SCCS studies with several estimates at different timepoints, within-study estimates were combined into an aggregated effect estimate using the inverse variance pooling method.^18^ Between-study heterogeneity was assessed using the Cochran *Q* test and Higgins *I^2^* statistic.^19^ Due to differences between exposure definitions and study designs, we pooled estimates using random-effects models (Hartung–Knapp adjustment for small sample sizes).^20^ For exposure–outcome pairs with <3 studies or due to statistical heterogeneity, effect estimate data were displayed in forest plots (i.e. without the summary diamond), including 95% confidence intervals, for visual comparison across studies. Where meta-analysis was inappropriate, the results were synthesized qualitatively.

We investigated sources of heterogeneity (from meta-analysis results) using subgroup analysis and assessed the impact of risk of bias and diagnostic method on the overall results in a sensitivity analysis. Due to inconsistencies in study design and effect estimate type within each exposure–outcome relationship, meta-regression was considered inappropriate and was not conducted. Where reporting bias was suspected, publication bias was assessed by visual inspection of funnel plot asymmetry. All analyses were performed using R Statistical Software version 4.3.2 (R Core Team).

### 2.7 Certainty assessment

The Grading of Recommendations, Assessment, Development and Evaluation (GRADE) approach was used to assess the certainty of the evidence for each respiratory virus on each outcome, across the five domains for population-level outcomes.^21^ The strength of evidence was categorized as very low, low, moderate, or high. Owing to the life-threatening, critical nature of our primary outcomes, we considered consistent non-zero relative effects (for example, RR > 1) and precise estimates to be important.

This review adheres to the Meta-analysis of Observational Studies in Epidemiology (MOOSE) and Preferred Reporting Items for Systematic Reviews and Meta-Analyses (PRISMA) guidelines (see Supplementary material online, *Methods S5andS6*).^22,23^

## 3. Results

### 3.1 Search results

**Figure 1:**
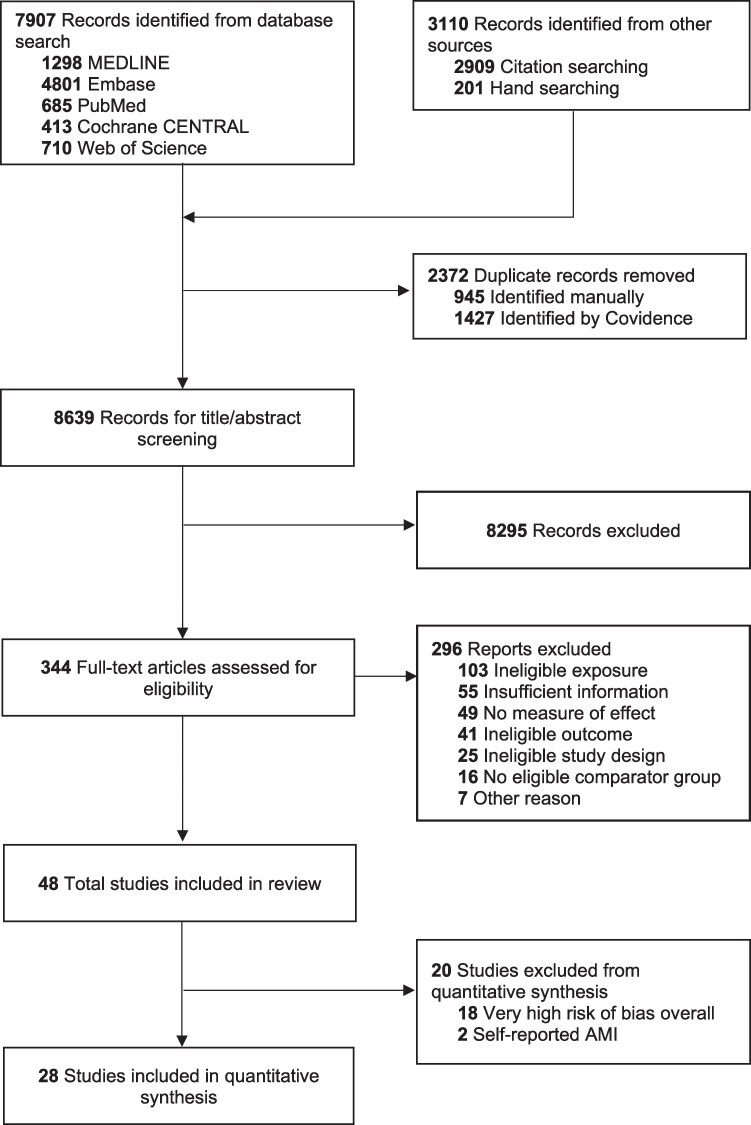
PRISMA flow diagram. Of 344 full-text articles assessed for eligibility, 54 reports of studies that met the inclusion criteria; multiple reports from the same study were merged, leaving 48 unique records. Of these, 18 studies at very high overall risk of bias and two studies using self-reported acute myocardial infarction (AMI) diagnosis were excluded from quantitative synthesis; thus 28 studies were selected for quantitative synthesis.

We identified a total of 11 017 records: 7907 records from database searches, and hand and citation searches yielding 3110 records (*Figure 1*). After duplicates were removed, we screened 8639 records at the title/abstract level and 8295 did not fulfil eligibility criteria. We reviewed 344 full-text articles. A total of 48 studies, published between 1978 and 2024, were included in the review.

### 3.2 Characteristics of included studies

The key characteristics of the included studies are summarized in *Table 1*. Of the 48 studies, there were 16 case-control studies, 18 cohort studies, and 14 SCCS designs—there were no randomized controlled trials. Among the included studies, 26 (54%) were conducted in Europe^26,27,38,40,49,51,54,58,59,62,69,71,72^ or UK^28,39,41,42,46,56,57,66,67,73,75,76^ (see Supplementary material online, *Figure S1*). Forty-two studies (85%) were conducted in hospital settings and two were autopsy studies with respiratory infection data collected postmortem.^26,63^ Total sample sizes ranged considerably depending on study design; case-control studies included between 50^44^ and 1470 patients.^57^ Self-controlled case series were small to moderate in size, with the exception of one US study of Medicare beneficiaries (*n* = 37 379).^60^ In contrast, cohort studies were considerably larger, for example, Schade Skov et al^58^ derived 4057 patients with ischaemic stroke from a population-based cohort of 3 423 877 participants. While the studies encompassed all ages, most were middle-aged to older patients, except for three paediatric stroke studies.^44,48,50^

**Table 1:** Characteristics of included studies by primary outcome (*n* = 48)^a^

First author, year, citation	Country	Study design^b^	Population	Age	Exposure	Outcome	Outcome definition	Risk period^c^	N^d^
**AMI**									
Akhtar, 2021^24^	Bangladesh	Cohort (p)	AMI admitted patients without suspected COVID-19	Adults ≥ 18 years	Influenza, SARS-CoV-2	Recurrent AMI	Self-report	3 months after index AMI	280
Aleem, 2023^25^	Bangladesh	Case-control	AMI admitted patients and healthy controls	Adults ≥ 40 years	Influenza, RSV, Adenovirus, HMPV	AMI	Clinical diagnosis	Samples within 72 h of the onset of AMI	240
Andreoletti, 2007^26^	France	Case-control	Patients who died suddenly	All ages 14–92 years	Enteroviruses	AMI death	Autopsy	Postmortem within 24 h	100
de Boer, 2024^27^	Netherlands	SCCS	Individuals tested for respiratory viral infection	Adults ≥ 35 years	Influenza, RSV	AMI	ICD coded	Up to 28 days	401^e^
Griffiths, 1980^28,29^	UK	Case-control	AMI admitted patients and outpatient controls	Adults 31–81 years	Coxsackie B	AMI	Clinical diagnosis	Samples in-hospital and at discharge	204
Guan, 2008^30,31^	China	Case-control	First-time AMI admitted patients and outpatient controls	Middle-aged adults	Influenza, RSV, Adenovirus	First-time AMI	Clinical diagnosis	Unclear	252
Kwong, 2018^32^	Canada	SCCS	Public health insurance claimants	Adults ≥35 years	Influenza, RSV	AMI	ICD coded	Up to 28 days	364^e^
Lau, 1986^33,34^	New Zealand	Case-control	AMI admitted patients and blood donor controls	Adults ≥20 years	Coxsackie B	AMI	Clinical diagnosis	Samples in-hospital	331
MacIntyre, 2011^35–37^	Australia	Case-control	AMI admitted patients and outpatient controls	Adults ≥40 years	Influenza	AMI	Clinical diagnosis	Blood samples at baseline and 4–6 weeks	559
Nikoskelainen, 1983^38^	Finland	Case-control	AMI admitted patients and hospital controls	Adults 16–87 years	Coxsackie B	AMI	Clinical diagnosis	Serum samples on admission and day of discharge (10–14 days later)	97
O’Neill, 1983^39^	UK	Case-control	Patients admitted with chest pain and outpatient controls	Middle-aged adults	Coxsackie B	AMI	Clinical diagnosis	Blood sample within 24 h of admission	230
Pönkä, 1981^40^	Finland	Case-control	AMI admitted patients and non-cardiac hospital controls	Adults 33–89 years	Influenza, Coxsackie B	AMI	Clinical diagnosis	Serum samples on admission and 2 weeks after	86
Warren-Gash, 2013^41^	UK	Case-control	AMI admitted patients and hospital controls	Adults ≥40 years	Influenza	AMI	Clinical diagnosis	Samples during current admission	134
Wood, 1978^42^	Scotland	Case-control	AMI admitted patients and outpatient controls with chest pain	Adults ≥30 years	Coxsackie B	AMI	Clinical diagnosis	Blood samples in-hospital and 10–14 days later	104
Young-Xu, 2020^43^	US	SCCS	Senior VHA-enrolled veterans	Adults ≥65 years	Influenza	AMI	ICD coded	7 days	391^e^
**Stroke**									
Abdelkreem, 2023^44^	Egypt	Case-control	Out-of-hospital AIS patients who were previously healthy and hospital controls	Children	SARS-CoV-2	Ischaemic stroke	Clinical diagnosis	Unclear	50
Belani, 2020^45^	US	Case-control	Acute ischaemic stroke patients matched to patients without stroke	Older adults	SARS-CoV-2	Ischaemic stroke	Clinical diagnosis	In-hospital approx. 2.5 weeks	123
Davidson, 2023^46^	UK	SCCS	First acute CVD event patients	Adults 40–84 years	SARS-CoV-2	Ischaemic stroke	ICD coded	Up to 91 days	401^e^
DeVries, 2023^47^	US	Cohort (r)	Medical insurance claimants for post-COVID-19	Middle-aged adults	SARS-CoV-2	Ischaemic stroke	ICD coded	Hospitalized cohort	11 091
dos Santos Brito Silva Furtado, 2016^48^	Brazil	Cohort (r)	Sickle cell disease patients	Children	Parvovirus B19	Stroke	Retrospective chart review	6 months before or 3 months after sample collection	229
Fuentes, 2021^49^	Spain	Cohort (r)	Acute stroke admitted to Madrid Stroke Units	Adults 63–82 years	SARS-CoV-2	Stroke recurrence	Clinical diagnosis	Unclear	550
Fullerton, 2017^50^	US	Case-control	Multicentre paediatric stroke study patients	Children	Adenovirus, Rhinovirus, HHV-6, Parvovirus B19	Ischaemic Stroke	Unclear	Blood samples within 3 weeks of stroke	195
Hautecloque, 2021^51^	France	Cohort (r)	Patients with ischaemic stroke or TIA	Adults 43–92 years	SARS-CoV-2	Ischaemic Stroke	Clinical diagnosis	Within 48 h of admission	6841
Hong, 2022^52^	South Korea	Cohort (r)	Individuals without history of CVD diseases	Undefined	SARS-CoV-2	First-time stroke	ICD coded	< 6 months	230 327
Huang, 2012^53^	China	Case-control	Plasma samples from stroke patients and controls	Older adults	CMV	Stroke	CT or MRI	Unclear	400
Lang, 2021^54^	Germany	Cohort (r)	Adult patients admitted with ARDS	Adults ≥18 years	SARS-CoV-2	Intra-cerebral haemorrhage	Clinical diagnosis	Approx. 3 months	163
Libruder, 2023^55^	Israel	SCCS	Adults without with previous strokes	Adults ≥18 years	SARS-CoV-2	First-time ischaemic stroke	ICD coded	Up to 28 days	555^e^
Patone, 2021^56^	UK	SCCS	Vaccinated individuals	Adults ≥16 years	SARS-CoV-2	Haemorrhagic stroke	ICD coded	Up to 28 days	462^e^
Perry, 2021^57^	UK	Case-control	All admitted stroke patients, excluding subarachnoid haemorrhage	Adults 61–84 years	SARS-CoV-2	Recurrent stroke	Clinical diagnosis	Recurrent stroke within 21 days of the index stroke	1470
Schade Skov, 2024^58^	Denmark	Cohort (r)	All Danish individuals who tested for SARS-CoV-2 during study period	Adults ≥18 years	SARS-CoV-2	Ischaemic stroke	ICD coded	Up to 194 days	3 423 877
Strambo, 2022^59^	Switzerland	Cohort (p)	Consecutive patients admitted with ischaemic stroke	Adults ≥18 years	SARS-CoV-2	Recurrent stroke	Clinical diagnosis	3 months	2338
Yang, 2022^60^	US	SCCS	US Medicare Fee-for-Service Beneficiaries	Adults ≥65 years	SARS-CoV-2	Ischaemic stroke	ICD coded	Up to 28 days	37 379e
Yurtsever, 2023^61^	Turkey	Cohort (r)	Patients presenting with TIA at ED	Adults 32–90 years	SARS-CoV-2	Stroke	Clinical diagnosis	Within 90 days	204
Zarifkar, 2022^62^	Denmark	Cohort (r)	Individuals tested in hospital-based facilities	Older adults	SARS-CoV-2	Ischaemic stroke	ICD coded	3 months	919 731
**AMI and stroke**									
Asumanu, 2022^63^	Ghana	Cohort (r)	Patients who died	All ages	SARS-CoV-2	AMI deaths; Stroke deaths	Autopsy	Postmortem	161
Azzalini, 2022^64^	US	Cohort (r)	Patients undergoing percutaneous coronary intervention	Older adults	SARS-CoV-2	AMI; Stroke	BMC2 registry	In-hospital	10 028
Coy-Cangucu, 2021^65^	Brazil	Cohort (r)	Patients admitted with acute respiratory distress syndrome	Adults ≥18 years	SARS-CoV-2	AMI; Ischaemic stroke	Clinical diagnosis	In-hospital	352
Hippisley-Cox, 2021^66^	UK	SCCS	Vaccinated individuals	Adults ≥16 years	SARS-CoV-2	AMI; Ischaemic stroke	ICD coded	Up to 28 days	AMI 6690; Stroke 3357^e^
Ho, 2021^67^	UK	SCCS	Individuals in Scotland who had confirmed COVID-19 and a thromboembolic event	Older adults	SARS-CoV-2	AMI; Ischaemic stroke	ICD coded	Up to 45 days	AMI 376; Stroke 560^e^
Korves, 2024^68^	US	SCCS	VHA-enrolled veterans	Older adults	Influenza	AMI; Ischaemic stroke	ICD coded	1–7 days	AMI 2148; Stroke 1212^e^
Koskinas, 2023^69^	Switzerland	Cohort (p)	Patients hospitalized in 5 Swiss cardiology centres	Older adults	SARS-CoV-2	AMI; Stroke	Retrospective chart review, self-report	In-hospital, 30 days	538
Ohland, 2020^70^	Denmark	SCCS	Patients admitted for first AMI and/or stroke	Adults ≥ 40 years	Influenza	First-time AMI; First-time Stroke	ICD coded	Up to 28 days	AMI 606; Stroke 744^e^
Ortega-Paz, 2022^71^	Spain/Italy	Cohort (r)	Consecutive patients who underwent a PCR	Adults ≥18 years	SARS-CoV-2	AMI; Ischaemic stroke	Clinical diagnosis	Acute phase (0–30 days)	4427
Profili, 2022^72^	Italy	Cohort (r)	Cases without prior AMI or stroke who tested positive for SARS-CoV-2 and matched controls	Adults 45–94 years	SARS-CoV-2	First-time AMI; First-time Stroke	ICD coded	6 months	83 110
Raisi-Estabragh, 2022^73^	UK	Cohort (p)	UK Biobank COVID-19 cases and propensity score-matched uninfected controls	Adults 40–69 years	SARS-CoV-2	AMI; Stroke	ICD coded	Average follow-up 141 days (range 32–395)	17 871
Rowe, 2023^74^	Australia	SCCS	COVID-19 cases notified to state government	All ages	SARS-CoV-2	AMI; Ischaemic stroke	ICD coded	Up to 89 days	AMI 94; Stroke 58^e^
Torabi, 2022^75^	UK	SCCS	Individuals with Welsh GP vaccine records	Adults aged ≥16 years	SARS-CoV-2	AMI; Ischaemic stroke	ICD coded	Up to 28 days	AMI 4102; Stroke 4215^e^
Warren-Gash, 2018^76,77^	UK	SCCS	Patients admitted for first AMI and stroke	Adults ≥40 years	Influenza	First-time AMI; First-time Stroke	ICD coded	Up to 28 days	AMI 1227; Stroke 762^e^

AIS, acute ischaemic stroke; ARDS, acute respiratory distress syndrome; BMC2: Blue Cross Blue Shield of Michigan Cardiovascular Consortium (BMC2) PCI registry; CMV, cytomegalovirus; CVD, cardiovascular disease; ED, emergency department; HHV-6, human herpesvirus 6; HMPV, human metapneumovirus; PCI, percutaneous coronary intervention; RSV, respiratory syncytial virus; SCCS, self-controlled case series; TIA, transient ischaemic attack; VHA, Veterans Health Affairs.

^a^Our definitions pertain to data derived from the study eligible for this review.

^b^Cohort study: prospective (p) or retrospective (r).

^c^Follow-up period for prospective studies, risk window for other study designs. For SCCS studies, the risk window refers to the days following infection compared with the baseline period.

^d^Total analyzed sample size eligible for inclusion in this review, including controls for cohort and case-control designs (including subjects without exposure or outcome).

^e^For SCCS ‘case-only’ study designs, total analyzed study population eligible for inclusion in this review.

Reported comorbidities were variable; most study populations had common cardiovascular risk factors at baseline, while a few studies explicitly focused on first-time cardiovascular events.^30,46,70,76^ Two studies were entirely comprised of vaccinated individuals.^56,66^

### 3.3 Risk of bias in included studies

**Figure 2:**
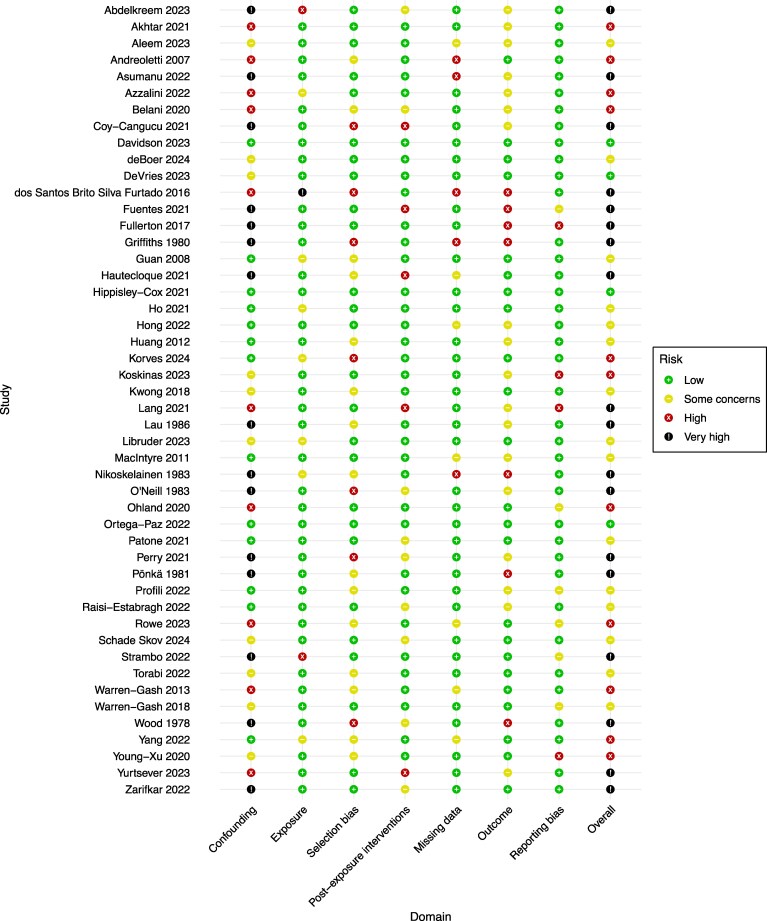
Risk of bias of included studies based on ROBINS-E domains.

Of the 48 included studies, risk of bias was relatively low across most ROBINS-E domains, including exposure, selection bias, postexposure intervention, missing data, outcome, and reporting bias. However, concerns in the confounding domain yielded higher overall judgements: 18 (38%) were rated very high risk, 11 (23%) were rated as high risk, 15 (31%) had some concerns, and only four (8%) studies were at low risk of bias (*Figure 2*).

### 3.4 Clinical outcomes

In terms of outcomes, 15 of the 48 studies reported on AMI, 19 studies on stroke, and 14 studies reported data on both AMI and stroke. The majority of studies defined AMI by either ICD-10 codes^27,32,43,66–68,70,72–76^ or physician diagnosis following standardized criteria.^25,28,30,33,35,38–42,65^ Three studies (two SCCS and one case-control study) focused specifically on first-time AMI.^30,70,76^ Two studies specifically on AMI deaths.^26,63^ Two studies included self-reported methods in conjunction with clinical data validation: Koskinas et al^62^ followed up cardiac patients post discharge for any new acute CVD events and corroborated with hospital discharge data, while Akhtar et al^24^ followed up previously admitted AMI patients for recurrent AMI events validated by physician’s notes.

Stroke diagnoses were consistent in terms of CT and/or MRI angiographic confirmation with clinical presentation, while outcomes reported were delineated by stroke subtypes and recurrent events among patients with prior stroke. Of these, three studies focused specifically on recurrent stroke soon after an index event.^49,57,59^ Over half of the studies defined stroke by ICD-9 or ICD-10 codes (17 studies),^46,47,52,55,56,58,60,62,66–68,70,72–76^ while 11 studies described clinical diagnosis confirmed by CT and/or MRI (10 studies).^44,45,49,51,53,54,57,59,61,65,71^ In terms of subtype, most studies reported on ischaemic stroke (21 studies), with fewer for haemorrhagic stroke (7 studies) and stroke deaths (3 studies).

### 3.5 Studies excluded from quantitative synthesis

As the overall risk of bias judgements were generally high due to poor control for confounding, 18 of 48 studies classified as having very high risk of bias^28,33,38–40,42,44,48–51,54,57,59,61–63,65^ were deemed inappropriate for meta-analysis. Two further studies by Koskinas et al^69^ and Akhtar et al^24^ were also excluded based on reliance on self-reported outcome ascertainment for AMI.

### 3.6 Respiratory viruses and AMI

**Figure 3:**
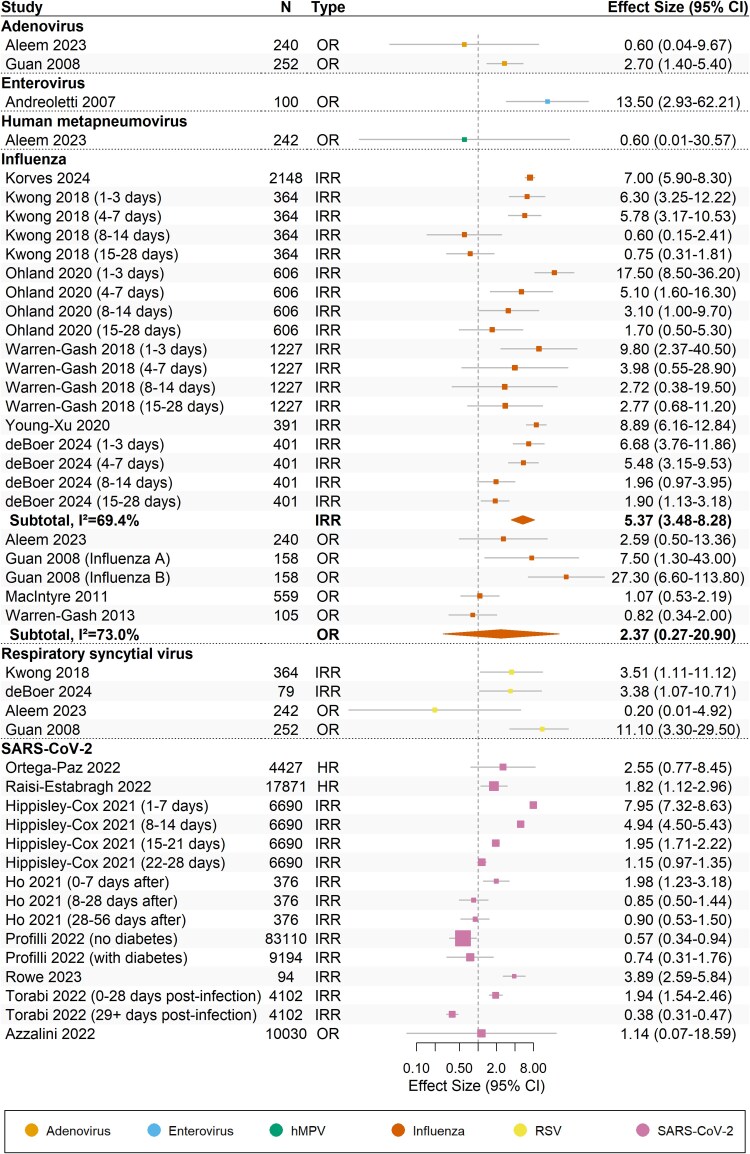
Forest plot for the effect of respiratory viral infection triggers on acute myocardial infarction (*n* = 19 studies). The dotted vertical line represents the line of no effect (effect size = 1). Boxes indicate reported estimates with whiskers representing 95% confidence intervals. Diamonds indicate pooled estimates from meta-analysis. HR, hazard ratio; IRR, incidence rate ratio; OR, odds ratio.

Twenty-nine studies reported on AMI and association with six different respiratory viruses: adenovirus, enteroviruses, human metapneumovirus (hMPV), influenza, respiratory syncytial virus (RSV), and SARS-CoV-2. Among these, estimates from 19 studies were suitable for quantitative synthesis (*Figure 3*).

Meta-analysis was conducted for the relationship between AMI with influenza. There was moderate-certainty of evidence for the association between influenza and AMI, based on estimates from 11 studies (*Table 2*).^25,27,30,32,35,40,41,43,68,70,76^ All studies had at least some concerns of risk of bias due to confounding. In a pooled analysis of six SCCS studies, influenza was associated with an increased risk of AMI within 28 days following a positive test (IRR, 5.37; 95% CI, 3.48–8.28; *I^2^* = 69.4%). However, pooled analysis of the remaining four case-control studies did not support an association with AMI (OR, 2.37; 95% CI, 0.27–20.90; *I^2^* = 73.0%). There was no evidence of publication bias (see Supplementary material online, *Figure S2*).

**Table 2:** Certainty of the evidence for a triggering association between respiratory viruses with acute myocardial infarction and stroke across GRADE domains

No. of studies	Virus	Risk of bias^a^	Inconsistency^b^	Indirectness^c^	Imprecision^d^	Publication bias	Other^e^	Overall Certainty
**Acute myocardial infarction**								
2	Adenovirus	Not serious	Not serious	Not serious	Serious	Undetected	-	⊕◯◯◯ Very Low
7	Enterovirus	Very serious	Not serious	Not serious	Serious	Undetected	Strong association (+2)	⊕⊕◯◯ Low
1	hMPV	Not serious	Not serious	Not serious	Very serious	Undetected	-	⊕◯◯◯ Very Low
11	Influenza	Not serious	Serious	Not serious	Serious	Undetected	Dose response (+1) Strong association (+2)	⊕⊕⊕◯ Moderate
4	RSV	Not serious	Not serious	Not serious	Serious	Undetected	Strong association (+1)	⊕⊕◯◯ Low
12	SARS-CoV-2	Not serious	Serious	Not serious	Serious	Undetected	Dose response (+2)	⊕⊕◯◯ Low
**Stroke**								
1	Adenovirus	Very serious	Not serious	Not serious	Very serious	Undetected	-	⊕◯◯◯ Very Low
1	CMV	Not serious	Not serious	Not serious	Not serious	Undetected	Strong association (+1)	⊕⊕⊕◯ Moderate
1	Rhinovirus	Very serious	Not serious	Not serious	Very serious	Undetected	-	⊕◯◯◯ Very Low
1	HHV-6	Very serious	Not serious	Not serious	Very serious	Undetected	-	⊕◯◯◯ Very Low
3	Influenza	Not serious	Not serious	Not serious	Serious	Undetected	Strong association (+2) No plausible confounding (+1)	⊕⊕⊕⊕ High
2	Parvovirus	Very serious	Serious	Serious	Serious	Undetected	-	⊕◯◯◯ Very Low
27	SARS-CoV-2	Serious	Serious	Not serious	Not serious	Undetected	Dose response (+2) Strong association (+1)	⊕⊕⊕◯ Moderate

CMV, cytomegalovirus; GRADE, Grading of Recommendations, Assessment, Development and Evaluation; HHV-6, human herpes virus-6; hMPV, human metapneumovirus; RSV, respiratory syncytial virus

^a^Risk of bias: *Not serious*—Studies had ≥1 domain at high risk of bias, predominantly only low risk to some concerns across most domains. *Serious*—Less than a third of studies had >2 domains at high or very high risk of bias, or less than a quarter of studies suffered from ≥2 domains at high risk of bias but these studies contributed <5% weight to the meta-analyses. *Very serious*—Study/studies had ≥3 domains at high or very high risk of bias.

^b^Inconsistency: *Not serious*—Where meta-analyses were possible, *I^2^* statistic did not show substantial heterogeneity, majority of point estimates across studies in the same direction. *Serious*—Where meta-analyses were possible, *I^2^* statistic indicated at least substantial heterogeneity, several point estimates across studies clearly in different directions.

^c^Imprecision: *Not serious*—Wide 95% CIs when event rates are low, however majority of estimates are precise and total sample sizes are large. *Serious*—wide 95% CIs around point estimates but total (cumulative) sample size is large. *Very serious*—wide 95% CIs around point estimates and total (cumulative) sample size is small (<300).

^d^Indirectness: *Serious*—Indirect study data (exposure 6 months before or 3 months after outcome).

^e^Other: *Dose–response*—clear dose response pattern with estimates from SCCS studies from relative incidence ratios over different timepoints post-infection.

A clear triggering association could not be identified between SARS-CoV-2 and AMI, based on 12 studies reporting on the association.^24,63–67,69,71–75^ It was not possible to meta-analyze the results of five SCCS studies reporting the association between SARS-CoV-2 and AMI due to considerable heterogeneity observed (*I^2^* = 98.6%).^66,67,72,74,75^ The risk of bias across studies were variable; 10 of 12 studies had at least some concerns in at least one domain. In a sensitivity analysis, variability by risk of bias did not substantially alter the meta-analysis results (see Supplementary material online, *Table S2*).

Enteroviruses, mainly Coxsackie B, were associated as a trigger for AMI in seven studies. In one study focused specifically on AMI deaths, enterovirus infection was associated with 13.5 times the odds of death following a 4-fold increase in titer from paired sera.^26^ Five (71%) of seven enterovirus studies suggested an increased risk of AMI following infection.^26,28,33,38–40,42^ However, the association was inconsistent, with two of the seven studies reporting an association in the opposite direction.^40,42^ Further, all these studies were assessed as having high to very high risk of bias, largely due to poor control for confounding, which precluded pooling these data in a meta-analysis.

Owing to the low number of sufficiently homogenous studies, pooled estimates were not generated for adenovirus, hMPV, and RSV. In three of the four identified studies, RSV was associated with increased risk of AMI.^27,30,32^ Two SCCS studies showed RSV triggering AMI, with incidence rate ratios as high as 11-fold within 7 days following positive specimen collection,^27,32^ and one case-control study by Guan et al^31^ also reporting higher odds of AMI in 2010, although this differed from recent case-control study results by Aleem et al.^31^ Studies of RSV were at low risk of bias, but heterogeneity due to differing study designs precluded meta-analysis of the effect estimates. In terms of other respiratory viruses, Guan et al^31^ and Aleem et al^25^ showed there was no evidence that adenovirus or hMPV was associated with AMI.

### 3.7 Respiratory viruses and stroke

**Figure 4:**
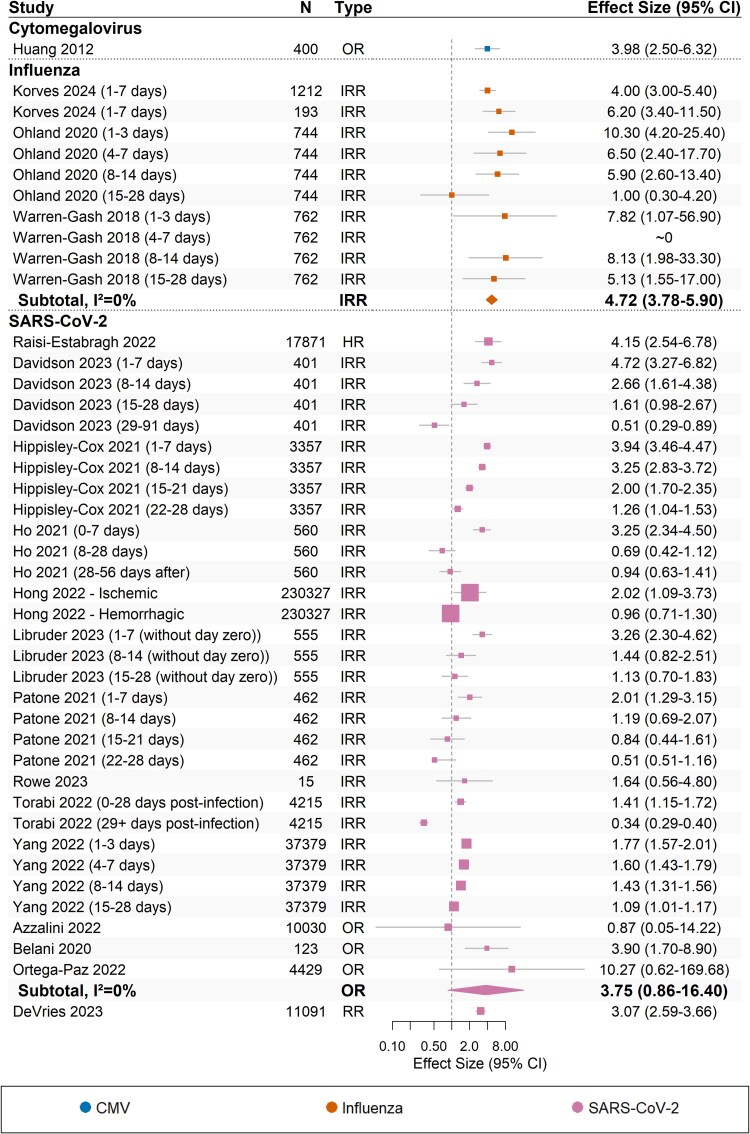
Forest plot for the effect of respiratory viral infection triggers on stroke (*n* = 18 studies). The dotted vertical line represents the line of no effect (effect size = 1). Boxes indicate reported estimates with whiskers representing 95% confidence intervals. Diamonds indicate pooled estimates from meta-analysis. HR, hazard ratio; IRR, incidence rate ratio; OR, odds ratio; RR, relative risk.

A total of 33 studies reported associations between stroke and seven different respiratory viruses: adenovirus, cytomegalovirus (CMV), human herpes virus 6 (HHV-6), influenza, parvovirus B19, rhinovirus, and SARS-CoV-2. Data from 18 studies that reported on CMV, influenza, and SARS-CoV-2, were suitable for quantitative synthesis (*Figure 4*). Meta-analysis was performed for the relationship between influenza and SARS-CoV-2 with stroke.

Influenza was associated with an increased risk of stroke (IRR, 4.72; 95% CI, 3.78–5.90; *I^2^* = 0%), based on 10 estimates from three heterogenous SCCS studies.^68,70,76^ Among them, there was generally low risk of bias across most domains, albeit at least some concerns across several domains. However, the strength and consistency of the association and the relatively narrow confidence intervals around the pooled estimate culminated in high certainty of evidence (*Table 2*).

SARS-CoV-2 was frequently described with stroke. On pooled analysis of three estimates from three similarly designed studies, SARS-CoV-2 was not significantly associated with stroke (OR, 3.75; 95% CI, 0.86–16.40; *I^2^* = 0%), despite the strong association from contributing estimates. Nine SCCS studies demonstrated a clear dose-response relationship with the highest risk in the days immediately following infection (*Figure 4*), although data from these studies were not pooled in a meta-analysis due to statistical heterogeneity (*I^2^* = 96.9%). Because of the inconsistency of association and risk of bias from contributing studies, the certainty of the evidence for a triggering association between SARS-CoV-2 and stroke was moderate. Subgroup analyses by specific subtype yielded largely similar results for the association between SARS-CoV-2 and ischaemic and haemorrhagic strokes (see Supplementary material online, *Figures S3*–S5).

There was limited evidence regarding other pathogens, namely adenovirus, CMV, HHV-6, parvovirus B19, and rhinovirus. One case-control study showed that CMV was associated with significantly higher risk of stroke (OR, 3.98; 95% CI, 2.50–6.32), even after adjusting for age, sex, BMI, hypertension, and smoking, among an older adult population.^53^ In contrast, adenovirus, HHV-6, parvovirus B19, and rhinovirus were identified entirely from three studies of paediatric stroke (see Supplementary material online, *Figure S6*). Two studies assessed parvovirus infection and ischaemic stroke: Brazilian case-control study showed no association in a population of children with sickle cell disease (OR, 0.52; 95% CI, 0.03–9.63),^48^ comparable to results from Fullerton et al^50^ in their paediatric study (OR, 4.78; 95% CI, 0.27–83.59). Fullerton et al^50^ also found no observed increase in ischaemic stroke following adenovirus, rhinovirus, or HHV-6 infection from multiplex PCR, based on a 3-week risk window.^50^

## 4. Discussion

Myocardial infarction and stroke remain the world’s two leading causes of premature death.^3^ Given the critical, life-threatening nature of these events, knowledge of viral triggers to inform policies that may reduce AMI and stroke represents a global health priority. Over the last two decades, laboratory techniques, such as multiplex PCR panels, have substantially improved the ascertainment of respiratory viral infections.^78^ Over this period, infections involving the respiratory tract have been most frequently implicated as cardiovascular triggers.^7^ However, limited research has been conducted beyond influenza to investigate the relative contributions of respiratory viruses to acute cardiovascular outcomes. We present a comprehensive systematic review including meta-analysis of pathogen-specific associations with AMI and stroke, incorporating comprehensive database coverage and a highly inclusive search spanning over 40 years of literature. This study adds new knowledge by providing a comprehensive picture of the role of multiple respiratory viruses and the pathogens most strongly associated with each outcome. Our specific focus on laboratory-confirmed infection was important to differentiate between infections for which vaccines or antiviral preventions are available. We found that common respiratory viruses play a role in precipitating acute cardiovascular events, but the strength of association and certainty of evidence varies across attributable pathogens.

The key pathogens of interest were SARS-CoV-2 and influenza. Our results are consistent with those of recent reviews^12,79^ and support ongoing vaccine trials assessing cardiovascular endpoints among high-risk patients, particularly the relationship between influenza and AMI. There was good evidence for the link between influenza with stroke—meta-analysis from SCCS studies of influenza showed a 4.7-fold increased risk of stroke within the first 28 days, comparable to a recent SCCS study of 2.2 million at-risk patients observing the same timepoints.^80^ Notably, there was a large body of data for SARS-CoV-2 for both AMI and stroke, indicative of the breadth of information and fast-tracked evidence that has become available since the COVID-19 pandemic. The biological mechanisms by which SARS-CoV-2 and influenza viruses may contribute to atherosclerosis are generally believed to be associated with inflammatory cascades evoked by many pathogens.^41^ Meanwhile, there was a paucity of evidence from RCTs, as influenza vaccine trials are currently ongoing, and insufficient time has elapsed for COVID-19 vaccines.^81^ The finding that laboratory-confirmed SARS-CoV-2 and influenza were the most frequently associated triggers suggests that they are important for improved risk prediction modelling in the short-term period following infection, particularly since confounding was the main issue affecting risk of bias among studies. Vaccine probe studies specific to influenza and SARS-CoV-2, whether randomized or observational, for the prevention of acute cardiovascular events may be helpful to assess the impact on population burden.

Enteroviruses, particularly Coxsackie B, may trigger AMI. This association was surprising given that the current paradigm has described enteroviruses as *cardiotropic,* causing inflammation and tissue damage to the myocardium,^82^ rather than as a contributor to atherosclerosis. It is reasonable to postulate inadequate control for confounding, especially with six of seven studies conducted in the 1970s to 1980s,^28,33,38–40,42^ but we cannot rule out the possibility of outcome misclassification with myocarditis. Severe localized myocarditis may have been clinically indistinguishable from AMI, and it is worth noting that modern cardiovascular biomarkers (e.g. troponin)^83^ have considerably improved sensitivity and specificity of diagnosis since these studies were conducted. Nevertheless, as serology to prove acute infections is often non-specific and enteroviruses are not generally recognized as important AMI triggers beyond isolated reports, further investigation with higher-quality data is needed.

RSV was linked to AMI in four studies, but the data were less convincing and of lower quality than those of other respiratory viruses. This potentially reflects low RSV testing rates (due to limited awareness of its role in adult respiratory disease) and underestimated incidence in many countries.^84^ Moreover, it may be difficult to differentiate RSV infection from other respiratory viruses due to overlapping seasonality and similar clinical presentations. However, improved laboratory testing has recently led to RSV being increasingly recognized as an important respiratory illness among cardiovascular disease patients.^85^ With recent developments in RSV vaccines, ongoing real-world observational analyses and postlicensure monitoring of cardiovascular events will be important to provide insights about any potential risk-benefit.

Moderate-certainty evidence indicated a link between CMV and stroke, although herpesviruses were generally not found to be common triggers for stroke. This is contrary to previous studies^9^ and is likely to be explained by the fact that testing for recent infection or reactivation of latent viruses is infrequently clinically indicated. Our findings may underestimate the role of herpesviruses, considering that laboratory testing is challenging without demonstrable seroconversion. The exposure definition may have reduced the sensitivity for capturing latent viruses, albeit specifically designed to observe a triggering effect.

This systematic review has some limitations. First, the results of this study cannot be extrapolated to all patients. The populations included in the review are likely to reflect middle-aged or older patients with (known or unknown) prior cardiovascular risk factors. Our systematic review was designed to be as inclusive as possible with respect to population characteristics, but this may have introduced heterogeneity, making it difficult to interpret between different cardiovascular risk profiles. It is understood that there is a triggering effect of respiratory infections on acute cardiovascular events that is short-term and transient in nature, but this is subject to debate among people without pre-existing disease,^86^ and the population of interest remains unclear. It was not possible to assess the viral triggers for different at-risk groups given the limited data on attributable viral triggers in relation to our specific endpoints and even less so for comorbidities. Second, although we included a broad range of respiratory viruses, there may be biases towards developed countries. While this was mitigated by employing a clearly defined, prespecified list, the selection process for viruses to include or exclude was challenging, and our findings may favour commonly tested respiratory pathogens in countries with resources for laboratory confirmatory testing. Third, the quantitative analysis was complex due to multiple effect estimates. We handled multiplicity by selecting the most precise point estimate eligible for inclusion and computing a summary effect over all timepoints for SCCS studies, in line with Cochrane guidelines.^16^ However, the insufficient number of studies in some exposure–outcome pairs and different effect types limited our ability to perform further subgroup analyses and meta-regression, as specified *a priori.* For example, heterogeneity was investigated by excluding studies with high or very high risk of bias, which often reduced the total sample size and thus precision of confidence intervals around the pooled estimate (see Supplementary material online, *Tables S2and S3*). Moreover, although we included a range of epidemiological study designs suitable for answering an aetiological question, this resulted in less consistent data compared with reviews limiting to one study type (e.g. case-control studies). Finally, our systematic review comprised entirely of observational studies, which are affected by inherent design-specific biases, such as confounding and lack of temporality. Although we aimed to include evidence from both randomized and observational studies, no RCTs met the inclusion criteria. Therefore, causal inference should be made cautiously.

## 5. Conclusions

Common, often vaccine-preventable, respiratory viruses play a role in precipitating acute cardiovascular events. The strength of association and certainty of evidence varies across different causal pathogens, with SARS-CoV-2 and influenza being the most strongly associated triggers. Vaccine probe studies may be helpful in assessing the impact of influenza and SARS-CoV-2 on population burden. There was an association with enteroviruses and inconsistent evidence for RSV linked to AMI that warrant further investigation. The findings of this systematic review have implications for clinicians regarding transient, acute cardiovascular risk following respiratory viral infection, particularly among high-risk patient groups.

## Supplementary Material

cvaf092_Supplementary_Data

## Data Availability

The data underlying this article were derived from sources in the public domain. Where not already provided as part of this manuscript or its supporting information, other relevant study data (e.g. quality assessment checklists, R code) are available from the corresponding author upon reasonable request.
