# Supplementary material for: Systematic review and meta-analysis of respiratory viral triggers for acute myocardial infarction and stroke
Source: Cardiovasc Res. 2025 Jun 17;121(9):1330–44. doi: 10.1093/cvr/cvaf092 (PMC12352309; doi:10.1093/cvr/cvaf092)
Supplement: cvaf092_Supplementary_Data [file cvaf092_supplementary_data.docx]

**SUPPLEMENTARY FIGURES/TABLES**

**Supplementary Table 1.** Characteristics of Excluded studies

**Supplementary Table 2.** Sensitivity Analysis Exploring the Association between Overall Risk of Bias and Diagnostic Method on Meta-Analysis Results for Acute Myocardial Infarction

**Supplementary Table 3.** Sensitivity Analysis Exploring the Association between Overall Risk of Bias, Diagnostic Method and Children/Adults on Meta-Analysis for Stroke

**Supplementary Figure 1.** Geographical Distribution of Included Studies (n = 48)

**Supplementary Figure 2.** Funnel Plot for Meta‐Analysis of Influenza and Acute Myocardial Infarction

**Supplementary Figure 3.** Forest Plot of Ischemic Stroke and SARS-CoV-2

**Supplementary Figure 4.** Forest Plot of Hemorrhagic Stroke and SARS-CoV-2

**Supplementary Figure 5**. Forest Plot of Stroke Deaths and SARS-CoV-2

**Supplementary Figure 6.** Forest Plot of Stroke and Respiratory Viral Triggers Among Children

**SUPPLEMENTARY METHODS**

**Supplementary Methods 1.** Protocol Changes

**Supplementary Methods 2.** Databases Search Strategy Results

**Supplementary Methods 3.** Inclusion/Exclusion Criteria List

**Supplementary Methods 4.** Risk of Bias Assessment – Approach to Signaling Questions

**Supplementary Methods 5.** Preferred Reporting Items for Systematic Reviews and Meta-analyses (PRISMA) Checklist

**Supplementary Methods 6.** Meta-Analysis of Observational Studies in Epidemiology (MOOSE) Checklist

**SUPPLEMENTARY REFERENCES**

**SUPPLEMENTARY FIGURES/TABLES**

**Supplementary Table 1.** Characteristics of Excluded Studies

| **Reason for exclusion** | **No. of studies (references)** |
| --- | --- |
| Ineligible exposure | 103^1–103^ |
| Insufficient information | 55^104–158^ |
| No measure of effect | 49^159–207^ |
| Ineligible outcome | 41^208–248^ |
| Ineligible study design | 25^249–273^ |
| No eligible comparator group | 16^274–289^ |
| Other reason | 7^290–296^ |
| **Total excluded** | **296** |

All records at full-text screening were independently reviewed by 2 people in Covidence. At full-text screening, if multiple exclusion reasons were applicable, the first (primary reason) for exclusion is cited.

Studies that potentially met eligibility criteria but were excluded: 7 studies excluded after failure to obtain a reply from authors to confirm against eligibility criteria; 3 studies excluded after successful contact with authors confirm against eligibility criteria; 2 studies excluded after failure to obtain a reply from authors for additional data; and 3 studies excluded after successful contact with authors for additional data.

**Supplementary Table 2.** Sensitivity Analysis Exploring the Association between Overall Risk of Bias and Diagnostic Method on Meta-Analysis Results for Acute Myocardial Infarction

|  | **Effect type** | **No. of contributing studies from 29** | **I^2^ (%)** | **τ²** | **Cochrane’s Q test p-value^b^** | **Pooled ES (95% CI); p-value ^a^** |
| --- | --- | --- | --- | --- | --- | --- |
| **Adenovirus** |  |  |  |  |  |  |
| All studies | OR | 2 | - | - | - | - |
| **Enterovirus** |  |  |  |  |  |  |
| All studies | OR | 7 | 34.2 | 0.721 | 0.025 | 2.40 (0.86, 6.67); p=0.082 |
| Remove very high risk of bias | OR | 1 | - | - | - | - |
| Remove high-very high risk of bias | OR | 0 | - | - | - | - |
| Clinical diagnosis only | OR | 6 | 20.0 | 0.396 | 0.107 | 1.80 (0.73, 4.45); p=0.157 |
| **Human metapneumovirus** |  |  |  |  |  |  |
| All studies | OR | 1 | - | - | - | - |
| **Influenza** |  |  |  |  |  |  |
| All studies | IRR | 6 | 69.4 | 0.140 | <0.001 | 5.37 (3.48, 8.28); p<0.001 |
| Remove very high risk of bias | IRR | 6 | 69.4 | 0.140 | <0.001 | 5.37 (3.48, 8.28); p<0.001 |
| Remove high-very high risk of bias | IRR | 3 | 0.0 | 0.000 | 0.337 | 3.53 (2.83, 4.40); p<0.001 |
| Clinical diagnosis only | IRR | 0 | - | - | - | - |
| ICD coded only | IRR | 6 | 69.4 | 0.140 | <0.001 | 5.37 (3.48, 8.28); p<0.001 |
| All studies | OR | 5 | 67.7 | 1.502 | <0.001 | 1.82 (0.34, 9.84); p=0.379 |
| Remove very high risk of bias | OR | 4 | 73.0 | 1.612 | <0.001 | 2.37 (0.27, 20.90); p=0.297 |
| Remove high-very high risk of bias | OR | 3 | 77.3 | 1.796 | <0.001 | 3.48 (0.10, 120.17); p=0.269 |
| Clinical diagnosis only | OR | 5 | 67.7 | 1.502 | <0.001 | 1.82 (0.34, 9.84); p=0.379 |
| ICD coded only | OR | 0 | - | - | - | - |
| **Respiratory syncytial virus** |  |  |  |  |  |  |
| All studies | IRR | 2 | - | - | - | - |
| All studies | OR | 2 | - | - | - | - |
| **SARS-CoV-2** |  |  |  |  |  |  |
| All studies | HR | 3 | 0.0 | 0.000 | 0.877 | 1.91 (1.23, 2.98); p=0.004 |
| Remove very high risk of bias | HR | 3 | 0.0 | 0.000 | 0.877 | 1.91 (1.23, 2.98); p=0.004 |
| Remove high-very high risk of bias | HR | 2 | - | - | - | - |
| Clinical diagnosis only | HR | 1 | - | - | - | - |
| ICD coded only | HR | 1 | - | - | - | - |
| All studies | IRR | 5 | 98.6 | 0.817 | <0.001 | 1.59 (0.51, 4.95); p=0.324 |
| Remove very high risk of bias | IRR | 5 | 98.6 | 0.817 | <0.001 | 1.59 (0.51, 4.95); p=0.324 |
| Remove high-very high risk of bias | IRR | 4 | - | - | - | - |
| Clinical diagnosis only | IRR | 0 | - | - | - | - |
| ICD coded only | IRR | 5 | 98.6 | 0.817 | <0.001 | 1.59 (0.51, 4.95); p=0.324 |
| All studies | OR | 3 | 0.0 | 0.000 | 0.663 | 0.97 (0.09, 10.15); p=0.974 |
| Remove very high risk of bias | OR | 1 | - | - | - | - |
| Remove high-very high risk of bias | OR | 0 | - | - | - | - |
| Clinical diagnosis only | OR | 1 | - | - | - | - |
| ICD coded only | OR | 0 | - | - | - | - |
| All studies | RR | 1 | - | - | - | - |
| Remove very high risk of bias | RR | 1 | - | - | - | - |
| Remove high-very high risk of bias | RR | 0 | - | - | - | - |
| Clinical diagnosis only | RR | 0 | - | - | - | - |
| ICD coded only | RR | 0 | - | - | - | - |

Abbreviations: ES, Effect Size; HR, hazard ratio; IRR, incidence rate ratio; OR, odds ratio; RR, relative risk.

^a^ Sensitivity analysis was only performed for meta-analyzed data. Meta-analysis was only performed in the primary analysis (including all studies) if there were ≥3 studies with the same exposure and outcome. For exposure-outcome pairs with <3 studies, a dash (-) indicates no pooled result.

^b^ Cochrane’s Q test may less reliable for relatively low number of studies.

**Supplementary Table 3.** Sensitivity Analysis Exploring the Association between Overall Risk of Bias, Diagnostic Method and Children/Adults on Meta-Analysis for Stroke

|  | **Effect type** | **No. of contributing studies from 33** | **I^2^ (%)** | **τ²** | **Cochrane’s Q test p-value^b^** | **TE (95% CI, p-value)** |
| --- | --- | --- | --- | --- | --- | --- |
| **Adenovirus** |  |  |  |  |  |  |
| All studies | OR | 1 | - | - | - | - |
| **Cytomegalovirus** |  |  |  |  |  |  |
| All studies | OR | 1 | - | - | - | - |
| **Herpes virus-6** |  |  |  |  |  |  |
| All studies | OR | 1 | - | - | - | - |
| **Influenza** |  |  |  |  |  |  |
| All studies | IRR | 3 | 0.0 | 0.000 | 0.496 | 4.72 (3.78, 5.90); p<0.001 |
| Remove very high risk of bias | IRR | 3 | 0.0 | 0.000 | 0.496 | 4.72 (3.78, 5.90); p<0.001 |
| Remove high-very high risk of bias | 1RR | 1 | - | - | - | - |
| **Parvovirus B19** |  |  |  |  |  |  |
| All studies | OR | 2 | - | - | - | - |
| **Rhinovirus** |  |  |  |  |  |  |
| All studies | OR | 1 | - | - | - | - |
| **SARS-CoV-2** |  |  |  |  |  |  |
| All studies | HR | 3^b^ | - | - | - | ^-^ |
| Remove very high risk of bias | HR | 3^b^ | - | - | - | - |
| Remove high-very high risk of bias | HR | 2 | - | - | - | - |
| Clinical diagnosis only | HR | 0 | - | - | - | - |
| ICD coded only | HR | 2 | - | - | - | - |
| Remove children | HR | 3^c^ | - | - | - | ^-^ |
| All studies | IRR | 9 | 96.9 | 0.233 | <0.001 | 1.44 (0.99, 2.09); p=0.057 |
| Remove very high risk of bias | IRR | 9 | 96.9 | 0.233 | <0.001 | 1.44 (0.99, 2.09); p=0.057 |
| Remove high-very high risk of bias | IRR | 7 | 97.4 | 0.291 | <0.001 | 1.44 (0.87, 2.38); p=0.128 |
| Clinical diagnosis only | IRR | 0 | - | - | - | - |
| ICD coded only | IRR | 9 | 96.9 | 0.233 | <0.001 | 1.44 (0.99, 2.09); p=0.057 |
| Remove children | IRR | 9 | 96.9 | 0.233 | <0.001 | 1.44 (0.99, 2.09); p=0.057 |
| All studies | OR | 11 | 41.0 | 0.489 | 0.002 | 1.88 (1.19, 2.99); p=0.195 |
| Remove very high risk of bias | OR | 3 | 0.0 | 0.000 | 0.460 | 3.75 (0.86, 16.40); p=0.061 |
| Remove high-very high risk of bias | OR | 1 | - | - | - | - |
| Clinical diagnosis only | OR | 9 | 50.5 | 0.610 | <0.001 | 1.49 (0.72, 3.06); p=0.239 |
| ICD coded only | OR | 0 | - | - | - | - |
| Remove children | OR | 10 | 44.7 | 0.556 | <0.001 | 1.40 (0.73, 2.69); p=0.274 |
| All studies | RR | 2 | - | - | - | - |
| Remove very high risk of bias | RR | 1 | - | - | - | - |
| Remove high-very high risk of bias | RR | 1 | - | - | - | - |
| Clinical diagnosis only | RR | 0 | - | - | - | - |
| ICD coded only | RR | 2 | - | - | - | - |
| Remove children | RR | 2 | - | - | - | - |

Abbreviations: ES, Effect Size; HR, hazard ratio; IRR, incidence rate ratio; OR, odds ratio; RR, relative risk.

^a^ Sensitivity analysis was only performed for meta-analyzed data. Meta-analysis was only performed in the primary analysis (including all studies) if there were ≥3 studies with the same exposure and outcome. For exposure-outcome pairs with <3 studies, a dash (-) indicates no pooled result.

^b^ Cochrane’s Q test may less reliable for relatively low number of studies.

^c^ Data not pooled due to zero events in one study.

**Supplementary Figure 1.** Geographical Distribution of Included Studies (n = 48)


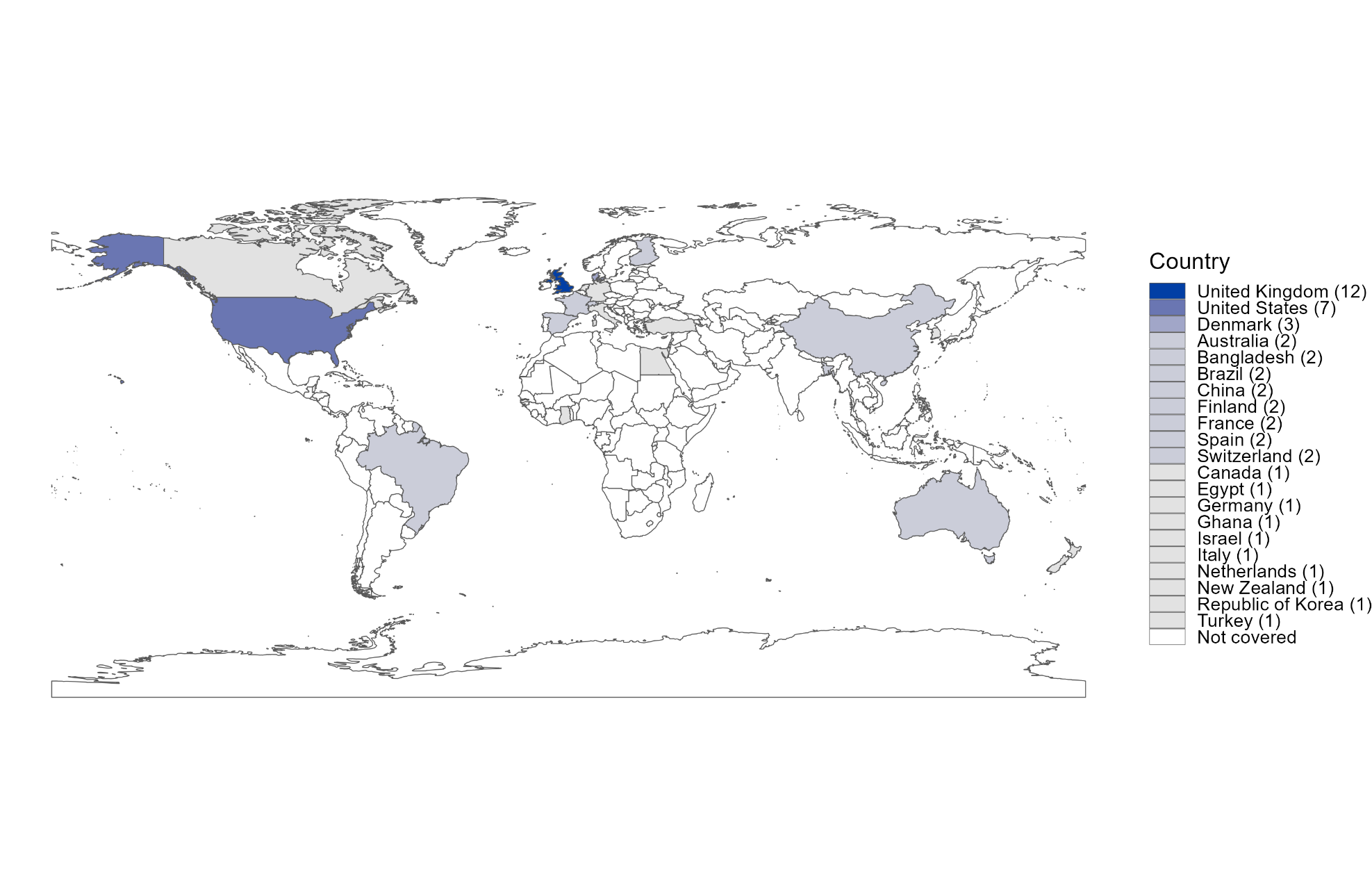


Among the 48 studies included in the systematic review, 12 were conducted in the United Kingdom,^17–28^ 14 across other countries in Europe,^29–41^ 8 in North America,^42–49^ 5 in Asia,^50–54^ 5 in Australasia,^55–57^ 2 in the Middle East,^58,59^ 2 in Brazil,^60,61^ 1 in Egypt^62^ and 1 in Ghana.^63^

**Supplementary Figure 2.** Funnel Plot for Meta‐Analysis of Influenza and AMI


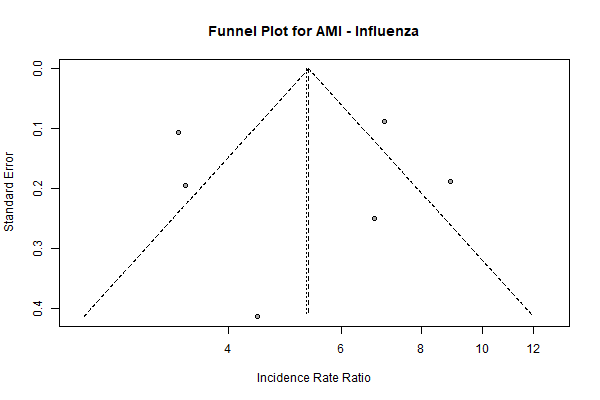


Upon examination of funnel plot asymmetry, there was insufficient evidence of publication bias among studies reporting incidence rate ratios (n = 6) for the association between influenza and AMI.

**Supplementary Figure 3.** Forest Plot of Ischemic Stroke and SARS-CoV-2


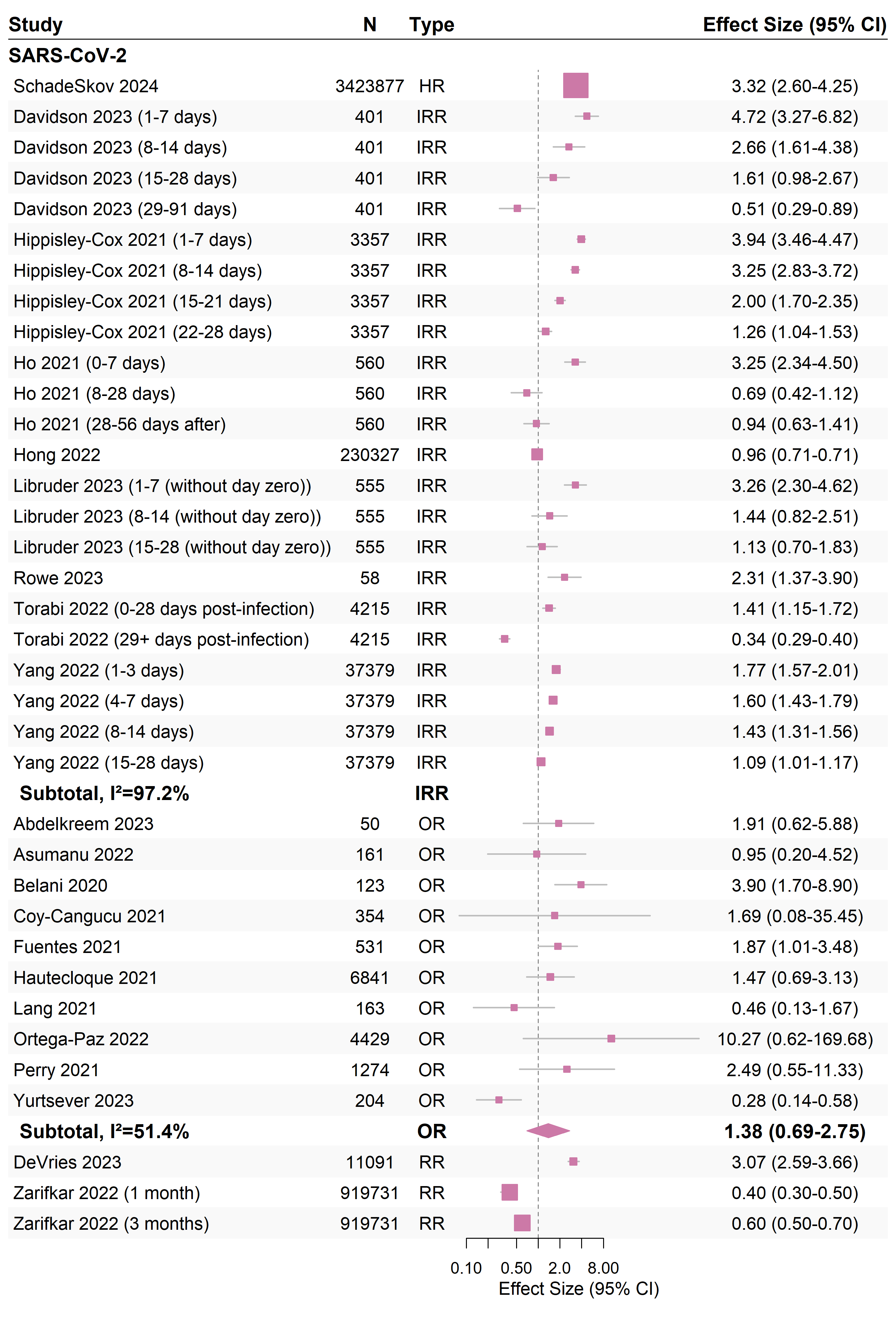


The dotted vertical line represents the line of no effect (effect size = 1). Boxes indicate reported estimates with whiskers representing 95% confidence intervals. Diamonds indicate pooled estimates from meta-analysis. HR, hazard ratio; IRR, incidence rate ratio; OR, odds ratio; RR, relative risk.

**Supplementary Figure 4.** Forest Plot of Hemorrhagic Stroke and SARS-CoV-2


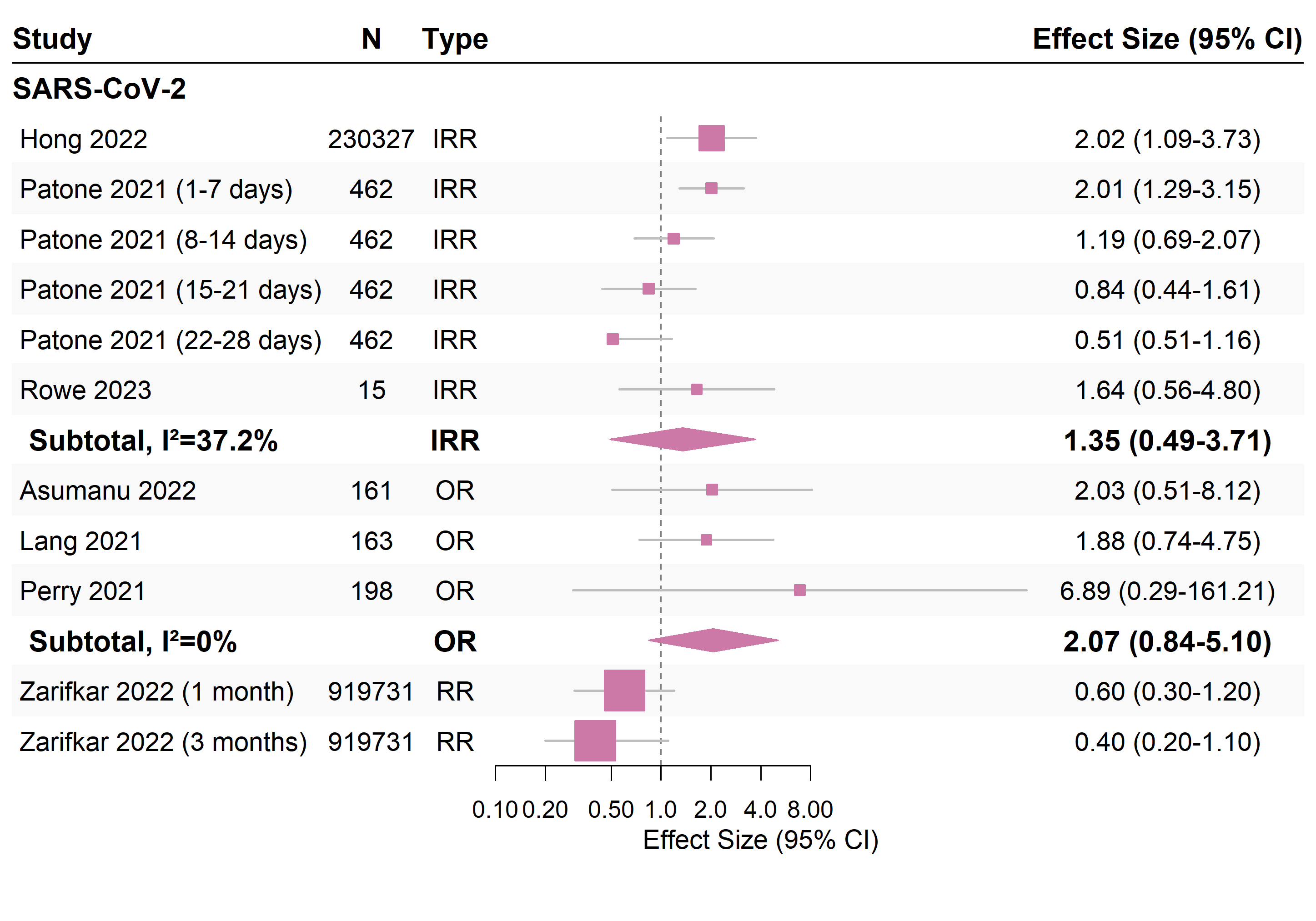


The dotted vertical line represents the line of no effect (effect size = 1). Boxes indicate reported estimates with whiskers representing 95% confidence intervals. Diamonds indicate pooled estimates from meta-analysis. IRR, incidence rate ratio; OR, odds ratio; RR, relative risk.

**Supplementary Figure 5.** Forest Plot of Stroke Deaths and SARS-CoV-2


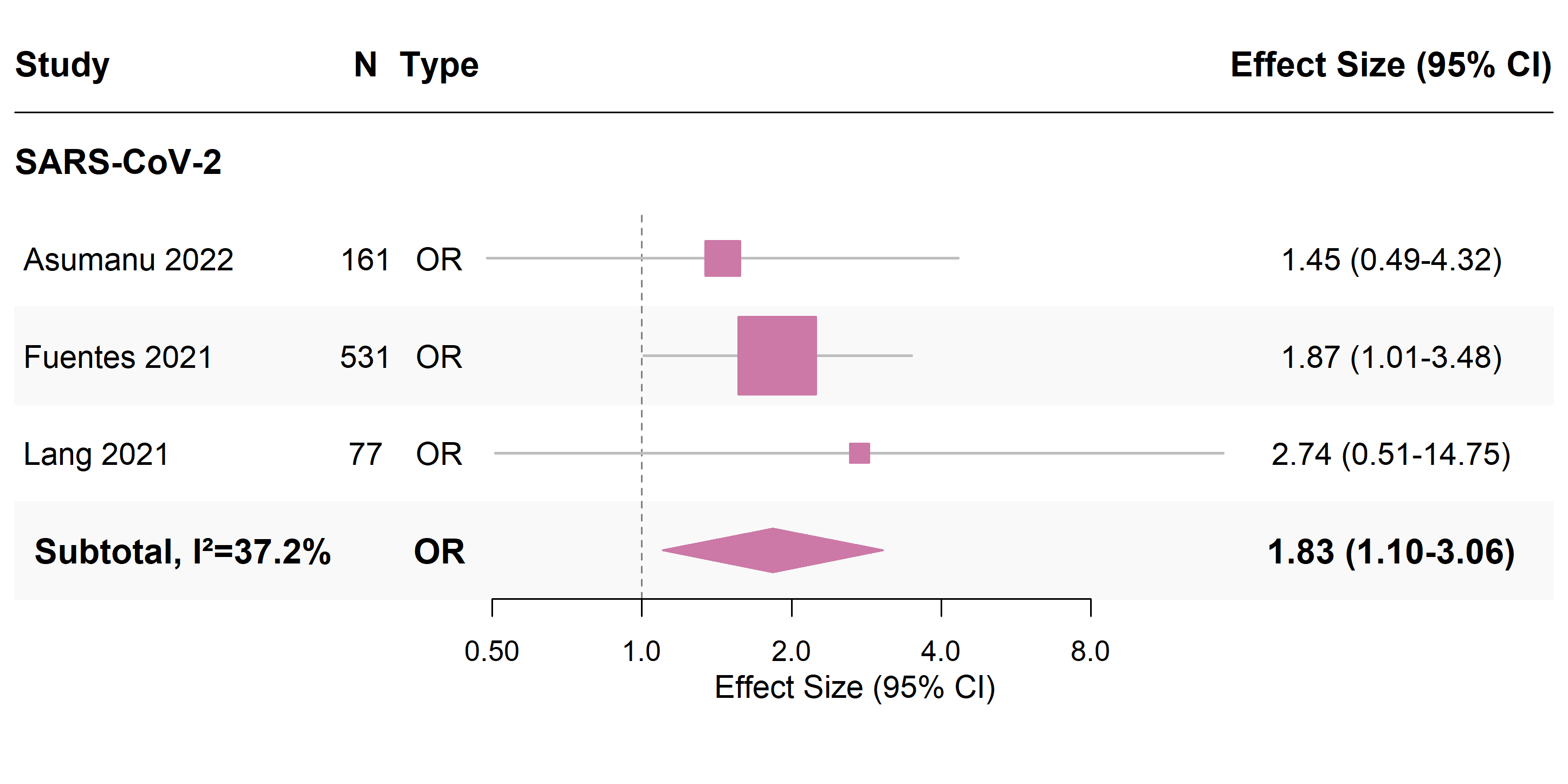


The dotted vertical line represents the line of no effect (effect size = 1). Boxes indicate reported estimates with whiskers representing 95% confidence intervals. The diamond indicates a pooled estimate from meta-analysis. OR, odds ratio.

**Supplementary Figure 6.** Forest Plot of Respiratory Viral Infection Triggers on Stroke Among Children


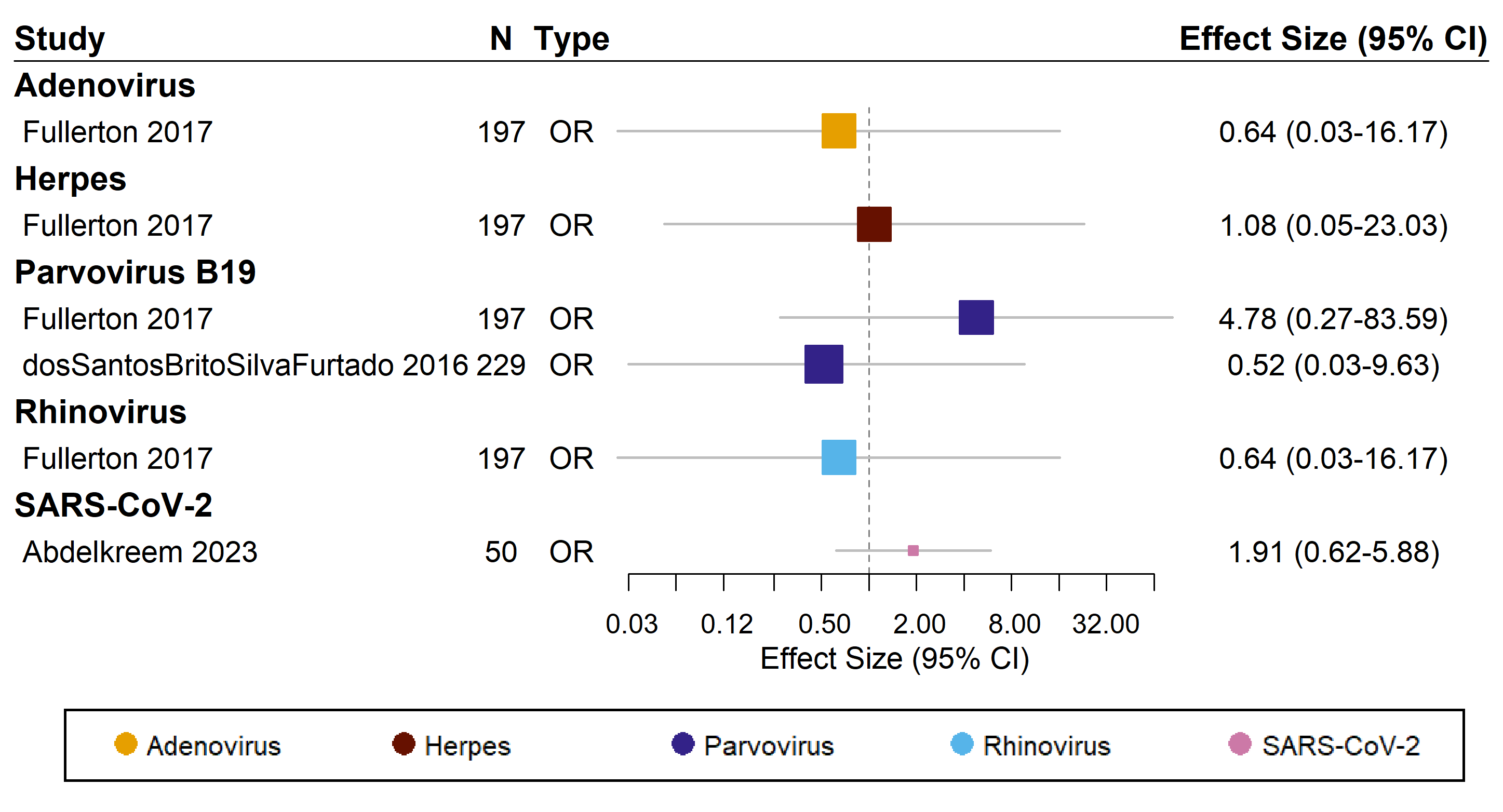


The dotted vertical line represents the line of no effect (effect size = 1). Boxes indicate reported estimates with whiskers representing 95% confidence intervals. The diamond indicates a pooled estimate from meta-analysis. OR, odds ratio.

**Supplementary Methods 1.** Protocol Changes

The protocol was first registered in PROSPERO (registration number: CRD42024494997) on [15 January 2024](https://www.crd.york.ac.uk/PROSPERO/display_record.php?RecordID=494997&VersionID=2152154). The following table lists changes to the protocol, including date and reason for the amendment.

| **Date** | **Reason for the amendment** |
| --- | --- |
| [23 April 2024](https://www.crd.york.ac.uk/PROSPERO/display_record.php?RecordID=494997&VersionID=2244964) | Clarified eligibility criteria to exclude latent virus exposures, exclude interrupted time series studies. |
|  | Added web link/DOI of preprint version of protocol on medRxiv. |
| [13 June 2024](https://www.crd.york.ac.uk/PROSPERO/display_record.php?RecordID=494997&VersionID=2291082) | Corrected a typographical error regarding the number of studies for piloting of the data collection form: changed from 'at least 50' to 'at least 5' of the included studies. |
| [17 July 2024](https://www.crd.york.ac.uk/PROSPERO/display_record.php?RecordID=494997&VersionID=2323658) | Added further detail for the risk of bias assessment and corrected the description to include a ‘very high risk of bias’ category. |
| [02 August 2024](https://www.crd.york.ac.uk/prospero/display_record.php?RecordID=494997&VersionID=2337658) | Update reference and URL for published protocol. |
|  | Review team members: adding biostatistician Eric Zhao. |

**Supplementary Methods 2.** Databases Search Strategy Results

As per protocol, our aim was to perform the search from inception until the end of March 2024 or later. Our search was updated to August 26, 2024.

**Database:** Ovid MEDLINE(R) ALL <1946 to August 23, 2024>

**Results:** 1,298

| **#** | **Query** | **Results** |
| --- | --- | --- |
| 1 | (Lab* adj2 (test* or confirm* or result* or detect* or diagnos*)).tw,kf. | 143,247 |
| 2 | exp Polymerase Chain Reaction/ | 469,075 |
| 3 | (polymerase-chain-reaction* or PCR or multi-plex or multiplex or monoplex).tw,kf. | 836,685 |
| 4 | ((Molecular or nucleic-acid or microbiologic*) adj1 (diagnos* or test* or assay*)).tw,kf. | 52,026 |
| 5 | exp Serologic Tests/ or exp microbiological techniques/ or exp fluorescent antibody technique/ | 648,102 |
| 6 | (serum* or serolog* or blood-sample or (sero* adj1 (positive* or negative* or epidemiolog*))).tw,kf. | 1,354,004 |
| 7 | (respiratory-panel* or pathogen-panel* or RP-panel* or respiratory-pathogen*).tw,kf. | 6,528 |
| 8 | (((throat* or nasal* or nasopharyngeal or naso-pharyngeal or respiratory) adj2 (swab* or specimen* or sample* or wash*)) or nasopharyngeal-aspirate or naso-pharyngeal-aspirate).tw,kf. | 26,656 |
| 9 | (culture* or cultivat* or isolat*).tw,kf. | 2,655,263 |
| 10 | (antigen-detect* or antibody-test* or immunoassay* or immuno-assay* or immunofluorescen* or immuno-fluorescen* or electron-microscopy or ELISA).tw,kf. | 748,559 |
| 11 | or/1-10 | 5,446,295 |
| 12 | exp Respiratory Tract Infections/ | 664,250 |
| 13 | (((chest or respiratory or lung) adj2 infect*) or RTI or URTI or LRTI or acute-respiratory or common-cold*).tw,kf. | 172,343 |
| 14 | exp *Virus Diseases/ or exp *Viruses/ | 1,504,198 |
| 15 | (viral* or virus* or virology).tw,kf. | 1,115,609 |
| 16 | exp influenza A virus/ or Influenza B virus/ or Influenza, Human/ | 85,376 |
| 17 | (influenza* or flu).tw,kf. | 154,032 |
| 18 | exp Coronaviridae/ or exp coronavirus infections/ | 297,878 |
| 19 | (SARS-CoV-2 or covid* or coronavirus* or severe-acute-respiratory-syndrome* or SARS or Middle-East-respiratory-syndrome* or MERS).tw,kf. | 456,642 |
| 20 | exp Picornaviridae/ or exp Picornaviridae Infections/ | 88,634 |
| 21 | (picornavirus* or rhinovirus*or parechovirus* or echovirus* or enterovirus*).tw,kf. | 16,603 |
| 22 | exp Paramyxoviridae/ or exp Paramyxoviridae Infections/ | 61,524 |
| 23 | Metapneumovirus/ or exp Pneumovirus Infections/ | 10,989 |
| 24 | (Metapneumovirus* or HMPV).tw,kf. | 2,874 |
| 25 | exp parainfluenza virus 1, human/ or exp parainfluenza virus 3, human/ | 3,971 |
| 26 | parainfluenza*.tw,kf. | 7,225 |
| 27 | Respiratory Syncytial Virus, Human/ or Respiratory Syncytial Virus Infections/ | 10,514 |
| 28 | (RSV or respiratory-syncytial).tw,kf. | 23,727 |
| 29 | exp Adenoviridae/ or exp Adenovirus Infections, Human/ | 39,114 |
| 30 | (Adenovirus* or adenoviridae).tw,kf. | 51,632 |
| 31 | exp Herpesviridae/ or exp Herpesviridae Infections/ | 185,181 |
| 32 | (herpes* or epstein-barr* or EBV or cytomegalovirus* or CMV or varicella or VZV).tw,kf. | 191,763 |
| 33 | Human bocavirus/ or exp Parvoviridae Infections/ | 6,853 |
| 34 | (human-bocavirus* or HBoV).tw,kf. | 996 |
| 35 | or/12-34 | 2,457,102 |
| 36 | exp Myocardial Infarction/ | 198,855 |
| 37 | (AMI or MI or STEMI or nSTEMI).tw,kf. | 101,274 |
| 38 | ((coronary or cardiovascular or heart or myocardial or cardiac) adj2 (infarct* or isch?emi* or attack*)).tw,kf. | 322,459 |
| 39 | ((isch?emi* or thrombo*) adj2 event*).tw,kf. | 37,706 |
| 40 | exp Stroke/ or exp Brain Infarction/ or Cerebral Hemorrhage/ or exp Subarachnoid Haemorrhage/ | 214,853 |
| 41 | (stroke* or ((brain or cerebr* or isch?emi* or h?emorrhag*) adj2 (stroke* or infarct* or failure*))).tw,kf. | 375,294 |
| 42 | ((cardiovascular or cerebr*) adj1 (acute or mortalit* or death*)).tw,kf. | 40,145 |
| 43 | or/36-42 | 824,839 |
| 44 | randomized controlled trial.pt. | 619,647 |
| 45 | (random$ or placebo$ or single blind$ or double blind$ or triple blind$).ti,ab. | 1,653,535 |
| 46 | (retraction of publication or retracted publication).pt. | 47,932 |
| 47 | or/44-46 | 1,802,566 |
| 48 | ((comment or editorial or meta-analysis or practice-guideline or review or letter) not "randomized controlled trial").pt. | 5,748,877 |
| 49 | (random sampl$ or random digit$ or random effect$ or random survey or random regression).ti,ab. not "randomized controlled trial".pt. | 139,228 |
| 50 | 47 not (48 or 49) | 1,460,567 |
| 51 | exp cohort studies/ | 2,641,335 |
| 52 | cohort$.tw. | 965,962 |
| 53 | controlled clinical trial.pt. | 95,592 |
| 54 | epidemiologic methods/ | 31,625 |
| 55 | limit 54 to yr=1966-1989 | 11,199 |
| 56 | exp case-control studies/ | 1,530,257 |
| 57 | (case$ and control$).tw. | 642,844 |
| 58 | (case$ and (series or crossover or cross-over)).tw. | 256,115 |
| 59 | or/51-53,55-58 | 3,941,056 |
| 60 | (exp animals/ or (rat or rats or mouse or mice or rodent* or swine or porcine or murine or sheep or lamb or lambs or pig or pigs or piglet or piglets or rabbit or rabbits or cat or cats or dog or dogs or cattle or bovine or monkey or monkeys or trout or marmoset or marmosets).ti.) not human*.sh. | 5,413,164 |
| 61 | 11 and 35 and 43 and (50 or 59) | 1,369 |
| 62 | 61 not 60 | 1,298 |

**Database:** Embase Classic+Embase <1947 to 2024 August 23>

**Results:** 4,801

| **#** | **Query** | **Results** |
| --- | --- | --- |
| 1 | (Lab* adj2 (test* or confirm* or result* or detect* or diagnos*)).tw,kf,dq. | 236,855 |
| 2 | exp Polymerase Chain Reaction/ | 1,306,008 |
| 3 | (polymerase-chain-reaction* or PCR or multi-plex or multiplex or monoplex).tw,kf,dq. | 1,165,705 |
| 4 | ((Molecular or nucleic-acid or microbiologic*) adj1 (diagnos* or test* or assay*)).tw,kf,dq. | 77,958 |
| 5 | exp serology/ or exp microbiological examination/ or exp fluorescent antibody technique/ | 927,965 |
| 6 | (serum* or serolog* or blood-sample or (sero* adj1 (positive* or negative* or epidemiolog*))).tw,kf,dq. | 1,973,030 |
| 7 | (respiratory-panel* or pathogen-panel* or RP-panel* or respiratory-pathogen*).tw,kf,dq. | 8,900 |
| 8 | (((throat* or nasal* or nasopharyngeal or naso-pharyngeal or respiratory) adj2 (swab* or specimen* or sample* or wash*)) or nasopharyngeal-aspirate or naso-pharyngeal-aspirate).tw,kf,dq. | 37,693 |
| 9 | (culture* or cultivat* or isolat*).tw,kf,dq. | 3,414,311 |
| 10 | (antigen-detect* or antibody-test* or immunoassay* or immuno-assay* or immunofluorescen* or immuno-fluorescen* or electron-microscopy or ELISA).tw,kf,dq. | 1,002,930 |
| 11 | or/1-10 | 7,375,991 |
| 12 | *respiratory tract infection/ or exp *lower respiratory tract infection/ or exp *upper respiratory tract infection/ or exp *viral respiratory tract infection/ | 274,842 |
| 13 | (((chest or respiratory or lung) adj2 infect*) or RTI or URTI or LRTI or acute-respiratory or common-cold*).tw,kf,dq. | 241,006 |
| 14 | exp *virus infection/ or exp *virus/ | 1,960,360 |
| 15 | (viral* or virus* or virology).tw,kf,dq. | 1,414,709 |
| 16 | *influenza/ or exp *influenza a/ or *influenza b/ or *influenza pneumonia/ or exp *pandemic influenza/ or *seasonal influenza/ | 63,448 |
| 17 | (influenza* or flu).tw,kf,dq. | 197,760 |
| 18 | *coronaviridae/ or exp *coronavirinae/ or exp *Coronavirus infection/ | 376,424 |
| 19 | (SARS-CoV-2 or covid* or coronavirus* or severe-acute-respiratory-syndrome* or SARS or Middle-East-respiratory-syndrome* or MERS).tw,kf,dq. | 532,459 |
| 20 | exp *picornaviridae/ or exp *picornavirus infection/ | 73,277 |
| 21 | (picornavirus* or rhinovirus* or parechovirus* or echovirus* or enterovirus*).tw,kf,dq. | 30,988 |
| 22 | exp *paramyxoviridae/ or exp *paramyxovirus infection/ | 40,758 |
| 23 | exp *human metapneumovirus/ or exp *human metapneumovirus infection/ | 1,067 |
| 24 | (Metapneumovirus* or HMPV).tw,kf,dq. | 4,233 |
| 25 | *respirovirus/ or *human parainfluenza virus 1/ or *human parainfluenza virus 3/ or exp *Parainfluenza virus infection/ | 672 |
| 26 | parainfluenza*.tw,kf,dq. | 10,108 |
| 27 | exp *Human respiratory syncytial virus/ or exp *respiratory syncytial virus infection/ | 7,504 |
| 28 | (RSV or respiratory-syncytial).tw,kf,dq. | 31,870 |
| 29 | exp *adenoviridae/ or exp *human adenovirus infection/ | 5,080 |
| 30 | (Adenovirus* or adenoviridae).tw,kf,dq. | 68,921 |
| 31 | exp *herpesviridae/ or exp *herpes virus infection/ | 169,961 |
| 32 | (herpes* or epstein-barr* or EBV or cytomegalovirus* or CMV or varicella or VZV).tw,kf,dq. | 262,731 |
| 33 | exp *bocaparvovirus/ or exp *Bocavirus infection/ | 479 |
| 34 | (human-bocavirus* or HBoV).tw,kf,dq. | 1,281 |
| 35 | or/12-34 | 3,002,995 |
| 36 | exp heart infarction/ | 498,528 |
| 37 | (AMI or MI or STEMI or nSTEMI).tw,kf,dq. | 188,654 |
| 38 | ((coronary or cardiovascular or heart or myocardial or cardiac) adj2 (infarct* or isch?emi* or attack*)).tw,kf,dq. | 493,085 |
| 39 | ((isch?emi* or thrombo*) adj2 event*).tw,kf,dq. | 65,405 |
| 40 | exp cerebrovascular accident/ or exp brain hemorrhage/ or exp brain infarction/ or exp brain ischemia/ | 706,564 |
| 41 | (stroke* or ((brain or cerebr* or isch?emi* or h?emorrhag*) adj2 (stroke* or infarct* or failure*))).tw,kf,dq. | 607,510 |
| 42 | ((cardiovascular or coronary or cerebrovascular) adj1 (mortalit* or death*)).tw,kf,dq. | 51,532 |
| 43 | or/36-42 | 1,564,761 |
| 44 | (random$ or placebo$ or single blind$ or double blind$ or triple blind$).ti,ab. | 2,284,895 |
| 45 | RETRACTION NOTICE/ | 13,406 |
| 46 | or/44-45 | 2,297,922 |
| 47 | (book or conference paper or editorial or letter or review).pt. not exp randomized controlled trial/ | 6,207,456 |
| 48 | (random sampl$ or random digit$ or random effect$ or random survey or random regression).ti,ab. not exp randomized controlled trial/ | 177,746 |
| 49 | 46 not (47 or 48) | 1,950,485 |
| 50 | exp cohort analysis/ | 1,211,068 |
| 51 | exp longitudinal study/ | 221,246 |
| 52 | exp prospective study/ | 940,449 |
| 53 | exp follow up/ | 2,280,157 |
| 54 | cohort$.tw. | 1,626,521 |
| 55 | exp case control study/ | 241,292 |
| 56 | limit 54 to yr=1966-1989 | 7,461 |
| 57 | exp case-control studies/ | 241,292 |
| 58 | (case$ and control$).tw. | 981,056 |
| 59 | exp case study/ | 112,616 |
| 60 | (case$ and (series or crossover or cross-over)).tw. | 400,700 |
| 61 | or/50-60 | 5,472,075 |
| 62 | (rat or rats or mouse or mice or rodent* or swine or porcine or murine or sheep or lamb or lambs or pig or pigs or piglet or piglets or rabbit or rabbits or cat or cats or dog or dogs or cattle or bovine or monkey or monkeys or trout or marmoset or marmosets).ti. and animal experiment/ | 1,302,071 |
| 63 | Animal experiment/ not (human experiment/ or human/) | 2,668,117 |
| 64 | or/62-63 | 2,746,606 |
| 65 | 11 and 35 and 43 and (49 or 61) | 4,942 |
| 66 | 65 not 64 | 4,801 |

**Database:** PubMed (National Library of Medicine)

**Date searched:** 26 Aug 2024

**Results**: 685

| **#** | **Query** | **Results** |
| --- | --- | --- |
| 1 | "Lab test"[tiab:~2] OR "Lab tests"[tiab:~2] OR "Lab testing"[tiab:~2] OR "Lab result"[tiab:~2] OR "Lab results"[tiab:~2] OR "Lab detection"[tiab:~2] OR "Lab detections"[tiab:~2] OR "Lab detect"[tiab:~2] OR "Lab diagnosis"[tiab:~2] OR "Lab diagnosed"[tiab:~2] OR "Lab diagnoses"[tiab:~2] OR "Laboratory test"[tiab:~2] OR "Laboratory tests"[tiab:~2] OR "Laboratory testing"[tiab:~2] OR "Laboratory confirmation"[tiab:~2] OR "Laboratory confirmed"[tiab:~2] OR "Laboratory confirmatory"[tiab:~2] OR "Laboratory confirm"[tiab:~2] OR "Laboratory confirmed"[tiab:~2] OR "Laboratory result"[tiab:~2] OR "Laboratory results"[tiab:~2] OR "Laboratory detect"[tiab:~2] OR "Laboratory detected"[tiab:~2] OR "Laboratory detection"[tiab:~2] OR "Laboratory detections"[tiab:~2] OR "Laboratory diagnosis"[tiab:~2] OR "Laboratory diagnosed"[tiab:~2] OR "Laboratory diagnoses"[tiab:~2] | 130,741 |
| 2 | "polymerase chain-reaction*"[tiab] OR "PCR"[tiab] OR "multi plex"[tiab] OR "multiplex"[tiab] OR "monoplex"[tiab] | 835,773 |
| 3 | "Molecular diagnosis"[tiab:~1] OR "Molecular diagnoses"[tiab:~1] OR "Molecular diagnosed"[tiab:~1] OR "Molecular diagnostic"[tiab:~1] OR "Molecular test"[tiab:~1] OR "Molecular tests"[tiab:~1] OR "Molecular testing"[tiab:~1] OR "Molecular tested"[tiab:~1] OR "Molecular assay"[tiab:~1] OR "Molecular assays"[tiab:~1] OR "Nucleic acid diagnosis"[tiab:~1] OR "Nucleic acid diagnoses"[tiab:~1] OR "Nucleic acid diagnosed"[tiab:~1] OR "Nucleic acid diagnostic"[tiab:~1] OR "Nucleic acid test"[tiab:~1] OR "Nucleic acid tests"[tiab:~1] OR "Nucleic acid testing"[tiab:~1] OR "Nucleic acid tested"[tiab:~1] OR "Nucleic acid assay"[tiab:~1] OR "Nucleic acid assays"[tiab:~1] OR "Microbiologic diagnosis"[tiab:~1] OR "Microbiologic diagnoses"[tiab:~1] OR "Microbiologic diagnosed"[tiab:~1] OR "Microbiologic diagnostic"[tiab:~1] OR "Microbiologic test"[tiab:~1] OR "Microbiologic tests"[tiab:~1] OR "Microbiologic testing"[tiab:~1] OR "Microbiologic tested"[tiab:~1] OR "Microbiologic assay"[tiab:~1] OR "Microbiologic assays"[tiab:~1] OR "Microbiological diagnosis"[tiab:~1] OR "Microbiological diagnoses"[tiab:~1] OR "Microbiological diagnosed"[tiab:~1] OR "Microbiological diagnostic"[tiab:~1] OR "Microbiological test"[tiab:~1] OR "Microbiological tests"[tiab:~1] OR "Microbiological testing"[tiab:~1] OR "Microbiological tested"[tiab:~1] OR "Microbiological assay"[tiab:~1] OR "Microbiological assays"[tiab:~1] | 60,832 |
| 4 | "Serum"[tiab] OR "Serological"[tiab] OR "Serology"[tiab] OR "Serologic"[tiab] OR "blood sample"[tiab] OR "Seroepidemiology"[tiab] OR "Seroepidemiological"[tiab] OR "Sero positive"[tiab:~1] OR "Sero negative"[tiab:~1] OR "Sero epidemiology"[tiab] OR "Sero epidemiological"[tiab] OR "Seroepidemiologic"[tiab] OR "Sero epidemiologic"[tiab] | 1,344,018 |
| 5 | "respiratory panel*"[tiab] OR "pathogen panel*"[tiab] OR "RP panel*"[tiab] OR "respiratory pathogen*"[tiab] | 6,525 |
| 6 | "Throat swab"[tiab:~2] OR "Throat swabbing" [tiab:~2] OR "Throat swabs"[tiab:~2] OR "Throat specimen"[tiab:~2] OR "Throat specimens"[tiab:~2] OR "Throat sample"[tiab:~2] OR "Throat samples"[tiab:~2] OR "Throat wash"[tiab:~2] OR "Throat washing"[tiab:~2] OR "Throat washes"[tiab:~2] OR "Nasal swab"[tiab:~2] OR "Nasal swabbing"[tiab:~2] OR "Nasal swabs"[tiab:~2] OR "Nasal specimen"[tiab:~2] OR "Nasal specimens"[tiab:~2] OR "Nasal sample"[tiab:~2] OR "Nasal samples"[tiab:~2] OR "Nasal wash"[tiab:~2] OR "Nasal washing"[tiab:~2] OR "Nasal washes" OR "Nasopharyngeal swab"[tiab:~2] OR "Nasopharyngeal swabbing"[tiab:~2] OR "Nasopharyngeal swabs"[tiab:~2] OR "Nasopharyngeal specimen"[tiab:~2] OR "Nasopharyngeal specimens"[tiab:~2] OR "Nasopharyngeal sample"[tiab:~2] OR "Nasopharyngeal samples"[tiab:~2] OR "Nasopharyngeal wash"[tiab:~2] OR "Nasopharyngeal washing"[tiab:~2] OR "Nasopharyngeal washes"[tiab:~2] OR "Naso pharyngeal swabbing"[tiab:~2] OR "Naso pharyngeal swabs"[tiab:~2] OR "Naso pharyngeal specimen"[tiab:~2] OR "Naso pharyngeal specimens"[tiab:~2] OR "Naso pharyngeal sample"[tiab:~2] OR "Naso pharyngeal samples"[tiab:~2] OR "Naso pharyngeal wash"[tiab:~2] OR "Naso pharyngeal washing"[tiab:~2] OR "Naso pharyngeal washes"[tiab:~2] OR "Respiratory swab"[tiab:~2] OR "Respiratory swabbing" [tiab:~2] OR "Respiratory swabs"[tiab:~2] OR "Respiratory specimen"[tiab:~2] OR "Respiratory specimens"[tiab:~2] OR "Respiratory sample"[tiab:~2] OR "Respiratory samples"[tiab:~2] OR "Nasopharyngeal aspirate"[tiab] | 28,318 |
| 7 | "culture"[tiab] OR "cultivat*"[tiab] OR "isolat*"[tiab] | 2,253,266 |
| 8 | "antigen detect*"[tiab] OR "antibody test*"[tiab] OR "immunoassay*"[tiab] OR "immuno assay*"[tiab] OR "immunofluorescen*"[tiab] OR "electron microscopy"[tiab] OR "ELISA"[tiab] | 747,964 |
| 9 | #1 OR #2 OR #3 OR #4 OR #5 OR #6 OR #7 OR #8 | 4,670,128 |
| 10 | "chest infection"[tiab:~2] OR "chest infections"[tiab:~2] OR "chest infected"[tiab:~2] OR "respiratory infection"[tiab:~2] OR "respiratory infections"[tiab:~2] OR "respiratory infected"[tiab:~2] OR "lung infection"[tiab:~2] OR "lung infections"[tiab:~2] OR "lung infected"[tiab:~2] OR RTI[tiab] OR URTI[tiab] OR LRTI[tiab] OR "acute respiratory*"[tiab] OR "common cold*"[tiab] | 178,960 |
| 11 | "viral*"[tiab] OR "virus*"[tiab] OR "virology"[tiab] | 1,114,896 |
| 12 | "influenza*"[tiab] OR "flu"[tiab] | 153,790 |
| 13 | "SARS-CoV-2"[tiab] OR "covid*"[tiab] OR "coronavirus*"[tiab] OR "severe acute respiratory syndrome*"[tiab] OR "SARS"[tiab] OR "Middle East respiratory syndrome*"[tiab] OR "MERS"[tiab] | 455,601 |
| 14 | "picornavirus*"[tiab] OR "rhinovirus*" OR "parechovirus*"[tiab] OR "echovirus*"[tiab] OR "enterovirus*"[tiab] | 23,316 |
| 15 | "Metapneumovirus*"[tiab] OR "HMPV"[tiab] | 2,873 |
| 16 | "parainfluenza*"[tiab] | 7,499 |
| 17 | "RSV"[tiab] OR "respiratory syncytial"[tiab] | 23,755 |
| 18 | "Adenovirus*"[tiab] OR "Adenoviridae"[tiab] | 51,649 |
| 19 | "herpes*"[tiab] OR "epstein-barr*"[tiab] OR "EBV"[tiab] OR "cytomegalovirus*"[tiab] OR "CMV"[tiab] OR "varicella"[tiab] OR "VZV"[tiab] | 191,878 |
| 20 | "human bocavirus*"[tiab] or "HBoV"[tiab] | 994 |
| 21 | #10 OR #11 OR #12 OR #13 OR #14 OR #15 OR #16 OR #17 OR #18 OR #19 OR #20 | 1,719,350 |
| 22 | "AMI"[tiab] OR "MI"[tiab] OR "STEMI"[tiab] OR "nSTEMI"[tiab] | 101,466 |
| 23 | "coronary infarct"[tiab:~2] OR "coronary infarction"[tiab:~2] OR "coronary infarctions"[tiab:~2] OR "coronary ischaemia"[tiab:~2] OR "coronary ischemia"[tiab:~2] OR "coronary ischaemic"[tiab:~2] OR "coronary ischemic"[tiab:~2] OR "coronary attack"[tiab:~2] OR "coronary attacks"[tiab:~2] OR "cardiovascular infarct"[tiab:~2] OR "cardiovascular infarction"[tiab:~2] OR "cardiovascular infarctions"[tiab:~2] OR "cardiovascular ischaemia"[tiab:~2] OR "cardiovascular ischemia"[tiab:~2] OR "cardiovascular ischaemic"[tiab:~2] OR "cardiovascular ischemic"[tiab:~2] OR "cardiovascular attack"[tiab:~2] OR "cardiovascular attacks"[tiab:~2] OR "heart infarct"[tiab:~2] OR "heart infarction"[tiab:~2] OR "heart infarctions"[tiab:~2] OR "heart ischaemia"[tiab:~2] OR "heart ischemia"[tiab:~2] OR "heart ischaemic"[tiab:~2] OR "heart ischemic"[tiab:~2] OR "heart attack"[tiab:~2] OR "heart attacks"[tiab:~2] OR "myocardial infarct"[tiab:~2] OR "myocardial infarction"[tiab:~2] OR "myocardial infarctions"[tiab:~2] OR "myocardial ischaemia"[tiab:~2] OR "myocardial ischemia"[tiab:~2] OR "myocardial ischaemic"[tiab:~2] OR "myocardial ischemic"[tiab:~2] OR "myocardial attack"[tiab:~2] OR "myocardial attacks"[tiab:~2] OR "cardiac infarct"[tiab:~2] OR "cardiac infarction"[tiab:~2] OR "cardiac infarctions"[tiab:~2] OR "cardiac ischaemia"[tiab:~2] OR "cardiac ischemia"[tiab:~2] OR "cardiac ischaemic"[tiab:~2] OR "cardiac ischemic"[tiab:~2] OR "cardiac attack"[tiab:~2] OR "cardiac attacks"[tiab:~2] | 329,058 |
| 24 | "ischaemic event"[tiab:~2] OR "ischaemic events"[tiab:~2] OR "ischemic event"[tiab:~2] OR "ischemic events"[tiab:~2] OR "ischaemia event"[tiab:~2] OR "ischaemia events"[tiab:~2] OR "ischemia events"[tiab:~2] OR "ischemia events"[tiab:~2] OR "thrombotic event"[tiab:~2] OR "thrombosis event"[tiab:~2] OR "thrombotic events"[tiab:~2] OR "thrombosis events"[tiab:~2] | 25,180 |
| 25 | stroke*[tiab] OR "brain infarct"[tiab:~2] OR "brain infarction"[tiab:~2] OR "brain infarctions"[tiab:~2] OR "brain failure"[tiab:~2] OR "brain failures"[tiab:~2] OR "brain haemorrhage"[tiab:~2] OR "brain hemorrhage"[tiab:~2] OR "cerebral infarct"[tiab:~2] OR "cerebral infarction"[tiab:~2] OR "cerebral infarctions"[tiab:~2] OR "cerebral failure"[tiab:~2] OR "cerebral failures"[tiab:~2] OR "cerebral stroke"[tiab:~2] OR "cerebral strokes"[tiab:~2] OR "cerebrovascular infarct"[tiab:~2] OR "cerebrovascular infarction"[tiab:~2] OR "cerebrovascular infarctions"[tiab:~2] OR "cerebrovascular failure"[tiab:~2] OR "cerebrovascular failures"[tiab:~2] OR "cerebrovascular stroke"[tiab:~2] OR "cerebrovascular strokes"[tiab:~2] OR "ischaemic infarct"[tiab:~2] OR "ischaemic infarction"[tiab:~2] OR "ischaemic infarctions"[tiab:~2] OR "ischaemic failure"[tiab:~2] OR "ischaemic failures"[tiab:~2] OR "ischaemic stroke"[tiab:~2] OR "ischaemic strokes"[tiab:~2] OR "ischemic infarct"[tiab:~2] OR "ischemic infarction"[tiab:~2] OR "ischemic infarctions"[tiab:~2] OR "ischemic failure"[tiab:~2] OR "ischemic failures"[tiab:~2] OR "ischemic stroke"[tiab:~2] OR "ischemic strokes"[tiab:~2] OR "haemorrhagic infarct"[tiab:~2] OR "haemorrhagic infarction"[tiab:~2] OR "haemorrhagic infarctions"[tiab:~2] OR "haemorrhagic failure"[tiab:~2] OR "haemorrhagic failures"[tiab:~2] OR "haemorrhagic stroke"[tiab:~2] OR "haemorrhagic strokes"[tiab:~2] OR "hemorrhagic infarct"[tiab:~2] OR "hemorrhagic infarction"[tiab:~2] OR "hemorrhagic infarctions"[tiab:~2] OR "hemorrhagic failure"[tiab:~2] OR "hemorrhagic failures"[tiab:~2] OR "hemorrhagic stroke"[tiab:~2] OR "hemorrhagic strokes"[tiab:~2] OR "haemorrhage infarct"[tiab:~2] OR "haemorrhage infarction"[tiab:~2] OR "haemorrhage infarctions"[tiab:~2] OR "haemorrhage failure"[tiab:~2] OR "haemorrhage failures"[tiab:~2] OR "haemorrhage stroke"[tiab:~2] OR "haemorrhage strokes"[tiab:~2] OR "hemorrhage infarct"[tiab:~2] OR "hemorrhage infarction"[tiab:~2] OR "hemorrhage infarctions"[tiab:~2] OR "hemorrhage stroke"[tiab:~2] OR "hemorrhage strokes"[tiab:~2] | 375,827 |
| 26 | "cardiovascular acute"[tiab:~1] OR "cardiovascular mortality"[tiab:~1] OR "cardiovascular mortalities"[tiab:~1] OR "cardiovascular death"[tiab:~1] OR "cardiovascular deaths"[tiab:~1] OR "cerebral acute"[tiab:~1] OR "cerebral mortality"[tiab:~1] OR "cerebral death"[tiab:~1] OR "cerebral deaths"[tiab:~1] OR "cerebrovascular acute"[tiab:~1] OR "cerebrovascular mortality"[tiab:~1] OR "cerebrovascular mortalities"[tiab:~1] OR "cerebrovascular death"[tiab:~1] OR "cerebrovascular deaths"[tiab:~1] | 54,110 |
| 27 | #22 OR #23 OR #24 OR #25 OR #26 | 728,112 |
| 28 | NOTNLM OR publisher[sb] OR inprocess[sb] OR pubmednotmedline[sb] OR indatareview[sb] OR pubstatusaheadofprint | 12,121,713 |
| 29 | (((((randomized controlled trial[Publication Type]) OR (random*[Title/Abstract] OR placebo*[Title/Abstract] OR single blind*[Title/Abstract] OR double blind*[Title/Abstract] OR triple blind*[Title/Abstract]) OR (retraction of publication[Publication Type] OR retracted publication[Publication Type])) NOT (((comment[Publication Type] OR editorial[Publication Type] OR meta-analysis[Publication Type] OR practice-guideline[Publication Type] OR review[Publication Type] OR letter[Publication Type]) NOT randomized controlled trial[Publication Type]) OR ((random sampl*[Title/Abstract] OR random digit*[Title/Abstract] OR random effect*[Title/Abstract] OR random survey[Title/Abstract] OR random regression[Title/Abstract]) NOT randomized controlled trial[Publication Type]))) OR (cohort OR controlled-clinical-trial* OR epidemiolog*) OR (case* AND (control* OR series OR crossover OR cross-over))) NOT (("Animal" OR "animals" OR "rat" OR "rats" OR "mouse" OR "mice" OR "rodent*" OR "swine" OR "porcine" OR "murine" OR "sheep" OR "lamb" OR "lambs" OR "pig" OR "pigs" OR "piglet" OR "piglets" OR "rabbit" OR "rabbits" OR "cat" OR "cats" OR "dog" OR "dogs" OR "cattle" OR "bovine" OR "monkey" OR "monkeys" OR "trout" OR "marmoset" OR "marmosets") NOT ("human" OR "humans" OR "patient" OR "patients" OR "newborn*" OR "baby" OR "babies" OR "neonat*" OR "infan*" OR "toddler*" OR "pre-schooler*" OR "preschooler*" OR "kindergarten" OR "boy" OR "boys" OR "girl" OR "girls" OR "child" OR "children" OR "childhood" OR "adolescen*" OR "pediatric*" OR "paediatric*" OR "youth*" OR "teen" OR "teens" OR "teenage*" OR "school-aged*" OR "school-child*" OR "school-girl*" OR "school-boy*" OR "schoolgirl*" OR "schoolboy*" OR "man" OR "men" OR "woman" OR "women" OR "adult" OR "adults" OR "middle-age*" OR "elderly"))) | 5,490,353 |
| 30 | #9 AND #21 AND #27 AND #28 AND #29 | 685 |

**Database: Cochrane Central Register of Controlled Trials**

**Date Run:** 26/08/2024 02:40:18

**Results:** 413 Trials matching "#44 - #11 AND #35 AND #43" in Trials

Issue 7 of 12, July 2024

| **ID** | **Search** | **Hits** |
| --- | --- | --- |
| #1 | (Lab*:ti,ab,kw NEAR/2 (test*:ti,ab,kw OR confirm*:ti,ab,kw OR result*:ti,ab,kw OR detect*:ti,ab,kw OR diagnos*:ti,ab,kw)) | 23040 |
| #2 | [mh "Polymerase Chain Reaction"] | 2846 |
| #3 | (polymerase-chain-reaction*:ti,ab,kw OR PCR:ti,ab,kw OR multi-plex:ti,ab,kw OR multiplex:ti,ab,kw OR monoplex:ti,ab,kw) | 23230 |
| #4 | ((Molecular:ti,ab,kw OR nucleic-acid:ti,ab,kw OR microbiologic*:ti,ab,kw) NEAR/1 (diagnos*:ti,ab,kw OR test*:ti,ab,kw OR assay*:ti,ab,kw)) | 1612 |
| #5 | [mh "Serologic Tests"] OR [mh "microbiological techniques"] OR [mh "fluorescent antibody technique"] | 10338 |
| #6 | (serum*:ti,ab,kw OR serolog*:ti,ab,kw OR blood-sample:ti,ab,kw OR (sero*:ti,ab,kw NEAR/1 (positive*:ti,ab,kw OR negative*:ti,ab,kw OR epidemiolog*:ti,ab,kw))) | 137849 |
| #7 | (respiratory-panel*:ti,ab,kw OR pathogen-panel*:ti,ab,kw OR RP-panel*:ti,ab,kw OR respiratory-pathogen*:ti,ab,kw) | 300 |
| #8 | (((throat*:ti,ab,kw OR nasal*:ti,ab,kw OR nasopharyngeal:ti,ab,kw OR naso-pharyngeal:ti,ab,kw OR respiratory:ti,ab,kw) NEAR/2 (swab*:ti,ab,kw OR specimen*:ti,ab,kw OR sample*:ti,ab,kw OR wash*:ti,ab,kw)) OR nasopharyngeal-aspirate:ti,ab,kw OR naso-pharyngeal-aspirate:ti,ab,kw) | 2771 |
| #9 | (culture*:ti,ab,kw OR cultivat*:ti,ab,kw OR isolat*:ti,ab,kw) | 59033 |
| #10 | (antigen-detect*:ti,ab,kw OR antibody-test*:ti,ab,kw OR immunoassay*:ti,ab,kw OR immuno-assay*:ti,ab,kw OR immunofluorescen*:ti,ab,kw OR immuno-fluorescen*:ti,ab,kw OR electron-microscopy:ti,ab,kw OR ELISA:ti,ab,kw) | 19592 |
| #11 | #1 OR #2 OR #3 OR #4 OR #5 #6 OR #8 OR #9 OR #10 | 117643 |
| #12 | [mh "Respiratory Tract Infections"] | 27576 |
| #13 | (((chest:ti,ab,kw OR respiratory:ti,ab,kw OR lung:ti,ab,kw) NEAR/2 infect*:ti,ab,kw) OR RTI:ti,ab,kw OR URTI:ti,ab,kw OR LRTI:ti,ab,kw OR acute-respiratory:ti,ab,kw OR common-cold*:ti,ab,kw) | 24597 |
| #14 | [mh "Virus Diseases"] OR [mh Viruses] | 49327 |
| #15 | (viral*:ti,ab,kw OR virus*:ti,ab,kw OR virology:ti,ab,kw) | 55237 |
| #16 | [mh "influenza A virus"] OR [mh "influenza b virus"] OR [mh ^"Influenza, Human"] | 3860 |
| #17 | (influenza*:ti,ab,kw OR flu:ti,ab,kw) | 13367 |
| #18 | [mh Coronaviridae] OR [mh "coronavirus infections"] | 8635 |
| #19 | (SARS-CoV-2:ti,ab,kw OR covid*:ti,ab,kw OR coronavirus*:ti,ab,kw OR severe-acute-respiratory-syndrome*:ti,ab,kw OR SARS:ti,ab,kw OR Middle-East-respiratory-syndrome*:ti,ab,kw OR MERS:ti,ab,kw) | 22808 |
| #20 | [mh Picornaviridae] OR [mh "Picornaviridae Infections"] | 3039 |
| #21 | (picornavirus*:ti,ab,kw OR (rhinovirus*or NEXT parechovirus*):ti,ab,kw OR echovirus*:ti,ab,kw OR enterovirus*:ti,ab,kw) | 303 |
| #22 | [mh Paramyxoviridae] OR [mh "Paramyxoviridae Infections"] | 1177 |
| #23 | [mh ^Metapneumovirus] OR [mh "Pneumovirus Infections"] | 540 |
| #24 | (Metapneumovirus*:ti,ab,kw OR HMPV:ti,ab,kw) | 106 |
| #25 | [mh "parainfluenza virus 1, human"] OR [mh "parainfluenza virus 3, human"] | 25 |
| #26 | parainfluenza*:ti,ab,kw | 260 |
| #27 | [mh ^"Respiratory Syncytial Virus, Human"] OR [mh ^"Respiratory Syncytial Virus Infections"] | 545 |
| #28 | (RSV:ti,ab,kw OR respiratory-syncytial:ti,ab,kw) | 1595 |
| #29 | [mh Adenoviridae] OR [mh "Adenovirus Infections, Human"] | 305 |
| #30 | (Adenovirus*:ti,ab,kw OR Adenoviridae:ti,ab,kw) | 1052 |
| #31 | [mh Herpesviridae] OR [mh "Herpesviridae Infections"] | 4005 |
| #32 | (herpes*:ti,ab,kw OR epstein-barr*:ti,ab,kw OR EBV:ti,ab,kw OR cytomegalovirus*:ti,ab,kw OR CMV:ti,ab,kw OR varicella:ti,ab,kw OR VZV:ti,ab,kw) | 9684 |
| #33 | [mh ^"Human bocavirus"] OR [mh "Parvoviridae Infections"] | 28 |
| #34 | (human-bocavirus*:ti,ab,kw OR HBoV:ti,ab,kw) | 18 |
| #35 | #12 OR #13 OR #14 OR #15 OR #16 OR #17 OR #18 OR #19 OR #20 OR #21 OR #22 OR #23 OR #24 OR #25 OR #26 OR #27 OR #28 OR #29 OR #30 OR #31 OR #32 OR #33 OR #34 | 122836 |
| #36 | [mh "Myocardial Infarction"] | 15716 |
| #37 | (AMI:ti,ab,kw OR MI:ti,ab,kw OR STEMI:ti,ab,kw OR nSTEMI:ti,ab,kw) | 21062 |
| #38 | ((coronary:ti,ab,kw OR cardiovascular:ti,ab,kw OR heart:ti,ab,kw OR myocardial:ti,ab,kw OR cardiac:ti,ab,kw) NEAR/2 (infarct*:ti,ab,kw OR isch?emi*:ti,ab,kw OR attack*:ti,ab,kw)) | 54169 |
| #39 | ((isch?emi*:ti,ab,kw OR thrombo*:ti,ab,kw) NEAR/2 event*:ti,ab,kw) | 7398 |
| #40 | [mh Stroke] OR [mh "Brain Infarction"] OR [mh ^"Cerebral Hemorrhage"] OR [mh "Subarachnoid Haemorrhage"] | 18730 |
| #41 | (stroke*:ti,ab,kw OR ((brain:ti,ab,kw OR cerebr*:ti,ab,kw OR isch?emi*:ti,ab,kw OR h?emorrhag*:ti,ab,kw) NEAR/2 (stroke*:ti,ab,kw OR infarct*:ti,ab,kw OR failure*:ti,ab,kw))) | 79706 |
| #42 | ((cardiovascular:ti,ab,kw OR cerebr*:ti,ab,kw) NEAR/1 (acute:ti,ab,kw OR mortalit*:ti,ab,kw OR death*:ti,ab,kw)) | 11232 |
| #43 | #36 OR #37 OR #38 OR #39 OR #40 OR #41 OR #42 | 134688 |
| #44 | #11 AND #35 AND #43 in Trials | 413 |

**Database:** Web of Science Core Collection (Clarivate Analytics)

**Date run:** Mon Aug 26 2024

**Results**: 710

|  | **Search Query** | **Results** |
| --- | --- | --- |
| 1 | Lab* NEAR/2 (test* or confirm* or result* or detect* or diagnos*) (Title) OR Lab* NEAR/2 (test* or confirm* or result* or detect* or diagnos*) (Abstract) | 285891 |
| 2 | polymerase-chain-reaction* or PCR or multi-plex or multiplex or monoplex (Title) OR polymerase-chain-reaction* or PCR or multi-plex or multiplex or monoplex (Abstract) | 968358 |
| 3 | (Molecular or nucleic-acid or microbiologic*) NEAR/1 (diagnos* or test* or assay*) (Title) OR (Molecular or nucleic-acid or microbiologic*) NEAR/1 (diagnos* or test* or assay*) (Abstract) | 70898 |
| 4 | serum* or serolog* or blood-sample or (sero* NEAR/1 (positive* or negative* or epidemiolog*)) (Title) OR serum* or serolog* or blood-sample or (sero* NEAR/1 (positive* or negative* or epidemiolog*)) (Abstract) | 1541589 |
| 5 | respiratory-panel* or pathogen-panel* or RP-panel* or respiratory-pathogen* (Title) OR respiratory-panel* or pathogen-panel* or RP-panel* or respiratory-pathogen* (Abstract) | 6318 |
| 6 | (throat* or nasal* or nasopharyngeal or naso-pharyngeal or respiratory*) NEAR/2 (swab* or specimen* or sample* or wash*) or nasopharyngeal-aspirate or naso-pharyngeal-aspirate (Title) OR (throat* or nasal* or nasopharyngeal or naso-pharyngeal or respiratory*) NEAR/2 (swab* or specimen* or sample* or wash*) or nasopharyngeal-aspirate or naso-pharyngeal-aspirate (Abstract) | 29292 |
| 7 | culture* or cultivat* or isolat* (Title) OR culture* or cultivat* or isolat* (Abstract) | 4071978 |
| 8 | antigen-detect* or antibody-test* or immunoassay* or immuno-assay* or immunofluorescen* or immuno-fluorescen* or electron-microscopy or ELISA (Title) OR antigen-detect* or antibody-test* or immunoassay* or immuno-assay* or immunofluorescen* or immuno-fluorescen* or electron-microscopy or ELISA (Abstract) | 1239472 |
| 9 | #1 OR #2 OR #3 OR #4 OR #5 OR #6 OR #7 OR #8 | 7329366 |
| 10 | ((chest or respiratory or lung) NEAR/2 infect*) or RTI or URTI or LRTI or acute-respiratory or common-cold* (Title) OR ((chest or respiratory or lung) NEAR/2 infect*) or RTI or URTI or LRTI or acute-respiratory or common-cold* (Abstract) | 174992 |
| 11 | viral* or virus* or virology (Title) OR viral* or virus* or virology (Abstract) | 1195303 |
| 12 | influenza* or flu (Title) OR influenza* or flu (Abstract) | 160036 |
| 13 | SARS-CoV-2 or covid* or coronavirus* or severe-acute-respiratory-syndrome* or SARS or Middle-East-respiratory-syndrome* or MERS (Title) OR SARS-CoV-2 or covid* or coronavirus* or severe-acute-respiratory-syndrome* or SARS or Middle-East-respiratory-syndrome* or MERS (Abstract) | 622135 |
| 14 | picornavirus* or rhinovirus*or parechovirus* or echovirus* or enterovirus* (Title) OR picornavirus* or rhinovirus*or parechovirus* or echovirus* or enterovirus* (Abstract) | 15648 |
| 15 | Metapneumovirus* or HMPV (Title) OR Metapneumovirus* or HMPV (Abstract) | 2940 |
| 16 | parainfluenza* (Title) OR parainfluenza* (Abstract) | 6725 |
| 17 | RSV or respiratory-syncytial* (Title) OR RSV or respiratory-syncytial* (Abstract) | 25211 |
| 18 | Adenovirus* or adenoviridae (Title) OR Adenovirus* or adenoviridae (Abstract) | 53816 |
| 19 | herpes* or epstein-barr* or EBV or cytomegalovirus* or CMV or varicella or VZV (Title) OR herpes* or epstein-barr* or EBV or cytomegalovirus* or CMV or varicella or VZV (Abstract) | 202024 |
| 20 | #10 OR #11 OR #12 OR #13 OR #14 OR #15 OR #16 OR #17 OR #18 OR #19 | 1984396 |
| 21 | AMI or MI or STEMI or nSTEMI (Title) OR AMI or MI or STEMI or nSTEMI (Abstract) | 144554 |
| 22 | (coronary or cardiovascular or heart or myocardial or cardiac) NEAR/2 (infarct* or isch$emi* or attack*) (Title) OR (coronary or cardiovascular or heart or myocardial or cardiac) NEAR/2 (infarct* or isch$emi* or attack*) (Abstract) | 340283 |
| 23 | stroke* or ((brain or cerebr* or isch$emi* or h$emorrhag*) NEAR/2 (stroke* or infarct* or failure*)) (Title) OR stroke* or ((brain or cerebr* or isch$emi* or h$emorrhag*) NEAR/2 (stroke* or infarct* or failure*)) (Abstract) | 452435 |
| 24 | (cardiovascular or cerebr*) NEAR/1 (acute or mortalit* or death*) (Title) OR (cardiovascular or cerebr*) NEAR/1 (acute or mortalit* or death*) (Abstract) | 54447 |
| 25 | #21 OR #22 OR #23 OR #24 | 848304 |
| 26 | random* or placebo* or single blind* or double blind* or triple blind* or retraction or retracted or cohort* or controlled-clinical-trial* or epidemiologic or (case and (control* or series or crossover or cross-over)) (Title) OR random* or placebo* or single blind* or double blind* or triple blind* or retraction or retracted or cohort* or controlled-clinical-trial* or epidemiologic or (case and (control* or series or crossover or cross-over)) (Abstract) | 4680690 |
| 27 | #9 AND #20 AND #25 AND #26 | 710 |

**Supplementary Methods 3.** Inclusion/Exclusion Criteria List

|  | **Include** | **Exclude** |
| --- | --- | --- |
| **EXPOSURE** | **PCR or serology**  Influenza  Coronavirus  SARS-CoV-2 or COVID-19  Respiratory syncytial virus  Adenovirus  Picornaviruses including enteroviruses (including Coxsackie B, rhinovirus)  Human metapneumovirus  Parainfluenza  WU polyomavirus  Human bocavirus  Andes hantavirus  Parvovirus B19 **(PCR + symptoms) OR (IgM serology + PCR)**  Herpes simplex virus  Herpes zoster virus **(Seroconversion) OR (IgM + PCR)**  Cytomegalovirus  Epstein-Barr virus | Hemorrhagic fever Community-acquired pneumonia  HIV infection  Hepatitis viruses  Invasive pneumococcal disease Tuberculosis  *Haemophilus influenzae* *Helicobacter pylori* *Staphylococcus aureus* *Streptococcus pneumoniae Chlamydia pneumoniae* Malaria Dengue virus Pulmonary cryptococcosis  Aspergillosis Puumala hantavirus Ebola virus Simian virus Hepatin binding protein  West Nile virus Zika virus Chikungunya virus Newcastle disease virus Measles virus Rubella **IgG serology OR IgM serology-only**^a^  Herpes simplex virus  Herpes zoster virus  Cytomegalovirus  Epstein-Barr virus |
| **OUTCOME** | Myocardial infarction Stroke Ischemic stroke Hemorrhagic stroke Intracerebral thrombotic hemorrhage Subarachnoid hemorrhage Intracerebral hemorrhage | Transient ischemic attack Cardiac arrest Peripheral arterial disease Acute limb ischemia Acute mesenteric ischemia Pulmonary embolism Atrial fibrillation Acute respiratory distress syndrome Myocarditis  Encephalitis  Meningitis  Endocarditis Venous thrombotic event Deep vein thrombosis Thrombosis  Coagulopathy |
| **COMPARATOR** | Negative testing individuals Unexposed person-time | No patients without infection  COVID-19 positive versus influenza positive  Pre-pandemic vs pandemic cohorts |
| **STUDY DESIGN** | Self-controlled case series Case crossover study Randomized controlled trial Cohort study Case-control study | Case report  Case study  Clinical management report Cross-sectional study (no follow-up) Ecological study Interrupted time series Before-and-after study |
| **TIMEFRAME** | Short-term risk  Risk period within 90 days  Mention of a triggering effect | Chronic infection risk window  Long-term outcomes (years) |
|  | Effect estimate data available^b^ | Insufficient information^d,e^ |

^a^ For latent viruses e.g. Herpes family viruses, we looked for indicators of recent infection/reactivation (measured by viral nucleic acid, serum IgM).

^b^ Risk Ratio, Odds Ratio, Rate Ratio, Incidence Rate Ratio or Hazard Ratio.

^c^ No effect estimate or data not extractable, or not enough data to calculate effect estimate.

^d^ Methods insufficiently described.

^e^ Contacted authors no response within 2 weeks.

**Supplementary Methods 4.** Risk of Bias Assessment – Approach to Signaling Questions

The Cochrane Risk of Bias in Non-randomised studies – of Exposures (ROBINS-E) tool for assessing risk of bias in individual observational studies of exposure-outcome effects.^297^ As there were no randomized trials suitable for inclusion in the review, only the ROBINS-E tool was used to judge overall risk of bias for each individual study. Our risk of bias assessment pertains to the exposure-outcome association derived from the study i.e. result-level assessment for risk of bias.

| **ROBINS-E domain** |  | **Approach to Signaling Questions** |
| --- | --- | --- |
| Planning stage | P1. | Important confounders to be accounted for:   - Age, sex, socioeconomic status, comorbidities - Vaccination status (where applicable for exposure under study) |
|  | P2. | No—we did not assess appropriateness of study design. |
| Preliminary considerations | A1. | Numerical result being assessed i.e. result-level assessment for risk of bias:   - Use the *most adjusted result* and note the confounders adjusted for in Preliminary assessment E |
|  | B1. | This question is interpreted as “Did the authors use an appropriate analysis method or design that adjusted for all of the important confounding variables?”  Y/PY—if an adjusted effect estimate is presented for the specific result, including controlling by:   - statistical analysis e.g. multivariable methods, propensity score matching - stratification - restriction of enrolment - matching controlled groups e.g. case-control or matched cohort   PN—Not for result being assessed, but the authors attempted to control for confounding for all other outcomes in their study design.  N—No attempts to control for confounding for outcome of interest. |
|  | B2. | If N/PN to B1:  Is there sufficient potential for confounding that an unadjusted result should not be considered further?  N/PN—if adjusted results were presented for composite outcomes only and there is clear evidence or justification for why adjusted results for AMI/stroke were not presented e.g. cell counts too low.  Y/PY—if unadjusted results only, including derived manually. In this case, the risk of bias directly led to the result being at very high risk of bias. For example, no specific adjusted effect estimate available for AMI and/or stroke and the numerical result being assessed is based on manually derived numbers which should not be considered further. |
|  | C4. | N/PN—binary exposure for most studies e.g. “For each person, records of multiple respiratory organisms dated within 28 days of each other were de-duplicated within strata of virus or bacteria.” |
|  |  | For self-controlled case series:   - researchers will often deduplicate records of multiple respiratory organisms within specific time period. |
|  | C5. | N/PN—exposure captured at single point in time for most studies, except for seroconversion where ii) a single exposure value derived from repeated measurements on each individual.  For self-controlled case series:   - N/PN – for studies with multiple risk windows that analyse a single measurement of exposure for each individual, but individuals are split by their post-exposure risk windows. |
|  | C10 | For self-controlled case series:   - Incidence rate ratio for the outcome occurring in exposure risk period compared with their baseline period. |
|  | D4. | For self-controlled case series:   - Exposure risk period vs baseline period. |
|  | E | For **self-controlled case series:**   - Time-fixed confounders were not an issue by design   (Y/PY controlled for in the analysis if measured: sex, socioeconomic status.  These time-fixed confounders are usually measured in SCCS, i.e. evidence that controlling for it was unnecessary based on the “Table 1” demographic table.   - Time-varying confounding considered to be controlled for**, if measured for this variable,** as confounder: - Age e.g. age in 5-year strata - Seasonality e.g. calendar month, 3-month blocks   Y/PY—includes sensitivity analyses incorporating adjustment for different time interval(s)  N/PN—if the estimate was not adjusted for at all without reason (if applicable)   - Vaccination status:   Y/PY—Were this variable controlled for in the analysis?  e.g. as long as vaccination status was measured in this study.   - restriction of enrolment - subgroup analysis   N/PN—if the estimate was not adjusted for vaccination status (if applicable). |
| **Domain 1 (confounding)** |  | Anly variant (a) used. |
|  | 1.1 | Y—if an adjusted effect estimate is presented for the specific result as specified in Preliminary considerations A1.  PY—if the authors described attempt to control for confounding but results in the multivariable analysis but there was evidence that controlling for it was unnecessary e.g. low cell counts for a composite outcome, methods or supplementary material describes attempts to control for confounding, but data are not presented for specified reason. |
|  | 1.2 | For **self-controlled case series**,   - If applicable/relevant to the circulation of the virus/exposure measured (i.e. influenza, RSV) - Measurement of calendar time / seasonality valid if an attempt was made to adjust for the confounding factor e.g., season in 3-month categories. |
|  | 1.4 | *‘Negative controls’* for outcomes or exposures can be helpful to assess the likely presence of unmeasured or residual confounding.  For **cohort studies:**   - E.g. vaccination against influenza not only protected against hospitalisation for pneumonia but also against hospitalisation for injury or trauma   For **self-controlled case series:**   - SCCS studies can examine the association of exposures with negative control outcomes e.g. coeliac disease, that are not thought to be related to the exposure   For **case-control studies:**   - Did the control group without AMI/stroke, or other considerations i.e. matching on factors with the ‘case’ group, suggest serious uncontrolled confounding? e.g. There is evidence that the distribution of important confounders differs substantially between cases and matched controls. |
| Domain 2 (measurement of exposure) | - | In most cases, only variant (a) was used: exposure as measured at a single point in time for all studies, unless serology was done.   - This question applies to the type of laboratory test used is appropriate for the virus, risk window used is reasonable with known understanding of pathophysiology |
|  | 2.1 | For **self-controlled case series:**  “Does the measured exposure well-characterize the exposure metric specified to be of interest in this study?”   - Interpret question 2.1 as “Were valid methods used for identification of the condition for all participants included in the case series?”   2.1.1 Additional question for **self-controlled case series**:  Are the defined risk window(s)/interval(s) e.g. pre-exposure, post-exposure, defined appropriately for the specified exposure of interest in this study? (*Regardless of answer proceed to question 2.2*) |
| Domain 3 (selection of participants into the study) | 3.1 | For **self-controlled case series:**  Y/PY if:   - The authors have accounted for latency or lag time (if applicable) with clearly defined pre- and post-exposure intervals - Beginning of the follow-up interpreted as the index date - e.g. reasonable inclusion or exclusion of patients based on specimen collection date   N/PN if:   - Evidence of selection biases due to definition of the ‘index date’ or exposure interval |
|  | 3.3 | For **self-controlled case series:**  Y/PY if:   - If the case series have consecutive inclusion of participants and the authors provided clear inclusion/exclusion criteria for their study participants with sufficient detail study - For example, a case series that states ‘we included all patients (24) with osteosarcoma who presented to our clinic between March 2005 and June 2006’ is more reliable than a study that simply states ‘we report a case series of 24 people with osteosarcoma.’   N/PN if   - Evidence of incomplete inclusion of participants e.g. limited datasets used for exposure or outcome datasets - Selection bias due to hospital-based sample, including patients who were more likely to get tested. |
|  | 3.3 | For **case-control studies:**  This question was interpreted as: *“Was selection of participants into the study based on participant characteristics (except the outcome) observed after the start of the exposure window being studied?"* |
|  | 3.3.0 | Additional question **for case-control studies**:  “Do controls constitute a representative sample from the source population from which the cases arose”? *[specified in C2 and D1]*  If Y/PY: proceed to question 3.3.1,  If NI/N/PN: Is the control selection method likely to impact results? If Y/PY: High risk of bias in box B, if N/PN: Some concerns in box B. |
|  | 3.4 | Y—if matched-cohort studies compared ‘ever’ exposed individuals to ‘never’ exposed individuals during the study period (that is, the selection of the control cohort is influenced by future non-exposure) |
| Domain 4 (post-exposure interventions) | 4.1 | Examples of post-exposure interventions:   - COVID-19 vaccination roll-out during the period of follow-up - Treatment for adverse events from vaccination or other treatment - Test positivity/ symptoms ‘clinical indicated’ leading to CT scan. |
| Domain 5 (missing data) | - | **‘All participants’** include all those baseline and outcomes collected.   - If the total ‘analyzed participants’ i.e. from which the effect estimate was based on had missing data, but there was reasonable justification for exclusion from the analysis**,** we did not penalise this. - Evidence of missingness included:   Outcome: Baseline numbers are different from patients with outcome data, with no clear reasonable explanation.  Footnotes under tables, indicating missing data.  In the text, authors explain missing data, loss to follow-up etc |
|  | 5.2 | For **case-control studies:**  “Were complete data on the exposure available for all, or nearly all, participants?” |
|  | 5.3 | For **self-controlled case series:**  Y/PY—If there is clear reporting of clinical information of the participants where relevant: AMI/stroke status, socioeconomic status, comorbidities, vaccination status (if applicable), results of diagnostic tests, etc. |
|  | 5.5 | For **case-control studies:**  “Were the reasons for missing data related to case or control status?” |
| Domain 6 (measurement of the outcome) | 6.1 | For **self-controlled case series:**  Y/PY—The condition was measured in a standard, reliable way for all participants included in the case series e.g. standardised method of outcome collection for all patients. |
|  | 6.2 | For prospective cohort studies:  Y/PY—No blinding, incomplete or broken blinding, and outcome likely to be influenced.  N/PN—Blinding, and unlikely that the blinding could have been broken.   - No blinding or incomplete blinding, but outcome unlikely to be influenced - If outcome assessment for AMI/stroke was done preferentially based on clinical indications of respiratory infection e.g. presence of symptoms. |
|  |  | For **self-controlled case series:**  “Was data collection on laboratory testing affected by knowledge of the outcome or risk of the outcome?”  Y/PY—anonymized linkage between exposure and outcome datasets  N/PN—evidence of bias in outcome data collection |
|  |  | For case-control studies:  “Was the definition of case status/control status applied without knowledge of exposure status?”  “Was data collection on exposure status unaffected by knowledge of the outcome or risk of the outcome?” |
| Domain 7 (selection of the reported result) | 7.1 | N/PN—pre-determined analysis plan for most studies. |
|  | 7.2 | Examples of multiple exposure measurements described in the methods or data collection, whereby for some exposure measures in which the effect estimate is reported preferentially  e.g. PCR + Serology + self-report  e.g. ICD-10 codes + symptoms |
|  | 7.4 -7.5 | Scrutiny based on reported effect estimates likely to be selected based on desirability of the magnitude from multiple analyses and analyses of different subgroups. |

**Supplementary Methods 5.** Preferred Reporting Items for Systematic Reviews and Meta-analyses (PRISMA) Checklist^298^

| **Section and Topic** | **Item No.** | **Checklist item** | **Location where item is reported** |
| --- | --- | --- | --- |
| **TITLE** | | |  |
| Title | 1 | Identify the report as a systematic review. | p. 1, lines 1-2 |
| **ABSTRACT** | | |  |
| Abstract | 2 | See the PRISMA 2020 for Abstracts checklist. | p. 2, lines 21-41 |
| **INTRODUCTION** | | |  |
| Rationale | 3 | Describe the rationale for the review in the context of existing knowledge. | p. 3, lines 43-79 |
| Objectives | 4 | Provide an explicit statement of the objective(s) or question(s) the review addresses. | p. 4, lines 80-88 |
| **METHODS** | | |  |
| Eligibility criteria | 5 | Specify the inclusion and exclusion criteria for the review and how studies were grouped for the syntheses. | p. 5, lines 106-124 |
| Information sources | 6 | Specify all databases, registers, websites, organisations, reference lists and other sources searched or consulted to identify studies. Specify the date when each source was last searched or consulted. | p. 5, lines 94-105  Supplementary Methods 2 |
| Search strategy | 7 | Present the full search strategies for all databases, registers and websites, including any filters and limits used. | Supplementary Methods 2 |
| Selection process | 8 | Specify the methods used to decide whether a study met the inclusion criteria of the review, including how many reviewers screened each record and each report retrieved, whether they worked independently, and if applicable, details of automation tools used in the process. | pp. 5-6  Supplementary Methods 3 |
| Data collection process | 9 | Specify the methods used to collect data from reports, including how many reviewers collected data from each report, whether they worked independently, any processes for obtaining or confirming data from study investigators, and if applicable, details of automation tools used in the process. | pp. 5-6, lines 125-141 |
| Data items | 10a | List and define all outcomes for which data were sought. Specify whether all results that were compatible with each outcome domain in each study were sought (e.g. for all measures, time points, analyses), and if not, the methods used to decide which results to collect. | p. 5, lines 112-114 |
|  | 10b | List and define all other variables for which data were sought (e.g. participant and intervention characteristics, funding sources). Describe any assumptions made about any missing or unclear information. | p. 5, lines 107-117  p. 6, lines 132-134  As per PECOST elements |
| Study risk of bias assessment | 11 | Specify the methods used to assess risk of bias in the included studies, including details of the tool(s) used, how many reviewers assessed each study and whether they worked independently, and if applicable, details of automation tools used in the process. | p. 6, lines 138-141  Supplementary Methods 4 |
| Effect measures | 12 | Specify for each outcome the effect measure(s) (e.g. risk ratio, mean difference) used in the synthesis or presentation of results. | p. 5, lines 118-120  p. 6, lines 143-145 |
| Synthesis methods | 13a | Describe the processes used to decide which studies were eligible for each synthesis (e.g. tabulating the study intervention characteristics and comparing against the planned groups for each synthesis (item #5)). | p. 6, lines 142-156 |
|  | 13b | Describe any methods required to prepare the data for presentation or synthesis, such as handling of missing summary statistics, or data conversions. | p. 6, lines 145-146 |
|  | 13c | Describe any methods used to tabulate or visually display results of individual studies and syntheses. | p. 6, lines 153-156 |
|  | 13d | Describe any methods used to synthesize results and provide a rationale for the choice(s). If meta-analysis was performed, describe the model(s), method(s) to identify the presence and extent of statistical heterogeneity, and software package(s) used. | p. 6, lines 142-156 |
|  | 13e | Describe any methods used to explore possible causes of heterogeneity among study results (e.g. subgroup analysis, meta-regression). | p. 6, lines 157-162 |
|  | 13f | Describe any sensitivity analyses conducted to assess robustness of the synthesized results. | p. 9, lines 239-241  p. 10, lines 281-283  Supplementary Tables 2-3 |
| Reporting bias assessment | 14 | Describe any methods used to assess risk of bias due to missing results in a synthesis (arising from reporting biases). | p. 6, lines 159-161 |
| Certainty assessment | 15 | Describe any methods used to assess certainty (or confidence) in the body of evidence for an outcome. | pp. 6-7, lines 163-169 |
| **RESULTS** | | |  |
| Study selection | 16a | Describe the results of the search and selection process, from the number of records identified in the search to the number of studies included in the review, ideally using a flow diagram. | p. 8, lines 174-181  Figure 1 |
|  | 16b | Cite studies that might appear to meet the inclusion criteria, but which were excluded, and explain why they were excluded. | Supplementary Table 1 |
| Study characteristics | 17 | Cite each included study and present its characteristics. | Table 1 |
| Risk of bias in studies | 18 | Present assessments of risk of bias for each included study. | p. 8, lines 201-207  Figure 2 |
| Results of individual studies | 19 | For all outcomes, present, for each study: (a) summary statistics for each group (where appropriate) and (b) an effect estimate and its precision (e.g. confidence/credible interval), ideally using structured tables or plots. | Figures 3-4 |
| Results of syntheses | 20a | For each synthesis, briefly summarise the characteristics and risk of bias among contributing studies. | pp. 8-11, lines 209-294 |
|  | 20b | Present results of all statistical syntheses conducted. If meta-analysis was done, present for each the summary estimate and its precision (e.g. confidence/credible interval) and measures of statistical heterogeneity. If comparing groups, describe the direction of the effect. | pp. 8-10, lines 209-283  Figures 3-4 |
|  | 20c | Present results of all investigations of possible causes of heterogeneity among study results. | p. 10, lines 279-283  Supplementary Figures 3-6 |
|  | 20d | Present results of all sensitivity analyses conducted to assess the robustness of the synthesized results. | p. 9, lines 239-241  p. 10, lines 281-283  Supplementary Tables 2-3 |
| Reporting biases | 21 | Present assessments of risk of bias due to missing results (arising from reporting biases) for each synthesis assessed. | Figure 2  Supplementary Figure 2 |
| Certainty of evidence | 22 | Present assessments of certainty (or confidence) in the body of evidence for each outcome assessed. | Table 2 |
| **DISCUSSION** | | |  |
| Discussion | 23a | Provide a general interpretation of the results in the context of other evidence. | pp. 12-13, lines 296-360 |
|  | 23b | Discuss any limitations of the evidence included in the review. | p. 13, lines 361-371 |
|  | 23c | Discuss any limitations of the review processes used. | pp. 13-14, lines 371-389 |
|  | 23d | Discuss implications of the results for practice, policy, and future research. | p. 12, lines 324-329  p. 13, lines 342-344  p. 13, lines 351-353 |
| **OTHER INFORMATION** | | |  |
| Registration and protocol | 24a | Provide registration information for the review, including register name and registration number, or state that the review was not registered. | p. 5, lines 90-93 |
|  | 24b | Indicate where the review protocol can be accessed, or state that a protocol was not prepared. | p. 5, lines 91-92 |
|  | 24c | Describe and explain any amendments to information provided at registration or in the protocol. | Supplementary Methods 1 |
| Support | 25 | Describe sources of financial or non-financial support for the review, and the role of the funders or sponsors in the review. | p. 16, lines 399-402 |
| Competing interests | 26 | Declare any competing interests of review authors. | p. 16, line 410-416 |
| Availability of data, code and other materials | 27 | Report which of the following are publicly available and where they can be found: template data collection forms; data extracted from included studies; data used for all analyses; analytic code; any other materials used in the review. | p. 16, lines 417-420 |

**Supplementary Methods 6.** Meta-Analysis of Observational Studies in Epidemiology (MOOSE) Checklist^299^

| **Reporting Criteria** | **Reported (Yes/No)** | **Reported on Page No.** |
| --- | --- | --- |
| **Reporting of background** |  |  |
| Problem definition | Yes | p. 3, lines 43-71 |
| Hypothesis statement | Yes | p. 3, lines 72-79 |
| Description of study outcome(s) | Yes | p. 5, lines 112-114  Supplementary Methods 3 |
| Type of exposure or intervention used | Yes | p. 5, lines 108-110  Supplementary Methods 3 |
| Type of study designs used | Yes | p. 5, lines 114-116  Supplementary Methods 3 |
| Study population | Yes | p. 5, lines 120-124 |
| **Reporting of search strategy** |  |  |
| Qualifications of searchers (eg, librarians and investigators) | Yes | Online submission system |
| Search strategy, including time period included in the synthesis and key words | Yes | Supplementary Methods 2 |
| Effort to include all available studies, including contact with authors | Yes | p. 5, lines 103-105  p. 6, lines 128-129 |
| Databases and registries searched | Yes | p. 5, lines 100-103 |
| Search software used, name and version, including special features used (eg, explosion) | Yes | Supplementary Methods 2 |
| Use of hand searching (eg, reference lists of obtained articles) | Yes | p. 5, lines 103-105 |
| List of citations located and those excluded, including justification | Yes | Table 1  Supplementary Table 1 |
| Method of addressing articles published in languages other than English | Yes | p. 5, lines 120-121 |
| Method of handling abstracts and unpublished studies | Yes | p. 5, lines 120-121  Supplementary Table 1 |
| Description of any contact with authors | Yes | p. 6, lines 128-129  Supplementary Table 1 |
| **Reporting of methods** |  |  |
| Description of relevance or appropriateness of studies assembled for assessing the hypothesis to be tested | Yes | p. 5, lines 107-117 |
| Rationale for the selection and coding of data (eg, sound clinical principles or convenience) | Yes | pp. 5-6, lines 118-156 |
| Documentation of how data were classified and coded (eg, multiple raters, blinding and interrater reliability) | Yes | pp. 5-6, lines 125-137 |
| Assessment of confounding (eg, comparability of cases and controls in studies where appropriate) | Yes | Supplementary Methods 4 |
| Assessment of study quality, including blinding of quality assessors, stratification or regression on possible predictors of study results | Yes | p. 6, lines 138-141  Supplementary Methods 4 |
| Assessment of heterogeneity | Yes | p. 6, lines 146-153 |
| Description of statistical methods (eg, complete description of fixed or random effects models, justification of whether the chosen models account for predictors of study results, dose-response models, or cumulative meta-analysis) in sufficient detail to be replicated | Yes | p. 6, lines 142-162 |
| Provision of appropriate tables and graphics | Yes | p. 6, lines 153-162 |
| **Reporting of results** |  |  |
| Graphic summarizing individual study estimates and overall estimate | Yes | Figures 3-4 |
| Table giving descriptive information for each study included | Yes | Table 1 |
| Results of sensitivity testing (eg, subgroup analysis) | Yes | Supplementary Figures 3-6  Supplementary Tables 2-3 |
| Indication of statistical uncertainty of findings | Yes | 95% confidence intervals in Figures 3-4 |
| **Reporting of discussion** |  |  |
| Quantitative assessment of bias (eg, publication bias) | No – funnel plot only (insufficient number of studies) | Supplementary Figure 2 |
| Justification for exclusion (eg, exclusion of non-English language citations) | Yes | p. 13, lines 361-389 |
| Assessment of quality of included studies | Yes | p. 8, lines 201-207  Figure 2 |
| **Reporting of conclusions** |  |  |
| Consideration of alternative explanations for observed results | Yes | pp. 12-13, lines 295-360 |
| Generalization of the conclusions (ie, appropriate for the data presented and within the domain of the literature review) | Yes | p. 15, lines 390-398 |
| Guidelines for future research | Yes | p. 12, lines 324-329  p. 13, lines 342-344  p. 13, lines 351-353  p. 15, lines 394-396 |
| Disclosure of funding source | Yes | p. 16, lines 399-402 |

##

**SUPPLEMENTARY REFERENCES**

1. Coles KA, Knuiman MW, Plant AJ, Riley TV, Smith DW, Divitini ML. A prospective study of infection and cardiovascular diseases: The Busselton Health Study. *Eur J Cardiovasc Prev Rehabil* 2003;**10**:278–282.

2. Chu P, Cadogan SL, Warren-Gash C. Antibodies to Human Herpesviruses and Rate of Incident Cardiovascular Events and All-Cause Mortality in the UK Biobank Infectious Disease Pilot Study. *Open Forum Infect Dis* 2022;**9**:ofac294.

3. Sealy-Jefferson S, Gillespie BW, Aiello AE, Haan MN, Morgenstern LB, Lisabeth LD. Antibody levels to persistent pathogens and incident stroke in mexican americans. *Circ Conf Epidemiol Prev Act Nutr Metab* 2012;**125**.

4. Ozturk A, Gunes M, Altinoz Aytar A, Ozturk CE, Ankarali H. Are some chronic infections probable risk factors for acute ischemic stroke? *Turk Klin J Med Sci* 2013;**33**:726–731.

5. Arcari CM, Gaydos CA, Nieto EJ, Krauss M, Nelson KE. Association between Chlamydia pneumoniae and acute myocardial infarction in young men in the United States military: The importance of timing of exposure measurement. *Clin Infect Dis* 2005;**40**:1123–1130.

6. Esteban-Hernandez J, San Roman Montero J, Gil R, Anegon M, Gil A. Association between herpetic burden and chronic ischemic heart disease: Matched case-control study. [Spanish]. *Med Clin (Barc)* 2011;**137**:157–160.

7. Kenina V, Zanda P, Andrejs M, Galina B. Association between increased carotid intima-media thickness and cytomegalovirus seropositivity in stroke patients. *Cerebrovasc Dis* 2010;**1**:35.

8. Romo N, Fito M, Guma M, Sala J, Garcia C, Ramos R, Muntasell A, Masia R, Bruguera J, Subirana I, Vila J, Groot E de, Elosua R, Marrugat J, Lopez-Botet M. Association of atherosclerosis with expression of the LILRB1 receptor by human NK and T-cells supports the infectious burden hypothesis. *Arterioscler Thromb Vasc Biol* 2011;**31**:2314–2321.

9. Guan XR, Yang W, Sun XJ, Wang LF, Ma BJ, Li HY, Zhou J. Association of influenza virus infection and inflammatory cytokines with acute myocardial infarction. *Inflamm Res* 2012;**61**:591–598.

10. Chen S, Pawelec G, Trompet S, Goldeck D, Mortensen LH, Slagboom PE, Christensen K, Gussekloo J, Kearney P, Buckley BM, Ford I, Jukema JW, Westendorp RGJ, Maier AB. Associations of Cytomegalovirus Infection With All-Cause and Cardiovascular Mortality in Multiple Observational Cohort Studies of Older Adults. *J Infect Dis* 2021;**223**:238–246.

11. Witherell HL, Smith KL, Friedman GD, Ley C, Thom DH, Orentreich N, Vogelman JH, Parsonnet J. C-reactive protein, Helicobacter pylori, Chlamydia pneumoniae, cytomegalovirus and risk for myocardial infarction. *Ann Epidemiol* 2003;**13**:170–177.

12. Kurkowska-Jastrzebska I, Karlinski MA, Blazejewska-Hyzorek B, Sarzynska-Dlugosz I, Filipiak KJ, Czlonkowska A. Carotid intima media thickness and blood biomarkers of atherosclerosis in patients after stroke or myocardial infarction. *Croat Med J* 2016;**57**:548–557.

13. Wang XM, Li Y, Huang C, Zhang RH, Guo HT, Chen H, Wang HC. Changes of serum soluble P-selectin and tumor necrotic factor-alpha in patients with CMV induced acute coronary syndrome. *Chin J Exp Clin Virol* 2005;**19**:149–151.

14. Milovancev A, Petrovic M, Popadic V, Miljkovic T, Klasnja S, Djuran P, Ilic A, Kovacevic M, Milosavljevic AS, Brajkovic M, Crnokrak B, Memon L, Milojevic A, Todorovic Z, Cankovic M, Sarkanovic ML, Bjelic S, Tadic S, Redzek A, Zdravkovic M. Characteristics and Outcomes of Patients with Acute Coronary Syndrome and COVID-19. *J Clin Med* 2022;**11**.

15. Ammann P, Marschall S, Kraus M, Schmid L, Angehrn W, Krapf R, Rickli H. Characteristics and prognosis of myocardial infarction in patients with normal coronary arteries. *Chest* 2000;**117**:333–338.

16. Fagerberg B, Gnarpe J, Gnarpe H, Agewall S, Wikstrand J. Chlamydia pneumoniae but not cytomegalovirus antibodies are associated with future risk of stroke and cardiovascular disease: A prospective study in middle-aged to elderly men with treated hypertension. *Stroke* 1999;**30**:299–305.

17. Siscovick DS, Schwartz SM, Corey L, Grayston JT, Ashley R, Wang SP, Psaty BM, Tracy RP, Kuller LH, Kronmal RA. Chlamydia pneumoniae, herpes simplex virus type 1, and cytomegalovirus and incident myocardial infarction and coronary heart disease death in older adults -: The Cardiovascular Health Study. *Circulation* 2000;**102**:2335–2340.

18. Danesh J, Wong Y, Ward M, Muir J. Chronic infection with Helicobacter pylori, Chlamydia pneumoniae, or cytomegalovirus: Population based study of coronary heart disease. *Heart* 1999;**81**:245–247.

19. Padmavati S, Gupta U, Agarwal HK. Chronic infections & coronary artery disease with special reference to Chalmydia pneumoniae. *Indian J Med Res* 2012;**135**:228–232.

20. Mohsenizadeh SA, Alidoosti M, Jalali A, Tofighi S, Salarifar M, Poorhosseini H, Jenab Y, Ahmadian T. Comparison of Angiographic and Clinical Outcomes after Primary Percutaneous Coronary Intervention for ST-elevation Myocardial Infarction between Patients with and Without Concomitant COVID-19 Infection. *Crit Pathw Cardiol* 2022;**21**:141–146.

21. Gonzalez-Quijada S, Alamo-Martinez de Lagos M del, Alvarez-Llabres M, Perez-Gonzalez L. Cytomegalovirus antibody levels and mortality among hospitalised elderly patients. *Ann Med* 2020;**52**:497–505.

22. Sagedal S, Rollag H, Hartmann A. Cytomegalovirus infection in renal transplant recipients is associated with impaired survival irrespective of expected mortality risk. *Clin Transplant* 2007;**21**:309–313.

23. Savva GM, Pachnio A, Kaul B, Morgan K, Huppert FA, Brayne C, Moss PAH. Cytomegalovirus infection is associated with increased mortality in the older population. *Aging Cell* 2013;**12**:381–387.

24. Guan Y, Zhang M, Yuan H. Cytomegalovirus infection, tumor necrosis factor, endothelin and coronary heart disease. [Chinese]. *Chin J Cardiol* 1999;**27**:187–188.

25. Strachan DP, Carrington D, Mendall MA, Butland BK, Sweetnam PM, Elwood PC. Cytomegalovirus seropositivity and incident ischaemic heart disease in the Caerphilly prospective heart disease study. *Heart* 1999;**81**:248–251.

26. Karangizi AHK, Chanouzas D, Fenton A, Moss P, Cockwell P, Ferro CJ, Harper L. Cytomegalovirus seropositivity is independently associated with cardiovascular disease in non-dialysis dependent chronic kidney disease. *QJM* 2020;**113**:253–257.

27. Nikitskaya E, Lebedeva A, Ivanova O, Maryukhnich E, Shpektor A, Grivel JC, Margolis L, Vasilieva E. Cytomegalovirus-Productive Infection Is Associated With Acute Coronary Syndrome. *J Am Heart Assoc* 2016;**5**:20.

28. Yi L, Wang DX, Feng ZJ. Detection of human cytomegalovirus in atherosclerotic carotid arteries in humans. *J Formos Med Assoc* 2008;**107**:774–781.

29. Cheng HY, Fung E, Choi KC, Zou HJ, Chair SY. Early risk of acute myocardial infarction following hospitalization for severe influenza infection in the middle-aged population of Hong Kong. *PloS One* 2022;**17**:e0272661–e0272661.

30. Gianella S, Moser C, Vitomirov A, McKhann A, Layman L, Scott B, Lada S, Bosch R, Hoenigl M, Lurain N, Landay A, Lederman MM, Hunt PW, Smith DM. EBV and CMV levels in blood are associated with Non-AIDS Events during ART. *Top Antivir Med* 2019;**27**:90s.

31. Choussat R, Montalescot G, Collet JP, Jardel C, Ankri A, Fillet AM, Raymond J, Bastard JP, Drobinski G, Orfila J, Agut H, Thomas D. Effect of prior exposure to Chlamydia pneumoniae, Helicobacter pylori, or cytomegalovirus on the degree of inflammation and one-year prognosis of patients with unstable angina pectoris or non-Q-wave acute myocardial infarction. *Am J Cardiol* 2000;**86**:379–384.

32. Heltai K, Kis Z, Burian K, Endresz V, Veres A, Ludwig E, Gonczol E, Valyi-Nagy I. Elevated antibody levels against Chlamydia pneumoniae, human HSP60 and mycobacterial HSP65 are independent risk factors in myocardial infarction and ischaemic heart disease. *Atherosclerosis* 2004;**173**:339–346.

33. Zhang C, Huang P, Zhang P, Zhou M, Gao Y, Zhang Y, Guo Y, Lv L, Xu T. Elevated Serum Human Cytomegalovirus IgM Levels in the Acute Phase of Ischemic Stroke are Associated with Increased Risk of Death and Major Disability. *Curr Neurovasc Res* 2018;**15**:305–311.

34. Bennermo M, Nordin M, Lundman P, Boqvist S, Held C, Samnegard A, Ericsson CG, Silveira A, Hamsten A, Nastase MM, Tornvall P. Genetic and environmental influences on the plasma interleukin-6 concentration in patients with a recent myocardial infarction: A case-control study. *J Interferon Cytokine Res* 2011;**31**:259–264.

35. Santana MF, Frank CHM, Almeida TVR, Jeronimo CMP, Araujo Pinto RA de, Martins YF, Farias MEL de, Dutra BG, Brito-Sousa JD, Baia-da-Silva DC, Xavier MS, Lacerda MVG, Almeida Val FF, Monteiro GC, Sampaio VS, Monteiro WM, Ferreira LCL. Hemorrhagic and thrombotic manifestations in the central nervous system in COVID-19: A large observational study in the Brazilian Amazon with a complete autopsy series. *PLoS ONE* 2021;**16**:e0255950.

36. Abolbashari S, Abad ESS, Aryan E, Dehghani T, Moohebati M, Mobarhan MG, Meshkat Z, Meshkat M, Chechaklou AH, Khoshakhlagh M, Gholoubi A. Herpes Simplex Infection in Cardiovascular Disease (CVD) Patients: A Seroepidemiological Study. *J Kermanshah Univ Med Sci* 2023;**27**.

37. Fullerton HJ, Elkind MS, Glaser CA, Hills NK, Luna J, Sear K, Wintermark M, DeVeber GA. Herpes viruses in childhood arterial ischemic stroke: Interim results of the VIPS study (Abstract 38). *Stroke Conference*. San Diego: American Stroke Association; 2014.

38. Schlitt A, Blankenberg S, Weise K, Gartner BC, Mehrer T, Peetz D, Meyer J, Darius H, Rupprecht HJ. Herpesvirus DNA (Epstein-Barr virus, herpes simplex virus, cytomegalovirus) in circulating monocytes of patients with coronary artery disease. *Acta Cardiol* 2005;**60**:605–610.

39. Elkind MS, Hills NK, Glaser CA, Lo WD, Amlie-Lefond C, Dlamini N, Kneen R, Hod EA, Wintermark M, deVeber GA, Fullerton HJ. Herpesvirus Infections and Childhood Arterial Ischemic Stroke: Results of the VIPS Study. *Circulation* 2016;**133**:732–741.

40. Stockdale L, Nash S, Nalwoga A, Painter H, Asiki G, Fletcher H, Newton R. Human cytomegalovirus epidemiology and relationship to tuberculosis and cardiovascular disease risk factors in a rural Ugandan cohort. *PLoS ONE* 2018;**13**.

41. Zheng L, Sun Z, Zhang X, Jing K, Li J, Hu D, Sun Y. Human cytomegalovirus increases the risk of future hemorrhagic but not ischemic stroke - A nested case-control study. *Circ J* 2016;**80**:2235–2239.

42. Zhen J, Zeng M, Zheng X, Qiu H, Cheung BMY, Xu A, Wu J, Li C. Human cytomegalovirus infection is associated with stroke in women: the US National Health and Nutrition Examination Survey 1999-2004. *Postgrad Med J* 2022;**98**:172–176.

43. Kubrich M, Petrakopoulou P, Kofler S, Nickel T, Kaczmarek I, Meiser BM, Reichart B, Scheidt W von, Weis M. Impact of coronary endothelial dysfunction on adverse long-term outcome after heart transplantation. *Transplantation* 2008;**85**:1580–1587.

44. Nanavaty D, Sinha R, Kaul D, Sanghvi A, Kumar V, Vachhani B, Singh S, Devarakonda P, Reddy S, Verghese D. Impact of COVID-19 on Acute Myocardial Infarction: A National Inpatient Sample Analysis. *Curr Probl Cardiol* 2023;**49**:102030–102030.

45. Xu J, Yalkun G, Wang M, Wang A, Wangqin R, Zhang X, Chen Z, Mo J, Meng X, Li H, Li Z, Wang Y. Impact of Infection on the Risk of Recurrent Stroke Among Patients With Acute Ischemic Stroke. *Stroke* 2020;**51**:2395–2403.

46. Tarnacka B, Gromadzka G, Czlonkowska A. Increased circulating immune complexes in acute stroke: the triggering role of Chlamydia pneumoniae and cytomegalovirus. *Stroke* 2002;**33**:936–940.

47. Paganini-Hill A, Lozano E, Fischberg G, Barreto MP, Rajamani K, Ameriso SF, Heseltine PNR, Fisher M. Infection and risk of ischemic stroke: Differences among stroke subtypes. *Stroke* 2003;**34**:452–457.

48. Elkind MS, Luna JM, Moon YP, Boden-Albala B, Liu KM, Spitalnik S, Rundek T, Sacco RL, Paik MC. Infectious burden and carotid plaque thickness: the northern Manhattan study. *Stroke* 2010;**41**:e117-22.

49. Elkind MS, Ramakrishnan P, Moon YP, Boden-Albala B, Liu KM, Spitalnik SL, Rundek T, Sacco RL, Paik MC. Infectious burden and risk of stroke: the northern Manhattan study. *Arch Neurol* 2010;**67**:33–38.

50. Wolf SC, Brehm BR, Mayer O, Jurgens S, Schultze G, Risler T. Infectious risk factors for atherosclerotic vascular disease in hemodialysis patients - Chlamydia pneumoniae but not Helicobacter pylori or cytomegalovirus is associated with increased C-reactive protein. *Ren Fail* 2004;**26**:279–287.

51. Tripathi B, Kumar V, Kalra A, Gupta T, Sawant AC, Sharma P, Arora S, Panhwar MS, Gopalan R, Deshmukh A, Pershad A, Gulati M, Bhatt DL. Influence of Influenza Infection on In-Hospital Acute Myocardial Infarction Outcomes. *Am J Cardiol* 2020;**130**:7–14.

52. Warren-Gash C, Hayward A, Hemingway H, Denaxas S, Thomas SL, Timmis A, Whitaker H, Smeeth L. Influenza Infection and Risk of Acute Myocardial Infarction in England and Wales: A CALIBER Self-Controlled Case Series Study. *J Infect Dis* 2012;**206**:1652–1659.

53. Cowan L, Lutsey PL, Pankow JS, Matsushita K, Ishigami J, Lakshminarayan K. Inpatient and Outpatient Infection as a Trigger of Cardiovascular Disease: The ARIC Study. *J Am Heart Assoc* 2018;**7**:e009683.

54. Sheth AR, Grewal US, Patel HP, Thotamgari SR, Patel S, Desai R, Thakkar S, Papayannis A. Inpatient cardiovascular outcomes in patients with cancer affected by viral influenza infection. *Postgrad Med J* 2022;**99**:701–707.

55. Januel E, Bottin L, Yger M, Leger A, Crozier S, Baronnet F, Deltour S, Delorme S, Capron J, Borden A, Marro B, Clarencon F, Sourour NA, Samson Y, Rosso C, Alamowitch S. Ischaemic strokes associated with COVID-19: Is there a specific pattern? *J Neurol Neurosurg Psychiatry* 2021;**92**:452–454.

56. Lee S, Berg N van den, Castley A, Divitini M, Knuiman M, Price P, Nolan D, Sanfilippo F, Dwivedi G. Long-Term Associations between Human Cytomegalovirus Antibody Levels with All-Cause Mortality and Cardiovascular Outcomes in an Australian Community-Based Cohort. *Viruses* 2022;**14**:29.

57. Xie Y, Xu E, Bowe B, Al-Aly Z. Long-term cardiovascular outcomes of COVID-19. *Nat Med* 2022;**28**:583–590.

58. Lim JT, Liang En W, Tay AT, Pang D, Chiew CJ, Ong B, Lye DCB, Tan KB. Long-term Cardiovascular, Cerebrovascular, and Other Thrombotic Complications in COVID-19 Survivors: A Retrospective Cohort Study. *Clin Infect Dis* 2023;**78**:70–79.

59. Wan EYF, Mathur S, Zhang R, Lam AHY, Wang B, Yan VKC, Chui CSL, Li X, Wong CKH, Lai FTT, Cheung CL, Chan EWY, Tan KCB, Wong ICK. Long-term effects of coronavirus disease 2019 on diabetes complications and mortality in people with diabetes: Two cohorts in the UK and Hong Kong. *Diabetes Obes Metab* 2023;**25**:3807–3816.

60. Pesonen E, Hallman M, Sarna S, Andsberg E, Haataja R, Meri S, Persson K, Puolakkainen M, Ohlin H, Truedsson L. Mannose-binding lectin as a risk factor for acute coronary syndromes. *Ann Med* 2009;**41**:591–598.

61. Bularga A, Wereski R, Taggart C, Lowry M, Singh T, Lee KK, Anand A, Shah ASV, Ross DA, Perry MR, Dweck MR, Newby DE, Chapman AR, Mills NL. Mechanisms of myocardial injury and clinical outcomes in patients hospitalised with suspected COVID-19. *Eur Heart J* 2021;**42**:1154.

62. Li HZ, Wang Q, Zhang YY, Wang JD, Wu HJ, Zhang MG, Li JC, Liu ZJ. Onset of Coronary Heart Disease is Associated with HCMV Infection and Increased CD14 (+)CD16 (+) Monocytes in a Population of Weifang, China. *Biomed Env Sci* 2020;**33**:573–582.

63. De Backer J, Mak R, De Bacquer D, Van Renterghem L, Verbraekel E, Kornitzer M, De Backer G. Parameters of inflammation and infection in a community based case-control study of coronary heart disease. *Atherosclerosis* 2002;**160**:457–463.

64. Jennifer K, Shirley SBD, Avi P, Daniella RC, Naama SS, Anat EZ, Miri MR. Post-acute sequelae of COVID-19 infection. *Prev Med Rep* 2022;**31**:102097–102097.

65. Nagaraja D, Christopher R, Tripathi M, Kumar MV, Valli ER, Patil SA. Preceding infection as a risk factor of stroke in the young. *J Assoc Physicians India* 1999;**47**:673–675.

66. Gianella S, Moser C, Vitomirov A, McKhann A, Layman L, Scott B, Caballero G, Lada S, Bosch RJ, Hoenigl M, Lurain N, Landay A, Lederman MM, Hunt PW, Smith D. Presence of asymptomatic cytomegalovirus and Epstein--Barr virus DNA in blood of persons with HIV starting antiretroviral therapy is associated with non-AIDS clinical events. *AIDS* 2020;**34**:849–857.

67. Neumann FJ, Kastrati A, Miethke T, Pogatsa-Murray G, Seyfarth M, Schomig A. Previous cytomegalovirus infection and risk of coronary thrombotic events after stent placement. *Circulation* 2000;**101**:11–13.

68. Smieja M, Cronin L, Levine M, Goldsmith CH, Yusuf S, Mahony JB. Previous exposure to Chlamydia pneumoniae, Helicobacter pylori and other infections in Canadian patients with ischemic heart disease. *Can J Cardiol* 2001;**17**:270–276.

69. Lebedeva A, Maryukhnich E, Grivel JC, Vasilieva E, Margolis L, Shpektor A. Productive Cytomegalovirus Infection Is Associated With Impaired Endothelial Function in ST-Elevation Myocardial Infarction. *Am J Med* 2020;**133**:133–142.

70. Zhu JH, Nieto FJ, Horne BD, Anderson JL, Muhlestein JB, Epstein SE. Prospective study of pathogen burden and risk of myocardial infarction or death. *Circulation* 2001;**103**:45–51.

71. Kwon HS, Kwo JS, Lee D, Jeong DE, Lee JS, Kim SH, Kwon SU. Reactivation of varicella-zoster virus in reversible cerebral vasoconstriction syndrome: a proof-of-concept study. *Future Virol* 2021;**16**:259–264.

72. Grau AJ, Buggle F, Heindl S, Steichen-Wiehn C, Banerjee T, Maiwald M, Rohlfs M, Suhr H, Fiehn W, Becher H, Hacke W. Recent Infection as a Risk Factor for Cerebrovascular Ischemia. *Stroke* 1995;**26**:373–379.

73. Zurru MC, Alonzo C, Brescacin L, Romano M, Camera L, Waisman G, Cristiano E, Ovbiagele B. Recent respiratory infection predicts atherothrombotic stroke: case-control study in a Buenos Aires healthcare system. *Stroke* 2009;**40**:1986–1990.

74. Katsoularis I, Fonseca-Rodríguez O, Farrington P, Lindmark K, Connolly AF. Risk of acute myocardial infarction and ischaemic stroke following COVID-19 in Sweden: a self-controlled case series and matched cohort study. *Lancet Lond Engl* 2021;**398**:599–607.

75. Davidson J, Banerjee A, Smeeth L, McDonald H, Grint D, Herrett E, Forbes H, Pebody R, Warren-Gash C. Risk of acute respiratory infection and acute cardiovascular events following acute respiratory infection among adults with increased cardiovascular risk in England between 2008 and 2018: a retrospective, population-based cohort study. *Lancet Digit Health* 2021;**3**:e773–e783.

76. Velasquez Garcia HA, Wong S, Jeong D, Binka M, Naveed Z, Wilton J, Hawkins NM, Janjua NZ. Risk of Major Adverse Cardiovascular Events After SARS-CoV-2 Infection in British Columbia: A Population-Based Study. *Am J Med* 2024;**24**:24.

77. Smilowitz NR, Subashchandran V, Newman JD, Barfield ME, Maldonado TS, Brosnahan SB, Yuriditsky E, Horowitz JM, Shah B, Reynolds HR, Hochman JS, Berger JS. Risk of Thrombotic Events after Respiratory Infection Requiring Hospitalization. *Circ Conf Am Heart Assoc Sci Sess* 2020;**142**:A13288.

78. Haider M, Rizvi M, Malik A, Azam M, Rabbani MU. Role of CMV & Interferon-γ in Myocardial infarction, Angina and Hypertension. *Bangladesh J Med Sci* 2015;**14**:339–345.

79. Guech-Ongey M, Brenner H, Twardella D, Hahmann H, Rothenbacher D. Role of cytomegalovirus sero-status in the development of secondary cardiovascular events in patients with coronary heart disease under special consideration of diabetes. *Int J Cardiol* 2006;**111**:98–103.

80. Al-Ghamdi A. Role of herpes simplex virus-1, cytomegalovirus and Epstein-Barr virus in atherosclerosis. *Pak J Pharm Sci* 2012;**25**:89–97.

81. Pesonen E, Tiirola T, Andsberg E, Jauhiainen M, Paldanius M, Persson K, Saikku P, Sarna S, Ohlin H, Leinonen M. Serum chlamydial lipopolysaccharide as a prognostic factor for a new cardiovascular event. *Heart Lung J Crit Care* 2009;**38**:176–181.

82. Kamtchum-Tatuene J, Al-Bayati Z, Mwandumba HC, Solomon T, Christmas SE, Benjamin LA. Serum concentration of anti-Cytomegalovirus IgG and ischaemic stroke in patients with advanced HIV infection in Malawi. *PLoS ONE* 2018;**13**:e0208040.

83. Kervinen H, Tenkanen L, Palosuo T, Roivainen M, Manninen V, Manttari M. Serum iron, infection and inflammation; effects on coronary risk. *Scand Cardiovasc J* 2004;**38**:345–348.

84. Boas H, Tapia G, Hagberg G, Ihle-Hansen H, Berild JD, Selmer R, Karlstad O, Gulseth HL, Ariansen I. Stroke After SARS-CoV-2 mRNA Vaccine: A Nationwide Registry Study. *Stroke* 2023;**54**:E190–E193.

85. Li ML, Li ZZ, Li SP, He JY, Wang YX. Stroke in progression, infection of herpes virus and neurologic impairment inhibited with antivirus druggery. *Chin J Clin Rehabil* 2005;**9**:158–161.

86. Topcuoglu MA, Pektezel MY, Oge DD, Bulut Yuksel ND, Ayvacioglu C, Demirel E, Balci S, Arat A, Akinci SB, Arsava EM. Stroke Mechanism in COVID-19 Infection: A Prospective Case-Control Study. *J Stroke Cerebrovasc Dis* 2021;**30**:105919.

87. Ayati A, Hosseini K, Hadizadeh A, Jalali A, Lotfi-Tokaldany M, Milan N, Bagheri J, Ahmadi Tafti SH. Surgical coronary revascularization in patients with COVID-19; complications and outcomes: A retrospective cohort study. *Health Sci Rep* 2022;**5**:e751.

88. Jafarzadeh A, Nemati M, Tahmasbi M, Ahmadi P, Rezayati MT, Sayadi AR. The association between infection burden in Iranian patients with acute myocardial infarction and unstable angina. *Acta Medica Indones* 2011;**43**:105–111.

89. Haider AW, Wilson PWF, Larson MG, Evans JC, Michelson EL, Wolf PA, O’Donnell CJ, Levy D. The association of seropositivity to Helicobacter Pylori, Chlamydia Pneumoniae, and cytomegalovirus with risk of cardiovascular disease: A prospective study. *J Am Coll Cardiol* 2002;**40**:1408–1413.

90. Olga Blagova OV, Pavlenko EV, Varionchik NV, Nedostup AV, Kogan EA, Donnikov AE, Kadochnikova VV. The clinical, morphological diagnosis and prognostic value of the myocarditis in the left ventricular noncompation syndrome. *Eur J Heart Fail* 2018;**20**:313–314.

91. Zakliczynski M, Krynicka-Mazurek A, Pyka L, Trybunia D, Nadziakiewicz P, Przybylski R, Zembala M. The Effect of Epstein-Barr Virus Infection on Medium-Term Survival After Orthotopic Heart Transplantation. *Transplant Proc* 2007;**39**:2862–2865.

92. Amiri HA, Razavi AS, Tabrizi N, Cheraghmakani H, Baghbanian SM, Sedaghat-Chaijan M, Zarvani A, Ghazaeian M, Hosseinnataj A. The Effects of COVID-19 on Patients with Acute Ischemic and Hemorrhagic Stroke. *J Stroke Cerebrovasc Dis* 2022;**31**:106512.

93. Osman NM, Sayed NM, Abdel-Rahman SM, Hamza SA, Abd al aziz AA. The impact of cytomegalovirus infection on mechanically ventilated patients in the respiratory and geriatric intensive care units. *Egypt J Chest Dis Tuberc* 2014;**63**:239–245.

94. Zakliczynski M, Krynicka-Mazurek A, Pyka L, Trybunia D, Nadziakiewicz P, Przybylski R, Zembala M. The Influence of Cytomegalovirus Infection, Confirmed by pp65 Antigen Presence, on the Development of Cardiac Allograft Vasculopathy. *Transplant Proc* 2007;**39**:2866–2869.

95. Izadi M, Zamani MM, Sabetkish N, Abolhassani H, Saadat SH, Taheri S, Dabiri H. The probable role of cytomegalovirus in acute myocardial infarction. *Jundishapur J Microbiol* 2014;**7**:e9253.

96. Grakova E, Shilov SN, Berezikova EN, Kopeva KV, Popova AA, Samsonova EN, Pankova IV, Teplyakov AT. The role of cytomegalovirus infection in ischemic heart failure progression. *Eur Heart J* 2021;**42**:862.

97. Awadalla H, Eldemerdash S, El Din ME, El Naga HA. The role of infection in the development of acute coronary syndrome. *TAF Prev Med Bull* 2011;**10**:715–722.

98. Sair A, Akyildiz UO, Korkmazgil B, Kiylioglu N. The Value of Autoantibody and Viral Serologic Examinations in the Differential Diagnosis of Multiple Sclerosis and Stroke. *Turk J Neurol* 2019;**25**:208–213.

99. Ruane L, Buckley T, Hoo SYS, Hansen PS, McCormack C, Shaw E, Fethney J, Tofler GH. Triggering of acute myocardial infarction by respiratory infection. *Intern Med J* 2017;**47**:522–529.

100. Davidson JA, Banerjee A, Smeeth L, McDonald HI, Grint D, Herrett E, Forbes H, Pebody R, Warren-Gash C. Underlying cardiovascular risk and major adverse cardiovascular events after acute respiratory infection: a population-based cohort study of over 4.2 million individuals in England, 2008-2018. *medRxiv* 2021.

101. Fiordelisi D, Poliseno M, De Gennaro N, Milano E, Santoro CR, Segala FV, Franco CF, Manco Cesari G, Frallonardo L, Guido G, Metrangolo G, Romita G, Di Gennaro F, Saracino A. Varicella-Zoster Virus Reactivation and Increased Vascular Risk in People Living with HIV: Data from a Retrospective Cohort Study. *Viruses* 2023;**15**:2217.

102. Mattila KJ. Viral and bacterial infections in patients with acute myocardial infarction. *J Intern Med* 1989;**225**:293–296.

103. Pearce BD, Bracher A, Jones JL, Kruszon-Moran D. Viral and parasitic pathogen burden and the association with stroke in a population-based cohort. *Int J Stroke* 2018;**13**:481–495.

104. Pescetelli I, Marchetti M, Gomez Rosas P, Sanga E, Russo L, Moretti F, Pellegrini D, Fiocca L, Canova PA, Vassileva A, Gamba S, Verzeroli C, Grosu A, Guagliumi G, Falanga A. A prospective evaluation of COVID-19 associated coagulopathy with acute coronary syndrome (ACS). *Eur Heart J* 2021;**42**:1288.

105. MacIntyre R, Seale H. A randomised, controlled clinical trial of influenza vaccine in the prevention of recurrent ischaemic vascular events in patients with recent myocardial infarction, a transient ischaemic attack or with an ischaemic cerebrovascular event (stroke), aged between 40-64 years of age. *ANZCTR*. https://trialsearch.who.int/Trial2.aspx?TrialID=ACTRN12607000197437 (18 March 2024)

106. GlaxoSmithKline Biologicals. A randomized, observer-blind, active-controlled phase III study to demonstrate the superior efficacy of GSK Biologicals’ adjuvanted influenza candidate vaccine [GSK2186877A], administered intramuscularly in elderly aged 65 years or above, as compared to Fluarix^TM^. - FLU NG-006 PRI. *EUCTR*. https://trialsearch.who.int/Trial2.aspx?TrialID=EUCTR2008-000872-25-NL (18 March 2024)

107. Janssen-Cilag International NV. A study to investigate the effects of lumicitabine (JNJ-64041575) in hospitalized adults infected with human metapneumovirus. *EUCTR*. https://trialsearch.who.int/Trial2.aspx?TrialID=EUCTR2017-001696-22-PL (18 March 2024)

108. Richert ME, Andargie T, Balasubramanian S, Chan N, Kong H, Jang MK, Buescher PC, Agbor S. Cell-Free DNA to Identify Sources of Tissue Injury in COVID-19 Associated Complications. *Am J Respir Crit Care Med Conf Am Thorac Soc Int Conf ATS* 2023;**207**.

109. Alfadda AA, AlKhowaiter M, Alotaibi N, Alayed K, Alzahrani M, Binkhamis K, Siddiqui K, Youssef A, Altalhi H, Almaghlouth I, Alarifi M, Albanyan S, Alosaimi MF, Hasanato R, Isnani A, Dekhil H, Rafiullah M. Clinical and biochemical characteristics and outcomes of suspected COVID-19 hospitalized patients: RT-PCR swab positive and negative comparison. *J Infect Public Health* 2021;**14**:1623–1629.

110. Beccaria A, Svahn EJ, Bertamino M, Severino M, Pistorio A, Banov L, Molinari AC. Clinical and radiological outcome in a single center cohort of pediatric patients with focal cerebral arteriopathy (FCA). Safety of antithrombotic prophylaxis and therapy. *Res Pract Thromb Haemost* 2020;**4**:667–668.

111. Chen Q, Rui Q, Zhang W, Zheng X. Clinical Characteristics and Prognosis of Acute Ischemic Stroke with COVID-19 Infection. *Cerebrovasc Dis* 2023;**52**:180.

112. Cento V, Colagrossi L, Bossi I, Armenia D, Nava A, Piccinelli E, Maloberti A, Inglese E, Matarazzo E, Ruscio FDI, Paba P, Marcuccilli F, Perrone M, Chiricolo G, Alteri C, Scaglione F, Vismara C, Campisi DA, Fanti D, Romeo F, Andreoni M, Oliva F, Ceccherini-Silberstein F, Giannattasio C, Perno CF. cMV seroprevalence and coronary cMV-dNa detection in immunocompetent patients with heart diseases. *Minerva Med* 2023;**114**:289–299.

113. Sedghi A, Siepmann T, Barlinn J, Pallesen LP, Puetz V, Barlinn K. Compromised evidence-based acute stroke care during the secondwave of the COVID-19 pandemic. *Eur Stroke J* 2021;**6**:223.

114. Bagoly Z, Orban-Kalmandi R, Szegedi I, Arokszallasi T, Toth J, Csiba L. COVID-19 associated coagulopathy in acute ischemic stroke patients receiving intravenous thrombolysis. *Clin Chem Lab Med* 2021;**59**:eA66.

115. Ataniyazov M, Rakhimbaeva G, Gazieva S. COVID-19 associated stroke: Clinical forms and features of the disease. *World Congress of Neurology*. Rome, Italy: Journal of the Neurological Sciences; 2021. p119868.

116. Lewin D, Mulzer J, Marcus M, Nersesian G, Schonrath F, Falk V, Potapov E. COVID-19 in Patients with a Ventricular Assist Device. *Thorac Cardiovasc Surg Conf 51st Annu Meet Ger Soc Thorac Cardiovasc Surg DGTHG* 2022;**70**.

117. Priyadarshni S, Baillargeon J, Westra J, Kuo YF, Raji M. Covid-19 infection and incident myocarditis: A population-based propensity score-adjusted analysis. *Circ Conf Am Hear Assoc* 2021;**144**.

118. Patel BD, Chapman S, Visaria A, Hong S, Priyal M, Khodaverdi M, Wen S, Singh J, Ogungbe O, Neumann J, Mazimba S, Kimble W, Sokos G. Covid-19 Related Outcomes in Patients with Preexisting Cardiovascular Diseases, with or without Cancer. *J Am Coll Cardiol* 2022;**79**:2140.

119. Loczi L, Orban-Kalmandi R, Szegedi I, Arokszallasi T, Toth J, Olah L, Csiba L, Bagoly Z. COVID-19- associated hemostasis alterations and outcomes in acute ischemic stroke patients treated with intravenous thrombolysis. *Res Pract Thromb Haemost Conf* 2022;**6**.

120. Elakabawi K, Zahid B. CRT-100.96 Effect of COVID-19 Pandemic on Six-Month Mortality and Clinical Outcomes of Patients With ST-Elevation Myocardial Infarction. *JACC Cardiovasc Interv* 2023;**16(4 Supplement)**:S13.

121. Narrett JA, Assefa ED, Patel A, Mallawaarachchi I, Loomba JJ, Ratcliffe S, Sadan O, Monteith T, Worrall BB, Brown DE, Johnston KC, Southerland AM. Demographics, characteristics, and outcomes of stroke patients with concurrent SARS-CoV-2 infection from march 1, 2020 to February 28, 2020: An analysis from the n3c database. *Stroke Conf* 2022;**53**.

122. Li JM, Huang C, Zhou D. Detection of human herpersvirus-7 in 305 patients with intractable epilepsy. *Epilepsy Curr* 2013;**1**:467.

123. Sheikhi LE, O’Connor KF, Al-Salaimeh AA, Kennedy KA, Swafford KJ, Fraser JF, Pettigrew LC. Does COVID-19 worsen outcome in stroke thrombectomy? *Ann Neurol* 2021;**90**:S51–S52.

124. Roivainen M. Enteroviruses and myocardial infarction. *Am Heart J* 1999;**138**:S479-83.

125. Curtis SA, Billett HH, Starrels JL, Betancourt JL, Thomas M, Vattappally L, Crouch A, Minniti C. Excess All-Cause Mortality in 2020 for People Living with Sickle Cell Disease in New York City. *Blood* 2021;**138**:978.

126. Williams M, Chkheidze R, Powers R. Higher Frequency of Acute and Subacute Postmortem Neuropathologic Findings in Patients with COVID-19 Infection. *Mod Pathol* 2022;**35**:1203–1204.

127. Tallapally N, Devanandan P, Chowdary PR, Sekar C. Impact of COVID-19 on the clinical and angiographic profile in patients with coronary artery disease. *Pharm Educ* 2023;**23**:188.

128. Acton T, Subramanian A, Ettlinger J, Mamawala M, A GS, D WH, Arroliga ALEX. Impact of Social Distancing during the Sars-Cov-2 Pandemic on the Association between Viral Respiratory Illness and Cardiovascular-Related Events. *Chest* 2022;**162**:A285.

129. Degano IR, Tintore C, Camps-Vilaro A, Subirana I, Elosua R, Marrugat J. Increased incidence of arrhythmias, heart failure and thrombosis in COVID-19 patients after the first 3 months post-infection: a population-based cohort study. *Eur J Prev Cardiol* 2024;**31**:I51–I52.

130. Strambo D, De Marchis GM, Bonati L, Arnold M, Carrera E, Nedeltchev K, Kahles T, Cereda C, Kagi G, Luft A, Bolognese M, Salmen S, Sturzenegger R, Medlin F, Berger C, Schelosky L, Bonvin C, Mono ML, Rodic B, Schwegler G, Tarnutzer A, Peters N, Beyeler M, Engelter S, Fischer U, Michel P. Ischemic stroke in consecutive patients with COVID-19: A case-control study of clinical features, mechanisms and outcome in the swiss stroke registry. *Eur Stroke J* 2021;**6**:59–60.

131. Wei J, Wang W, Wang C, Wang S. Long-term cardiovascular outcomes in Covid-19 survivors among non-vaccinated population: a retrospective cohort study from the trinetx us collaborative networks. *Int J Rheum Dis* 2023;**26**:66.

132. Silva R, Ferreira M, Magalhaes J, Lant S, Gasque K, Abuquerque M, Braga M, Morais C, Salvi C. NeuroCOVID protocol and pilot results: A multicentre case-control study of acute stroke and SARS-CoV-2 infection. *Eur J Neurol* 2021;**28**:772.

133. McDonald M, Bhattacharjee M. Neuropathology in COVID-19 Autopsy Examinations - A Single Institutional Experience. *J Neuropathol Exp Neurol* 2021;**80**:587.

134. Makkar K, Sharma YP. Observational study in a tertiary care center in North India comparing demographic profile and outcome in patients with COVID-19 ACS and non COVID-19 ACS. *Indian Heart J* 2021;**73**:S10.

135. Patel B, Chapman SA, Neumann JT, Visaria A, Ogungbe O, Wen S, Khodaverdi M, Makwana P, Singh JA, Sokos G. Outcomes of patients with active cancers and pre-existing cardiovascular diseases infected with SARS-CoV-2. *Cardio-Oncol* 2023;**9**.

136. Luna JM, Fullerton HJ, Wintermark M, DeVeber G, Hills N, Muhammad K, Tokarz R, Lipkin WI, Elkind MS. Parvovirus B19 DNA prevalence is increased in pediatric stroke patients compared to controls: Pilot findings from the vascular effects of infection in pediatric stroke (VIPS) study. *Stroke Conf* 2014;**45**.

137. Kashem TS, Begum NAS, Nobi F, Arefin SZ, Rashid HU. Pos-529 Covid 19 Infection among Haemodialysis Patients of a Specialised Kidney Hospital in Bangladesh. *Kidney Int Rep* 2021;**6**:S230–S231.

138. Sirine B, hamouda M, salem M ben, handous I, salah M ben, ltaief A, aloui S, skhiri H. Pos-770 Impact of Cytomegalovirus Infection in the Long-Term Outcome of Renal Transplantation: A Single Center 20-Year Experience. *Kidney Int Rep* 2022;**7**:S332.

139. Kearney A, Hanzlik-Meech M, Mackay C, Evans L, Robins A, Simpson A, Abuzeid W. Prediction of Hospitalization and Icu Admission for Ontario Covid-19 Patients with Cardiac Comorbidities. *J Am Coll Cardiol* 2022;**79**:1843.

140. Sud M, Qiu F, Shah R, Lee DS, Nallamothu BK, Natarajan MK, Wijeysundera HC, Ko DT. Rate of COVID-19 Infection in Patients With ST-Segment Elevation Myocardial Infarction. *CJC Open* 2021;**3**:1214–1216.

141. Neidlinger NA, Pirsch JD, Odorico JS, Becker YT, Sollinger HW. Risk factors for death among 1,000 pancreas-kidney transplants at a single center. *Am J Transplant* 2010;**4**:113–114.

142. Mainous AG, Rooks BJ, Orlando FA. Risk of new hospitalization post-COVID-19 infection for non-covid-19 conditions. *J Am Board Fam Med* 2021;**34**:907–913.

143. Knight S, Miller RR, Bair TL, Horne BD, Lopansri BK, Anderson JL, Muhlestein JB, Carlquist JF. Risk of subsequent cardiovascular events following a respiratory viral infection. *Circ Conf Am Heart Assoc* 2014;**130**.

144. Long J, Giles J, Zhang L, Kang P, Baldassari L, Andruska K, Burnham CA, Wilen C, Ances B, Bucelli R. Sensitivity and specificity of CSF VZV antibody and pcr testing in suspected VZV vasculopathy. *Ann Neurol* 2017;**82**:S133.

145. Dahle DO, Asberg A, Hartmann A, Holdaas H, Jenssen TG, Dionisi M, Pasch A. Serum calcification propensity predicts mortality in kidney transplant recipients. *Nephrol Dial Transplant* 2015;**3**:iii36.

146. Perissinotti I, Schettini M, Del Guerra F, Fonseca B, Tinone G, Nitrini R, Conforto A. Stroke Etiologies in Patients with Covid-19. *Neuroepidemiology* 2022;**56**:63.

147. Subramanian A, McGraw M, Carlin J, Zolfaghari K, Ghamande S, White H. Stroke Following Positive Biomarker for Viral Respiratory Illness. *Chest* 2020;**158**:A348.

148. Levine S, Linzer R, Labins J, Kim J, Kimura A, Kiyatkin M. Surgical morbidity and mortality during the COVID-19 epidemic in new york city. *Crit Care Med* 2021;**49**:99.

149. Gharib I, Annie F, Rinehart S. TCT-551 The Effect of the COVID-19 Vaccines on Individuals Who Develop Incidences of Nontraumatic Intracranial Hemorrhages. *J Am Coll Cardiol* 2022;**80**:B227.

150. Islam AW, Reza AQM, Munwar S, Talukder SU, Tamzeed Ahmed TA, Rahman KA, Bhuiyan AH. TCTAP A-014 Pharmacoinvasive Therapy in Treating Acute STEMI Patients in COVID-19 Era-Thrombolysis by Tenecteplase (TNK) / Streptokinase (STK) Followed by Rescue PCI of the Culprit Vessel. *J Am Coll Cardiol* 2023;**81**:S9.

151. Ozdemir FN, Akgul A, Altunoglu A, Bilgic A, Arat Z, Haberal M. The Association Between Cytomegalovirus Infection and Atherosclerotic Events in Renal Transplant Recipients. *Transplant Proc* 2007;**39**:990–992.

152. Oleske D, Talapala S, Kukreja P, Facheris M, Dorsey R, Dey S, Isherwood A. The COVID-19 pandemic impact on advanced parkinson’s disease in the US. *Mov Disord* 2021;**36**:S77.

153. Kurkowska-Jastrzebska I, Blazejewska-Hyzorek B, Sarzynska-Dlugosz I, Karlinski M, Kiliszek M, Glowczynska R, Filipiak K, Czlonkowska A. The inflammatory serum markers in patients with a recent history of stroke and myocardial infarction. *Int J Stroke* 2015;**2**:244–245.

154. Aslan AN, Bilir YA, Selcuk T, Temizhan A, Akcay M. The management of acute coronary syndrome patients in COVID-19 pandemic. *Anatol J Cardiol* 2020;**24**:94.

155. Akinyemi A, Mangold K, Ernst L. The Prevalence of SARS-CoV-2 In Autopsy Tissue from Patients Dying Without Known COVID-19 Disease. *Mod Pathol* 2022;**35**:1.

156. Mercadé-Besora N, Li X, Kolde R, Trinh NT, Sanchez-Santos MT, Man WY, Roel E, Reyes C, Delmestri A, Nordeng HM, Uusküla A, Duarte-Salles T, Prats C, Prieto-Alhambra D, Jödicke AM, Català M. The role of COVID-19 vaccines in preventing post COVID-19 thromboembolic and cardiovascular complications: a multinational cohort study. 2023.

157. Fullerton HJ, Elkind MSV, Barkovich AJ, Glaser C, Glidden D, Hills NK, Leiva-Salinas C, Wintermark M, DeVeber GA. The Vascular Effects of Infection in Pediatric Stroke (VIPS) study. *J Child Neurol* 2011;**26**:1101–1110.

158. Fullerton HJ, Wintermark M, Hills NK, Amlie-Lefond C, Bernard TJ, Dowling MM, Friedman N, Jordan LC, Grose CF. Varicella Zoster Virus Infection in Children With Arterial Ischemic Stroke: The Chicken or the Egg? *Stroke Conf Am Stroke Assoc* 2024;**55**.

159. Immovilli P, Marchesi E, Terracciano C, Morelli N, Bazzurri V, Magnifico F, Zaino D, Terlizzi E, De Mitri P, Vollaro S, Mometto N, Guidetti D. A ‘Post-mortem’ of COVID-19-associated stroke: a case-control study. *J Stroke Cerebrovasc Dis* 2022;**31**.

160. Garcia-Lamberechts EJ, Miro O, Fragiel M, Llorens P, Jimenez S, Pinera P, Burillo-Putze G, Martin A, Martin-Sanchez FJ, Jacob J, Alquezar-Arbe A, Ejarque Martinez L, Rodriguez Miranda B, Ruiz Grinspan M, Dominguez MJ, Teigell Munoz FJ, Gayoso Martin S, Garcia Garcia A, Iglesias Vela M, Carbajosa V, Salido Mota M, Marchena Gonzalez MJ, Aguera Urbano C, Porta-Etessam J, Calvo E, Gonzalez del Castillo J. A case-control analysis of stroke in COVID-19 patients: Results of unusual manifestations of COVID-19-study 11. *Acad Emerg Med* 2021;**28**:1236–1250.

161. Dhamoon MS, Thaler A, Gururangan K, Kohli A, Sisniega D, Wheelwright D, Mensching C, Fifi JT, Fara MG, Jette N, Cohen E, Dave P, DiRisio AC, Goldstein J, Loebel EM, Mayman NA, Sharma A, Thomas DS, Vega Perez RD, Weingarten MR, Wen HH, Tuhrim S, Stein LK. Acute Cerebrovascular Events With COVID-19 Infection. *Stroke* 2021;**52**:48–56.

162. Ferlini M, Castini D, Ferrante G, Marenzi G, Montorfano M, Savonitto S, D’Urbano M, Lettieri C, Cuccia C, Marino M, Visconti LO, Carugo S. Acute Coronary Syndromes and SARS-CoV-2 Infection: Results From an Observational Multicenter Registry During the Second Pandemic Spread in Lombardy. *Front Cardiovasc Med* 2022;**9**:912815.

163. Kvernland A, Kumar A, Yaghi S, Raz E, Frontera J, Lewis A, Czeisler B, Kahn DE, Zhou T, Ishida K, Torres J, Riina HA, Shapiro M, Nossek E, Nelson PK, Tanweer O, Gordon D, Jain R, Dehkharghani S, Henninger N, Havenon A de, Grory BM, Lord A, Melmed K. Anticoagulation use and Hemorrhagic Stroke in SARS-CoV-2 Patients Treated at a New York Healthcare System. *Neurocrit Care* 2021;**34**:748–759.

164. Chughtai AA, Tan TC, Hitchen EM, Kunasekaran M, MacIntyre CR. Association of influenza infection and vaccination with cardiac biomarkers and left ventricular ejection fraction in patients with acute myocardial infarction. *IJC Heart Vasc* 2020;**31**:100648.

165. Havenon A de, Ney JP, Callaghan B, Hohmann S, Shippey E, Yaghi S, Anadani M, Majersik JJ. Characteristics and Outcomes Among US Patients Hospitalized for Ischemic Stroke Before vs During the COVID-19 Pandemic. *JAMA Netw Open* 2021;**4**:e2110314.

166. Briscoe M, Sykes R, Krystofiak T, Peck O, Mangion K, Berry C. Clinical significance of coronavirus disease 2019 in hospitalized patients with myocardial injury. *Clin Cardiol* 2021;**44**:332–339.

167. Sheriff F, Lavezo J, Floresca R, Chaudhury MR, Colina G, Regenhardt R, Gupta V, Rodriguez G, Maud A. Clinicopathologic Analysis of COVID-19 Associated Thrombi in the Setting of Large Vessel Occlusion: A Prospective Case-Control Study. *Stroke-Vasc Interv Neurol* 2023;**3**.

168. Gorgulu U, Bayindir H, Bektas H, Kayipmaz AE, San I. Coexistence of neurological diseases with Covid-19 pneumonia during the pandemic period. *J Clin Neurosci* 2021;**91**:237–242.

169. Arun BS, Makhale CN, Grant P, Khan N, Mitra P, Tribhuwan S, Sing A, Dhamdhere P. Comparative study of COVID 19 positive and COVID 19 negative STEMI (ST elevation myocardial infarction) during covid pandemic. *J Cardiovasc Dis Res* 2022;**13**:1524–1536.

170. Okuyan DY, Golen MK. Comparison of patients with acute ischemic stroke with and without covid-19. *Neurol Asia* 2021;**26**:449–458.

171. Quinn KL, Huang A, Bell CM, Detsky AS, Lapointe-Shaw L, Rosella LC, Urbach DR, Razak F, Verma AA. Complications Following Elective Major Noncardiac Surgery among Patients with Prior SARS-CoV-2 Infection. *JAMA Netw Open* 2022;**5**:e2247341.

172. Lawton MT, Alimohammadi E, Bagheri SR, Bostani A, Vaziri S, Karbasforoushan A, Mozaffari K, Bukani MB, Abdi A. Coronavirus disease 2019 (COVID-19) can predispose young to Intracerebral hemorrhage: a retrospective observational study. *BMC Neurol* 2021;**21**:83.

173. Henderson J, Britton Z, Hall C, Brown M, Man K, Abraham-Thomas N, Drumm B, D’Anna L, Kwan J, Brown Z, Malik A, Venter M, Halse O, Jamil S, Dixon L, Ali Sheikh A, Elmamoun S, Kalladka D, Banerjee S. COVID-19 infection and stroke in hospitalised patients: A retrospective analysis of incidence, stroke character, risk factors and mortality. *Eur Stroke J* 2021;**6**:290–291.

174. Zahid B, Kamal M, Said M, Salem M, Elakabawi K. Effect of COVID-19 pandemic on six-month mortality and clinical outcomes of patients with ST-elevation myocardial infarction. *Postepy W Kardiologii Interwencyjnej* 2022;**18**:228–236.

175. Pellegrini D, Fiocca L, Pescetelli I, Canova P, Vassileva A, Faggi L, Senni M, Guagliumi G. Effect of respiratory impairment on the outcomes of primary percutaneous coronary intervention in patients with ST-segment elevation myocardial infarction and Coronavirus disease-2019 (COVID-19). *Circ J* 2021;**85**:1701–1707.

176. Havenon A de, Yaghi S, Mistry EA, Delic A, Hohmann S, Shippey E, Stulberg E, Tirschwell D, Frontera JA, Petersen NH, Anadani M. Endovascular thrombectomy in acute ischemic stroke patients with COVID-19: prevalence, demographics, and outcomes. *J Neurointerventional Surg* 2020;**12**:1045–1048.

177. Roivainen M, Alfthan G, Jousilahti P, Kimpimaki M, Hovi T, Tuomilehto J. Enterovirus infections as a possible risk factor for myocardial infarction. *Circulation* 1998;**98**:2534–2537.

178. Al Qawasmeh M, Ahmed YB, Nsour OA, Qarqash AA, Al-Horani SS, Hazaimeh EA, Jbarah OF, Yassin A, Aldabbour B, Alhusban A, El-Salem K. Functional outcomes of COVID-19 patients with acute ischemic stroke: A prospective, observational, single-center study in North Jordan. *Med U S* 2022;**101**:e29834.

179. Wojcik M, Karpiak J, Zareba L, Przybylski A. High in-hospital mortality and prevalence of cardiogenic shock in patients with ST-segment elevation myocardial infarction and concomitant COVID-19. *Adv Interv Cardiol* 2023;**19**:22–30.

180. Gallego Y, Arias MP, Diaz AG, Freixa A, Vazquez D, Capdevila GM, Salvany S, Saldana AS, Fernandez ER, Pedro ES, Sanahuja J, Mingot CG, Requena LQ, Martinez AQ, Otin MP, Garcia FP. IMPACT OF COVID-19 IN ACUTE ACUTE ISCHEMIC STROKE IN THE VACCINATION ERA: Data from the Ilercovid project. *9th European Stroke Organisation Conference*. Munich, Germany: European Stroke Journal; 2023. p570.

181. Sangalli D, Martinelli-Boneschi F, Versino M, Colombo I, Ciccone A, Beretta S, Marcheselli S, Altavilla R, Roncoroni M, Beretta S, Lorusso L, Cavallini A, Prelle A, Guidetti D, La Gioia S, Santalucia P, Zanferrari C, Grampa G, D’Adda E, Peverelli L, Colombo A, Salmaggi A. Impact of SARS-CoV-2 infection on acute intracerebral haemorrhage in northern Italy. *J Neurol Sci* 2021;**426**:117479.

182. De Luca G, Debel N, Cercek M, Jensen LO, Vavlukis M, Calmac L, Johnson TW, Ferrer GR, Ganyukov V, Wojakowski W, Kinnaird T, Birgelen C von, Cottin Y, Ijsselmuiden A, Tuccillo B, Versaci F, Royaards KJ, Berg JMT, Laine M, Dirksen MT, Siviglia M, Casella G, Kala P, Gil JLD, Banning AP, Becerra V, De Simone C, Santucci A, Carrillo X, Scoccia A, Amoroso G, Hof AWJ van ’t, Kovarnik T, Tsigkas G, Mehilli J, Gabrielli G, Rios XF, Bakraceski N, Levesque S, Cirrincione G, Guiducci V, Kidawa M, Spedicato L, Marinucci L, Ludman P, Zilio F, Galasso G, Fabris E, Menichelli M, García-Touchard A, Manzo S, Caiazzo G, Moreu J, Forés JS, Donazzan L, Vignali L, Teles RC, Benit E, Agostoni P, Ojeda FB, Lehtola H, Camacho-Freiere S, Kraaijeveld AO, Antti Y, Boccalatte M, Deharo P, Martínez-Luengas IL, Scheller B, Varytimiadi E, Moreno R, Uccello G, Faurie B, Barrios AG, Milewski M, Bruwiere E, Smits PC, Wilbert B, Di Uccio FS, Parodi G, Kedhi E, Verdoia M. Impact of SARS-CoV-2 positivity on clinical outcome among STEMI patients undergoing mechanical reperfusion: Insights from the ISACS STEMI COVID 19 registry. *Atherosclerosis* 2021;**332**:48–54.

183. Hasibul Hasan ATM. In hospital mortality and short-term outcome of acute ischemic stroke patients contracting SARS CoV-2 infection: Experience from a dedicated stroke unit in Bangladesh. *World Congress of Neurology*. Rome, Italy: Journal of the Neurological Sciences; 2021. p429 Supplement.

184. Ciolli L, Righi V, Vandelli G, Giacobazzi L, Biagioli N, Marzullo D, Vandelli L, Rosafio F, Vinceti G, Maffei S, Picchetto L, Dell’Acqua ML, Borzi GM, Ricceri R, Bigliardi G, Meletti S. In-hospital and out-of-hospital stroke in patients with COVID-19: two different diseases? *Neurol Sci* 2022;**43**:2203–2210.

185. Kihira S, Schefflein J, Chung M, Mahmoudi K, Rigney B, Delman BN, Mocco J, Doshi A, Belani P. Incidental COVID-19 related lung apical findings on stroke CTA during the COVID-19 pandemic. *J Neurointerventional Surg* 2020;**12**:669–672.

186. Baştan B, Erdağ Turgeon E, Şanlı E, Bayar MD, Şişman AB, Atacan Yaşgüçlükal M, Küçükali CIS, Tüzün E, Günaydın S. Increased neuropil antibody prevalence in COVID-19 patients with acute ischemic stroke. *Neurol Res* 2023;**45**:988–993.

187. Matsushita K, Marchandot B, Carmona A, Curtiaud A, El Idrissi A, Trimaille A, Kibler M, Cardi T, Heger J, Hess S, Reydel A, Jesel L, Ohlmann P, Morel O. Increased susceptibility to SARS-CoV-2 infection in patients with reduced left ventricular ejection fraction. *ESC Heart Fail* 2021;**8**:380–389.

188. Wang J, Chen J. Infection with COVID-19 is a risk factor for poor prognosis in patients with intracranial hemorrhage: A prospective observational cohort study. *Medicine (Baltimore)* 2023;**102**.

189. Sripadma PV, Jain RS, Vyas A, Sharma B, Srivastava T, Murarka S, Saavaliya P, Agrawal J, Rao K. Isolated Acute Cerebrovascular Involvement in COVID-19 without Fever and Respiratory Symptoms: An Indian Perspective. *J Assoc Physicians India* 2021;**69**:45–50.

190. Sawczynska K, Wrona P, Kesek T, Wnuk M, Chrzan R, Homa T, Pulyk R, Jagiella J, Popiela T, Slowik A. Mechanical thrombectomy in COVID-19-associated ischaemic stroke: patient characteristics and outcomes in a single-centre study. *Neurol Neurochir Pol* 2022;**56**:163–170.

191. Ucles J, Cuesta E, Rigual R, Rodriguez-Pardo J, Ruiz-Ares G, Navia P, Fernandez-Prieto A, Alvarez-Muelas A, Lecinana MA de, Fuentes B. Neck CT angiography in acute stroke: An open window for fast detection of COVID-19 lung involvement? Applicability in telemedicine. *PLoS ONE Electron Resour* 2023;**18**:e0281955.

192. Gonzalez F, Caiza Zambrano F, Bala M, Saucedo M, Bandeo L, Leon Cejas L, Uribe Roca C, Bottaro F, Fernandez Pardal M, Reisin R, Bonardo P. One year after the pandemic in Argentina, clinical characteristics of patients. *Neurol Argent* 2022;**14**:85–91.

193. Kotecha T, Knight DS, Razvi Y, Kumar K, Vimalesvaran K, Thornton G, Patel R, Chacko L, Brown JT, Coyle C, Leith D, Shetye A, Ariff B, Bell R, Captur G, Coleman M, Goldring J, Gopalan D, Heightman M, Hillman T, Howard L, Jacobs M, Jeetley PS, Kanagaratnam P, Kon OM, Lamb LE, Manisty CH, Mathurdas P, Mayet J, Negus R, Patel N, Pierce I, Russell G, Wolff A, Xue H, Kellman P, Moon JC, Treibel TA, Cole GD, Fontana M. Patterns of myocardial injury in recovered troponin-positive COVID-19 patients assessed by cardiovascular magnetic resonance. *Eur Heart J* 2021;**42**:1866–1878.

194. Beslow LA, Linds AB, Fox CK, Kossorotoff M, Zuniga Zambrano YC, Hernandez-Chavez M, Hassanein SMA, Byrne S, Lim M, Maduaka N, Zafeiriou D, Dowling MM, Felling RJ, Rafay MF, Lehman LL, Noetzel MJ, Bernard TJ, Dlamini N. Pediatric Ischemic Stroke: An Infrequent Complication of SARS-CoV-2. *Ann Neurol* 2021;**89**:657–665.

195. Syrjanen J, Valtonen VV, Iivanainen M, Kaste M, Huttunen JK. Preceding infection as an important risk factor for ischaemic brain infarction in young and middle aged patients. *Br Med J Clin Res Ed* 1988;**296**:1156–1160.

196. Altschul DJ, Esenwa C, Haranhalli N, Unda SR, La Garza Ramos R de, Dardick J, Fernandez-Torres J, Toma A, Labovitz D, Cheng N, Lee SK, Brook A, Zampolin R. Predictors of mortality for patients with COVID-19 and large vessel occlusion. *Interv Neuroradiol* 2020;**26**:623–628.

197. Mortensen JK, Blauenfeldt RA, Hedegaard JN, Morberg Wejse C, Johnsen SP, Andersen G, Simonsen CZ. Prevalence and impact of SARS-CoV-2 infection among patients with acute ischaemic stroke: a nationwide register-based cohort study in Denmark. *BMJ Open* 2024;**14**:e081527.

198. Khedr EM, Abdelwarith A, Moussa GM, Saber M. Recombinant tissue plasminogen activator (rTPA) management for first onset acute ischemic stroke with covid -19 and non-covid -19 patients. *J Stroke Cerebrovasc Dis* 2023;**32**:107031.

199. Vielleux MJ, Swartwood S, Nguyen D, James KE, Barbeau B, Bonkowsky JL. SARS-CoV-2 Infection and Increased Risk for Pediatric Stroke. *Pediatr Neurol* 2023;**142**:89–94.

200. De Michele M, Lorenzano S, Piscopo P, Rivabene R, Crestini A, Chistolini A, Stefanini L, Pulcinelli FM, Berto I, Campagna R, Amisano P, Iacobucci M, Cirelli C, Falcou A, Nicolini E, Schiavo OG, Toni D. SARS-CoV-2 infection predicts larger infarct volume in patients with acute ischemic stroke. *Front Cardiovasc Med* 2022;**9**:1097229.

201. Cherenko TM, Turchyna NS, Heletiuk YL, Andriushkova NG, Kuzminska OV. SEASONAL FACTOR AND FREQUENCY OF DETECTION OF HERPES VIRUSES AND INFLUENZA VIRUS IN PATIENTS WITH ISCHEMIC STROKE. *Wiad Lek* 2021;**74**:923–928.

202. Anghel L, Tudurachi BS, Leonte A, Sascau RA, Zota IM, Bazyani A, Tinica G, Statescu C. The Challenge of High Coronary Thrombotic Events in Patients with ST-Segment Elevation Myocardial Infarction and COVID-19. *J Clin Med* 2022;**11**.

203. Kazirod-Wolski K, Zajac P, Zabojszcz M, Kolodziej A, Sielski J, Siudak Z. The Effect of COVID-19 on the Perioperative Course of Acute Coronary Syndrome in Poland: The Estimation of Perioperative Prognosis and Neural Network Analysis in 243,515 Cases from 2020 to 2021. *J Clin Med* 2022;**11**.

204. Takacs TT, Berki AJ, Bojti PP, Stang R, Fritz-Reunes PA, Schnekenberg L, Siepmann T, Pinter A, Szatmari S, Bereczki D, Gunda BB. The Impact of Sars-Cov-2 Infection on the Outcome of Acute Ischemic Stroke - a Retrospective Cohort Study. *Eur Stroke J* 2022;**7**:462.

205. Takacs TT, Berki AJ, Bojti PP, Stang R, Fritz-Reunes PA, Schnekenberg L, Siepmann T, Pinter A, Szatmari S, Bereczki D, Gunda B. The impact of SARS-CoV-2 infection on the outcome of acute ischemic stroke-A retrospective cohort study. *PLoS ONE* 2023;**18**:e0282045.

206. Chui CSL, Fan M, Wan EYF, Leung MTY, Cheung E, Yan VKC, Gao L, Ghebremichael-Weldeselassie Y, Man KKC, Lau KK, Lam ICH, Lai FTT, Li X, Wong CKH, Chan EW, Cheung CL, Sing CW, Lee CK, Hung IFN, Lau CS, Chan JYS, Lee MK, Mok VCT, Siu CW, Chan LST, Cheung T, Chan FLF, Leung AY, Cowling BJ, Leung GM, Wong ICK. Thromboembolic events and hemorrhagic stroke after mRNA (BNT162b2) and inactivated (CoronaVac) covid-19 vaccination: A self-controlled case series study. *EClinicalMedicine* 2022;**50**:101504–101504.

207. Tokarek T, Dziewierz A, Malinowski KP, Rakowski T, Bartus S, Dudek D, Siudak Z. Treatment delay and clinical outcomes in patients with st-segment elevation myocardial infarction during the covid-19 pandemic. *J Clin Med* 2021;**10**.

208. Raisi-Estabragh Z, McCracken C, Cooper J, Fung K, Paiva JM, Khanji MY, Rauseo E, Biasiolli L, Raman B, Piechnik SK, Neubauer S, Munroe PB, Harvey NC, Petersen SE. Adverse cardiovascular magnetic resonance phenotypes are associated with greater likelihood of incident coronavirus disease 2019: findings from the UK Biobank. *Aging Clin Exp Res* 2021;**33**:1133–1144.

209. Dogan Z, Erden I, Bektasoglu G, Karabulut A. Association Between History of Polymerase Chain Reaction-verified COVID-19 Infection and Outcomes of Subsequent ST-Elevation Myocardial Infarction. *Angiology* 2024;**75**:131–138.

210. Wong AYS, Tomlinson L, Brown JP, Elson W, Walker AJ, Schultze A, Morton CE, Evans D, Inglesby P, MacKenna B, Bhaskaran K, Rentsch CT, Powell E, Williamson E, Croker R, Bacon S, Hulme W, Bates C, Curtis HJ, Mehrkar A, Cockburn J, McDonald HI, Mathur R, Wing K, Forbes H, Eggo RM, Evans SJW, Smeeth L, Goldacre B, Douglas IJ. Association between oral anticoagulants and COVID-19-related outcomes: a population-based cohort study. *Br J Gen Pract* 2022;**72**:e456–e463.

211. Mendel A, Fritzler M, St Pierre Y, Rauch J, Bernatsky S, Vinet E. Association of antiphospholipid antibodies with thromboembolic events and severe outcomes in COVID-19. *Arthritis Rheumatol* 2021;**73**:2010–2013.

212. Horne BD, Muhlestein JB, May HT, Le VT, Bair TL, Knowlton KU, Anderson JL. Association of Periodic Fasting with Lower Severity of COVID-19 Outcomes in the SARS-CoV-2 Pre-Vaccine Era: An Observational Cohort from the INSPIRE Registry. *medRxiv* 2022;**20**.

213. Bryant JM, Boncyk CS, Rengel KF, Doan V, Snarskis C, McEvoy MD, McCarthy KY, Li G, Sandberg WS, Freundlich RE. Association of Time to Surgery after COVID-19 Infection with Risk of Postoperative Cardiovascular Morbidity. *JAMA Netw Open* 2022;**5**:e2246922.

214. Ollila H, Pihlajamaa J, Martola J, Kuusela L, Blennow K, Zetterberg H, Salmela V, Hokkanen L, Tiainen M, Hästbacka J. Brain magnetic resonance imaging findings six months after critical COVID-19: A prospective cohort study. *J Crit Care* 2024;**80**.

215. Kolin DA, Kulm S, Christos PJ, Elemento O. Clinical, regional, and genetic characteristics of Covid-19 patients from UK Biobank. *PLoS ONE Electron Resour* 2020;**15**:e0241264.

216. Horvath S, Archampong D, Nagaraja P, Roberts G, Stephens M, Fialova J, Griffin S, Chavez R, Asderakis A, Kaposztas Z. Complications of kidney transplantation from donors after cardiac death (DCD). *Transpl Int* 2011;**2**:289.

217. Obel N, Fox MP, Tetens MM, Pedersen L, Krause TG, Ullum H, Sorensen HT. Confounding and Negative Control Methods in Observational Study of SARS-CoV-2 Vaccine Effectiveness: A Nationwide, Population-Based Danish Health Registry Study. *Clin Epidemiol* 2024;**16**:501–512.

218. Davis MG, Gangu K, Suriya S, Maringanti BS, Chourasia P, Bobba A, Tripathi A, Avula SR, Shekhar R, Sheikh AB. COVID-19 and Acute Ischemic Stroke Mortality and Clinical Outcomes among Hospitalized Patients in the United States: Insight from National Inpatient Sample. *J Clin Med* 2023;**12**:1340–1340.

219. Courivaud C, Bamoulid J, Chalopin JM, Gaiffe E, Tiberghien P, Saas P, Ducloux D. Cytomegalovirus exposure and cardiovascular disease in kidney transplant recipients. *J Infect Dis* 2013;**207**:1569–1575.

220. Petrakopoulou P, Kubrich M, Pehlivanli S, Meiser B, Reichart B, Scheidt W von, Weis M. Cytomegalovirus infection in heart transplant recipients is associated with impaired endothelial function. *Circulation* 2004;**110**:II207-12.

221. MacIntyre R, Dwyer D, Lindley R, Gao Z, Tan T, Seale H, Heywood A, Ridda I, Kovoor P, Mitchell P. Does influenza precipitate acute ischaemic heart disease? A prospective case control study. *Int J Infect Dis* 2012;**16**:e352.

222. Reunanen A, Roivainen M, Kleemola M, Saikku P, Leinonen M, Hovi T, Knekt P, Leino A, Aromaa A. Enterovirus, mycoplasma and other infections as predictors for myocardial infarction. *J Intern Med* 2002;**252**:421–429.

223. Hammond ME, Christensen ED, Belenky M, Snow GL, Shah K, Hammond MEH. Evidence of autoinflammation as a principal mechanism of myocardial injury in SARS-CoV-2 PCR-positive medical examiner cases. *Diagn Pathol* 2023;**18**:114.

224. Ghani ZH, Mohammed AH. Human Cytomegalovirus and Heart Diseases: A Case Control Study. *Biochem Cell Arch* 2021;**21**:2507–2511.

225. Das S, Ray BK, Ghosh R, Sengupta S, Pandit A, Dubey S. Impact of COVID-19 pandemic in natural course of Moyamoya Angiopathy: an experience from tertiary-care-center in India. *Egypt J Neurol Psychiatry Neurosurg* 2021;**57**:166.

226. Rivera-Izquierdo M, Lainez-Ramos-Bossini AJ, Alba IGF de, Ortiz-Gonzalez-Serna R, Serrano-Ortiz A, Fernandez-Martinez NF, Ruiz-Montero R, Cervilla JA. Long COVID 12 months after discharge: persistent symptoms in patients hospitalised due to COVID-19 and patients hospitalised due to other causes-a multicentre cohort study. *BMC Med* 2022;**20**:92.

227. Tabarelli W, Bonatti H, Tabarelli D, Eller M, Muller L, Ruttmann E, Lass-Florl C, Larcher C, Geltner C. Long term complications following 54 consecutive lung transplants. *J Thorac Dis* 2016;**8**:1234–1244.

228. Gronkjaer CS, Christensen RHB, Kondziella D, Benros ME. Long-term neurological outcome after COVID-19 using all SARS-CoV-2 test results and hospitalisations in Denmark with 22-month follow-up. *Nat Commun* 2023;**14**.

229. Mehta L, Gupta RK, Gupta P. MRI findings in COVID-19 positive and COVID-19 negative patients presenting with acute neurological symptoms-a case-control study. *Neurol Asia* 2022;**27**:117–123.

230. Petersen EL, Gossling A, Adam G, Aepfelbacher M, Behrendt CA, Cavus E, Cheng B, Fischer N, Gallinat J, Kuhn S, Gerloff C, Koch-Gromus U, Harter M, Hanning U, Huber TB, Kluge S, Knobloch JK, Kuta P, Schmidt-Lauber C, Lutgehetmann M, Magnussen C, Mayer C, Muellerleile K, Munch J, Nagele FL, Petersen M, RennCrossed DST, Riedl KA, Rimmele DL, Schafer I, Schulz H, Tahir E, Waschki B, Wenzel JP, Zeller T, Ziegler A, Thomalla G, Twerenbold R, Blankenberg S. Multi-organ assessment in mainly non-hospitalized individuals after SARS-CoV-2 infection: The Hamburg City Health Study COVID programme. *Eur Heart J* 2022;**43**:1124–1137.

231. Junior WBC, Ferreia NN, Santos LM, Borges PBA, Albuquerque CP de, Espindola LS, Nóbrega OT, Gomes CM, Mota LMH da, Soares A. Negative impact of SARS-CoV-2 infection in acute coronary syndrome mortality in a Latin American cohort study. *Front Med* 2022;**9**:959769.

232. Niccoli G, Severino A, Pieroni M, Cosentino N, Ventrone MA, Conte M, Roberto M, Gallinella G, Liuzzo G, Leone AM, Porto I, Burzotta F, Trani C, Crea F. Parvovirus B19 at the culprit coronary stenosis predicts outcome after stenting. *Eur J Clin Invest* 2014;**44**:209–218.

233. Ferrara A, Hedderson MM, Zhu Y, Avalos LA, Kuzniewicz MW, Myers LC, Ngo AL, Gunderson EP, Ritchie JL, Quesenberry CP, Greenberg M. Perinatal Complications in Individuals in California With or Without SARS-CoV-2 Infection During Pregnancy. *JAMA Intern Med* 2022;**182**:503–512.

234. Nordenskjold AM, Johansson N, Sunnefeldt E, Athlin S, Frobert O. Prevalence and prognostic implications of myocardial injury in patients with influenza. *Eur Heart J Open* 2022;**2**:oeac051.

235. Kažukauskienė I, Baltrūnienė V, Jakubauskas A, Žurauskas E, Maneikienė VV, Daunoravičius D, Čelutkienė J, Ručinskas K, Grabauskienė V. Prevalence and prognostic relevance of myocardial inflammation and cardiotropic viruses in non-ischemic dilated cardiomyopathy. *Cardiol J* 2022;**29**:441–453.

236. Tereshchenko LG, Bishop A, Fisher-Campbell N, Levene J, Morris CC, Patel H, Beeson E, Blank JA, Bradner JN, Coblens M, Corpron JW, Davison JM, Denny K, Earp MS, Florea S, Freeman H, Fuson O, Guillot FH, Haq KT, Kim M, Kolseth C, Krol O, Lin L, Litwin L, Malik A, Mitchell E, Mohapatra A, Mullen C, Nix CD, Oyeyemi A, Rutlen C, Tam AE, Van Buren I, Wallace J, Khan A. Risk of Cardiovascular Events After COVID-19. *Am J Cardiol* 2022;**179**:102–109.

237. Muñoz-Quiles C, López-Lacort M, Urchueguía A, Díez-Domingo J, Orrico-Sánchez A. Risk of Cardiovascular Events After Influenza: A Population-Based Self-Controlled Case Series Study, Spain, 2011–2018. *J Infect Dis* 2024;**230**:e722–e731.

238. Walker TA, Waite B, Thompson MG, McArthur C, Wong C, Baker MG, Wood T, Haubrock J, Roberts S, Gross DK, Sue Huang Q, Claire Newbern E. Risk of severe influenza among adults with chronic medical conditions. *J Nutr* 2020;**150**:183–190.

239. Gorayeb-Polacchini FS, Caldas HC, Bottazzo AC, Abbud-Filho M. SARS-CoV-2 assessment in an outpatient dialysis facility of a single center in Brazil. *Braz J Infect Dis* 2021;**25**.

240. Thoppil JJ, Courtney DM, McDonald S, Kabrhel C, Nordenholz KE, Camargo CA Jr, Kline JA. SARS-CoV-2 Positivity in Ambulatory Symptomatic Patients Is Not Associated With Increased Venous or Arterial Thrombotic Events in the Subsequent 30 Days. *J Emerg Med* 2022;**62**:716–724.

241. Glinge C, Engstrom T, Midgley SE, Tanck MWT, Halkjaer Madsen JE, Pedersen F, Jacobsen MR, Lodder EM, Al-Hussainy NR, Stampe NK, Trebbien R, Kober L, Gerds T, Torp-Pedersen C, Fischer TK, Bezzina CR, Tfelt-Hansen J, Jabbari R. Seasonality of ventricular fibrillation at first myocardial infarction and association with viral exposure. *PLoS ONE* 2020;**15**:e0232569.

242. Diaz-Dominguez KA, Cruz-Melendez A, Amezcua-Castillo LM, Guerra-Lopez JA, Tavera-Alonso C, Gonzalez-Pacheco H, Amezcua-Guerra LM. Should a recent SARS-CoV-2 infection be considered a risk or prognostic factor for ST-segment elevation myocardial infarction? *Arch Cardiol Mex* 2022;**08**.

243. Saleem M. Study the Effect of Seasonal Influenza Virus Infection on Patients with Acute Myocardial Infarction and Acute Kidney Injury. *J Al-Nahrain Univ-Sci* 2016;**19**:8–17.

244. Cohnert TU, Stevanov M, Siegl G, Konstantiniuk P. Surgical Revascularization for Acute Mesenteric Ischemia in COVID-19 Patients. *J Vasc Surg* 2022;**75**:e68–e69.

245. Isrctn. Telomerase activator TA-65MD® in patients with ACS (TACTIC). *https://trialsearch.who.int/Trial2.aspx?TrialID=ISRCTN16613292* 2018.

246. Turner KM, Lee HC, Boppana SB, Carlo WA, Randolph DA. The incidence and impact of CMV infection in preterm infants. *J Investig Med* 2013;**61**:512–513.

247. Pincavitch JD, Pisquiy JJ, Wen S, Bryan N, Ammons J, Makwana P, Dietz MJ. Thirty-Day Mortality and Complication Rates in Total Joint Arthroplasty After a Recent COVID-19 Diagnosis: A Retrospective Cohort in the National COVID Cohort Collaborative (N3C). *J Bone Jt Surg - Am Vol* 2023;**105**:1362–1372.

248. Mohammad S, Amendola M, Banc-Husu A, Chapin C, Cheng K, Chu C, Cohen M, Diamond T, Gold B, Goldstein M, Gumm A, Gupta NA, Karpen SJ, Kohut T, Kriegermeier A, Mackling J, Raghu V, Wadera S, Wood P, Kohli R. Worldwide Outbreak of Acute Hepatitis in Children: Interim Results from the Severe Hepatitis in Pediatric Patients (Shipp) International Registry. *Hepatology* 2023;**77**:e116–e117.

249. Alshammari H, Ali S, Galluzza D, Rechester O, Nouman R, Gandhi C, Mayer S, Al-Muft F. Acute cerebrovascular disorders and vasculopathies associated with significant mortality in SARS-CoV-2 patients admitted to the intensive care unit in the New York Epicenter. *Neurol Conf 73rd Annu Meet Am Acad Neurol AAN* 2021;**96**.

250. Sheth KN, Mazurek MH, Yuen MM, Cahn BA, Shah JT, Ward A, Kim JA, Gilmore EJ, Falcone GJ, Petersen N, Gobeske KT, Kaddouh F, Hwang DY, Schindler J, Sansing L, Matouk C, Rothberg J, Sze G, Siner J, Rosen MS, Spudich S, Kimberly WT. Assessment of Brain Injury Using Portable, Low-Field Magnetic Resonance Imaging at the Bedside of Critically Ill Patients. *JAMA Neurol* 2020;**08**:08.

251. Yassine IA, Hussein MM, Hosny AO, ElSamahy MA. Association between acute stroke and COVID-19 infection among patients with acute stroke. *Egypt J Neurol Psychiatry Neurosurg* 2024;**60**.

252. Pensato U, Forlivesi S, Gentile M, Romoli M, Muccioli L, Ambrosi F, Foschini MP, Gallo C, Ballestrazzi MS, Teutonico P, Faggioli G, Gargiulo M, Galluzzo S, Taglialatela F, Simonetti L, Zini A. Carotid free-floating thrombus in COVID-19: a cerebrovascular disorder of cytokine storm-related immunothrombosis. *Neurol Sci* 2023;**44**:1855–1860.

253. Muhammad AS, Ammar A, Khan B, Qayyum D, Bhatti UH, Hassan M, Ashok A, Saghir T, Ali F, Salahuddin N. Clinical Memory of Covid-19 Pandemic: A Comparative Analysis of Clinical and Angiographic Characteristics of Patients with Acute Coronary Syndrome. *Pak Heart J* 2023;**56**:101–109.

254. Ates O, Yilmaz I, Karaarslan N, Ersoz E, Hacioglu Kasim FB, Dogan M, Ozbek H. Coexistence of SARS-CoV-2 and cerebrovascular diseases: Does COVID-19 positivity trigger cerebrovascular pathologies? *J Infect Dev Ctries* 2022;**16**:981–992.

255. Aykac O, Ozdemir AO, Giray S, Akpinar CK, Ozkul A, Ozdemir G, Sarionder Gencer E, Gurkas E, Acar BA, Yildirim S, Kocaturk O, Yurekli VA, Milanlioglu A, Sair A, Inanc Y, Cabalar M, Sengeze N, Bilgic AB, Acar T, Ersoy AN, Onalan A, Baydemir R. Comparison of COVID-19 patients who underwent thrombectomy with those in the pre-pandemic period in terms of etiology and prognosis. *Eur Rev Med Pharmacol Sci* 2022;**26**:4884–4892.

256. Mehrpour M, Shuaib A, Farahani M, Hatamabadi HR, Fatehi Z, Ghaffari M, Moghadam NB, Aghamiri SH, Mansouri B, Assarzadegan F, Lima BS, Hesami O. Coronavirus disease 2019 and stroke in Iran: a case series and effects on stroke admissions. *Int J Stroke* 2021;**16**:1047–1052.

257. Nicholls AC, Thomas M. COXSACKIE VIRUS INFECTION IN ACUTE MYOCARDIAL INFARCTION. *The Lancet* 1977;**309**:883–884.

258. Kwee RM, Krdzalic J, Fasen B, Jaegere TMH de. CT Scanning in Suspected Stroke or Head Trauma: Is it Worth Going the Extra Mile and Including the Chest to Screen for COVID-19 Infection? *Am J Neuroradiol* 2020;**41**:1165–1169.

259. Alhejily W. Impact of the COVID-19 Pandemic on Patients With Acute Coronary Syndrome: A Tertiary Center Experience With Primary Percutaneous Intervention and Early Invasive Strategy. *Cureus* 2021;**13**:e20747.

260. Baytugan NZ, Kandemir HC, Bezgin T. In-Hospital Outcomes of ST-Segment Elevation Myocardial Infarction in COVID-19 Positive Patients Undergoing Primary Percutaneous Intervention. *Arq Bras Cardiol* 2024;**121**:e20230258.

261. Bayar MD, Sisman AB, Koral G, Cirak S, Tuzun E, Gunaydin S, Bastan B. Increased Serum Citrullinated Histone H3 Levels in Covid-19 Patients with Acute Ischemic Stroke. *Ideggyogyaszati Szle* 2022;**75**:191–198.

262. Beslow LA, Agner SC, Santoro JD, Ram D, Wilson JL, Harrar D, Appavu B, Fraser SM, Rossor T, Torres MD, Kossorotoff M, Zuniga Zambrano YC, Hernandez-Chavez M, Hassanein SMA, Zafeiriou D, Dowling MM, Kopyta I, Stence NV, Bernard TJ, Dlamini N. International Prevalence and Mechanisms of SARS-CoV-2 in Childhood Arterial Ischemic Stroke during the COVID-19 Pandemic. *Stroke* 2022;**53**:2497–2503.

263. Khazaal F, Okleh N, Ahmad F, Baroudy G, Omosola A, Alsehli Z, El Hassan M, Alataresh E, Al-Suwaidi J, Al-Qahtani A, Asaad N, Rafie I, Arabi A. Isolation Unit: A New Way to Practice at Heart Hospital, Qatar during the Corona Virus -2019 Pandemic. *Heart Views* 2020;**21**:157–160.

264. Blackburn R, Zhao H, Pebody R, Hayward A, Warren-Gash C. Laboratory-Confirmed Respiratory Infections as Predictors of Hospital Admission for Myocardial Infarction and Stroke: Time-Series Analysis of English Data for 2004-2015. *Clin Infect Dis* 2018;**67**:8–17.

265. Wood A, Denholm R, Hollings S, Cooper J, Ip S, Walker V, Denaxas S, Akbari A, Banerjee A, Whiteley W, Lai A, Sterne J, Sudlow C. Linked electronic health records for research on a nationwide cohort of more than 54 million people in England: data resource. *BMJ* 2021;**373**:n826.

266. Arevalos V, Ortega-Paz L, Fernandez-Rodriguez D, Alfonso Jimenez-Diaz V, Rius JB, Campo G, Rodriguez-Santamarta M, Prado AP de, Gomez-Menchero A, Diaz Fernandez JF, Scardino C, Gonzalo N, Pernigotti A, Alfonso F, Jesus Amat-Santos I, Silvestro A, Ielasi A, Maria de la Torre J, Bastidas G, Gomez-Lara J, Sabate M, Brugaletta S. Long-term effects of coronavirus disease 2019 on the cardiovascular system, CV COVID registry: A structured summary of a study protocol. *PLoS ONE* 2021;**16**:e0255263.

267. Alasnag M, Ahmed W, Bokhari F, Al-Shaibi K. Management of Acute Coronary Syndrome During the MERS-CoV Outbreak - Single-Center Experience. *Cardiovasc Revasc Med* 2021;**24**:20–23.

268. Kahwagi J, Mbodj A, Diagne R, Toure K, Ndiaye M, Diop A. NEUROCOVID: Experience of a Sub-Saharan African country, example of Senegal. *World Congress of Neurology*. Rome, Italy: Journal of the Neurological Sciences; 2021. p429 Supplement.

269. Vejpongsa P, Kitkungvan D, Madjid M, Charitakis K, Anderson HV, Arain SA, Balan P, Smalling RW, Dhoble A. Outcomes of Acute Myocardial Infarction in Patients with Influenza and Other Viral Respiratory Infections. *Am J Med* 2019;**132**:1173–1181.

270. Porter DD, Porter HG. Respiratory viral antigens in autopsy lung tissue specimens from patients with cancer or myocardial infarction. *Clin Infect Dis* 1999;**29**:437–440.

271. Asteggiano F, Divenuto I, Ajello D, Gennaro N, Santonocito O, Marcheselli S, Balzarini L, Nuzzi NP, Politi LS. Stroke management during the COVID-19 outbreak: challenges and results of a hub-center in Lombardy, Italy. *Neuroradiology* 2021;**63**:1087–1091.

272. Sharma P, Shah K, Loomba J, Patel A, Mallawaarachchi I, Blazek O, Ratcliffe S, Breathett K, Johnson AE, Taylor AM, Salerno M, Ragosta M, Sodhi N, Addison D, Mohammed S, Bilchick KC, Mazimba S. The impact of COVID-19 on clinical outcomes among acute myocardial infarction patients undergoing early invasive treatment strategy. *Clin Cardiol* 2022;**45**:1070–1078.

273. Jablonska A, Chmiel M, Chwarscianek N, Czyzewski K, Richert-Przygonska M. Thromboembolic complications associated with COVID-19 infection in children. *Acta Haematol Pol* 2023;**54**:43–45.

274. Fullerton HJ, Di Germanio C, Hills NK, Beslow LA, Cummings DD, Dlamini N, Fox CK, Sun LR, Norris P. Assessing the Impact of the COVID-19 Pandemic on Childhood Arterial Ischemic Stroke: An Unanticipated Natural Experiment. *Stroke Conf Am Stroke Assoc* 2024;**55**.

275. Wang M, Zhang H, He Y, Qin C, Liu X, Liu M, Tang Y, Li X, Yang G, Liang G, Xu S, Wang W. Association Between Ischemic Stroke and COVID-19 in China: A Population-Based Retrospective Study. *Front Med* 2022;**8**:792487.

276. Ntaios G, Michel P, Georgiopoulos G, Guo Y, Li W, Xiong J, Calleja P, Ostos F, Gonzalez-Ortega G, Fuentes B, Alonso de Lecinana M, Diez-Tejedor E, Garcia-Madrona S, Masjuan J, DeFelipe A, Turc G, Goncalves B, Domigo V, Dan GA, Vezeteu R, Christensen H, Christensen LM, Meden P, Hajdarevic L, Rodriguez-Lopez A, Diaz-Otero F, Garcia-Pastor A, Gil-Nunez A, Maslias E, Strambo D, Werring DJ, Chandratheva A, Benjamin L, Simister R, Perry R, Beyrouti R, Jabbour P, Sweid A, Tjoumakaris S, Cuadrado-Godia E, Campello AR, Roquer J, Moreira T, Mazya MV, Bandini F, Matz K, Iversen HK, Gonzalez-Duarte A, Tiu C, Ferrari J, Vosko MR, Salzer HJF, Lamprecht B, Dunser MW, Cereda CW, Quintero ABC, Korompoki E, Soriano-Navarro E, Soto-Ramirez LE, Castaneda-Mendez PF, Bay-Sansores D, Arauz A, Cano-Nigenda V, Kristoffersen ES, Tiainen M, Strbian D, Putaala J, Lip GYH. Characteristics and Outcomes in Patients With COVID-19 and Acute Ischemic Stroke: The Global COVID-19 Stroke Registry. *Stroke* 2020;**51**:e254–e258.

277. Muller SA, Manintveld OC, Szymanski MK, Damman K, Meer MG van der, Caliskan K, Laake LW van, Oerlemans MIFJ. Characteristics and outcomes of COVID-19 in heart transplantation recipients in the Netherlands. *Neth Heart J* 2022;**30**:519–525.

278. Chuaychoo B, Ngamwongwan S, Kaewnaphan B, Athipanyasilp N, Horthongkham N, Kantakamalakul W, Muangman N. Clinical manifestations and outcomes of respiratory syncytial virus infection in adult hospitalized patients. *J Clin Virol* 2019;**117**:103–108.

279. Bektas M, Ay M, Hamdi Uyar M, Ikbal Kilic M. Combination therapy of high-dose intravenous anakinra and baricitinib in patients with critical COVID-19: Promising results from retrospective observational study. *Int Immunopharmacol* 2024;**129**:111586.

280. Chou OHI, Chung CT, Satti DI, Zhou J, Lee TTL, Wai AKC, Liu T, Lee S, Vassiliou VS, Cheung BMY, Tse G. Comparisons of the rate of acute myocardial infarction between COVID-19 patients and individuals received COVID-19 vaccines: a population-based study. *medRxiv* 2022;**25**.

281. Pomnikov VG, Sakovskiy IV, Yashchikhina TA, Dudkina OV. [COVID-19 and cerebral stroke]. *Zh Nevrol Psikhiatr Im S S Korsakova* 2021;**121**:5–8.

282. Terlecki M, Wojciechowska W, Klocek M, Olszanecka A, Bednarski A, Drozdz T, Pavlinec C, Lis P, Zajac M, Rusinek J, Siudak Z, Bartus S, Rajzer M. Impact of concomitant COVID-19 on the outcome of patients with acute myocardial infarction undergoing coronary artery angiography. *Front Cardiovasc Med* 2022;**9**.

283. Havenon A de, Ney JP, Callaghan B, Delic A, Hohmann S, Shippey E, Esper GJ, Stulberg E, Tirschwell D, Frontera J, Yaghi S, Anadani M, Majersik JJ. Impact of COVID-19 on Outcomes in Ischemic Stroke Patients in the United States. *J Stroke Cerebrovasc Dis* 2021;**30**:105535.

284. Jain SS, Liu Q, Raikhelkar J, Fried J, Elias P, Poterucha TJ, DeFilippis EM, Rosenblum H, Wang EY, Redfors B, Clerkin K, Griffin JM, Wan EY, Abdalla M, Bello NA, Hahn RT, Shimbo D, Weiner SD, Kirtane AJ, Kodali SK, Burkhoff D, Rabbani LE, Schwartz A, Leon MB, Homma S, Di Tullio MR, Sayer G, Uriel N, Anstey DE. Indications for and Findings on Transthoracic Echocardiography in COVID-19. *J Am Soc Echocardiogr* 2020;**33**:1278–1284.

285. Bainton D, Jones GR, Hole D. Influenza and Ischaemic Heart Disease-a Possible Trigger for Acute Myocardial Infarction? *Int J Epidemiol* 1978;**7**:231–239.

286. Eligulashvili A, Gordon M, Lee JS, Lee J, Mehrotra-Varma S, Mehrotra-Varma J, Hsu K, Hilliard I, Lee K, Li A, Essibayi MA, Yee J, Altschul DJ, Eskandar E, Mehler MF, Duong TQ. Long-term outcomes of hospitalized patients with SARS-CoV-2/COVID-19 with and without neurological involvement: 3-year follow-up assessment. *PLoS Med* 2024;**21**:e1004263.

287. Pinzon RT, Veronica V. Medical comorbidities as predictors of COVID-19 short-term mortality: A historical cohort study in Indonesia. *Tzuchi Med J* 2023;**35**:53–57.

288. Gombolay G, Anderson M, Xiang Y, Bai S, Rostad CA, Tyor W. Neurologic complications in children with seizures and respiratory illness: A comparison between SARS-CoV-2 and other respiratory viruses. *J Clin Transl Sci* 2022;**6**:38–39.

289. Navar AM, Cosmatos I, Purinton S, Ramsey JL, Taylor RJ, Sobel RE, Barlow G, Dieck GS, Bulgrein ML, Peterson ED. Using EHR data to identify coronavirus infections in hospitalized patients: Impact of case definitions on disease surveillance. *Int J Med Inf* 2022;**166**:104842.

290. Modin D, Claggett B, Sindet-Pedersen C, Lassen MCH, Skaarup KG, Jensen JS, Fralick M, Schou M, Lamberts M, Gerds TA, Fosbøl EL, Phelps M, Kragholm K, Andersen MP, Køber L, Torp-Pedersen C, Solomon SD, Gislason G, Biering-Sørensen T. Acute COVID-19 and the Incidence of Ischemic Stroke and Acute Myocardial Infarction. *Circulation* 2020;**142**:2080–2082.

291. Grist NR, Bell E. Coxsackie viruses and myocardial infarction. *The Lancet* 1977;**310**:41–41.

292. Glück T. Do Pneumococcal and Respiratory Virus Infections Trigger Cardiovascular Morbidity. *NEJM Journal Watch*. https://www.jwatch.org/na46451/2018/04/09/do-pneumococcal-and-respiratory-virus-infections-trigger

293. Arévalos V, Ortega-Paz L, Brugaletta S. Mid-term effects of SARS-CoV-2 infection on cardiovascular outcomes. *Med Clin Engl Ed* 2022;**158**:41–42.

294. Lassen MCH, Modin D, Skaarup KG, Johansen ND, Claggett B, Solomon SD, Fralick M, Jensen JUS, Sivapalan P, Vaduganathan M, Pareek M, Schou M, Krause TG, Hviid A, Køber L, Torp-Pedersen C, Gislason G, Biering-Sørensen T. Risk of Incident Thromboembolic and Ischemic Events After COVID-19 Vaccination Compared With SARS-CoV-2 Infection. *Circulation*.

295. Corrales-Medina VF, Madjid M, Musher DM. Role of acute infection in triggering acute coronary syndromes. *Lancet Infect Dis* 2010;**10**:83–92.

296. Abbasi J. The COVID Heart-One Year After SARS-CoV-2 Infection, Patients Have an Array of Increased Cardiovascular Risks. *JAMA* 2022;**327**:1113–1113.

297. Higgins JPT, Morgan RL, Rooney AA, Taylor KW, Thayer KA, Silva RA, Lemeris C, Akl EA, Bateson TF, Berkman ND, Glenn BS, Hróbjartsson A, LaKind JS, McAleenan A, Meerpohl JJ, Nachman RM, Obbagy JE, O’Connor A, Radke EG, Savović J, Schünemann HJ, Shea B, Tilling K, Verbeek J, Viswanathan M, Sterne JAC. A tool to assess risk of bias in non-randomized follow-up studies of exposure effects (ROBINS-E). *Environ Int* 2024;**186**:108602.

298. Page MJ, McKenzie JE, Bossuyt PM, Boutron I, Hoffmann TC, Mulrow CD, Shamseer L, Tetzlaff JM, Akl EA, Brennan SE, Chou R, Glanville J, Grimshaw JM, Hróbjartsson A, Lalu MM, Li T, Loder EW, Mayo-Wilson E, McDonald S, McGuinness LA, Stewart LA, Thomas J, Tricco AC, Welch VA, Whiting P, Moher D. The PRISMA 2020 statement: an updated guideline for reporting systematic reviews. *Br Med J* 2021;**372**:n71.

299. Stroup DF, Berlin JA, Morton SC, Olkin I, Williamson GD, Rennie D, Moher D, Becker BJ, Sipe TA, Thacker SB, Meta-analysis of Observational Studies in Epidemiology (MOOSE) group. Meta-analysis of Observational Studies in Epidemiology: A Proposal for Reporting. *JAMA* 2000;**283**:2008.
